# Supplementary material for: SMC Progressively Aligns Chromosomal Arms in Caulobacter crescentus but Is Antagonized by Convergent Transcription
Source: Cell Rep. 2017 Aug 29;20(9):2057–71. doi: 10.1016/j.celrep.2017.08.026 (PMC5583512; doi:10.1016/j.celrep.2017.08.026)
Supplement: Document S1. Supplemental Experimental Procedures, Figures S1–S7, and Tables S1–S3 [file mmc1.pdf]

Cell Reports, Volume *20*

Supplemental Information

SMC Progressively Aligns Chromosomal Arms in *Caulobacter crescentus* but Is Antagonized by Convergent Transcription

Ngat T. Tran, Michael T. Laub, and Tung B.K. Le

SUPPLEMENTARY INFORMATION

SMC progressively aligns chromosomal arms in *Caulobacter crescentus* but is antagonized by convergent transcription

Ngat T. Tran, Michael T. Laub, and Tung B. K. Le

SUPPLEMENTARY FIGURES AND LEGENDS

SUPPLEMENTARY EXPERIMENTAL PROCEDURES

SUPPLEMENTARY TABLES S1-S3

SUPPLEMENTARY REFERENCES

Figure S1

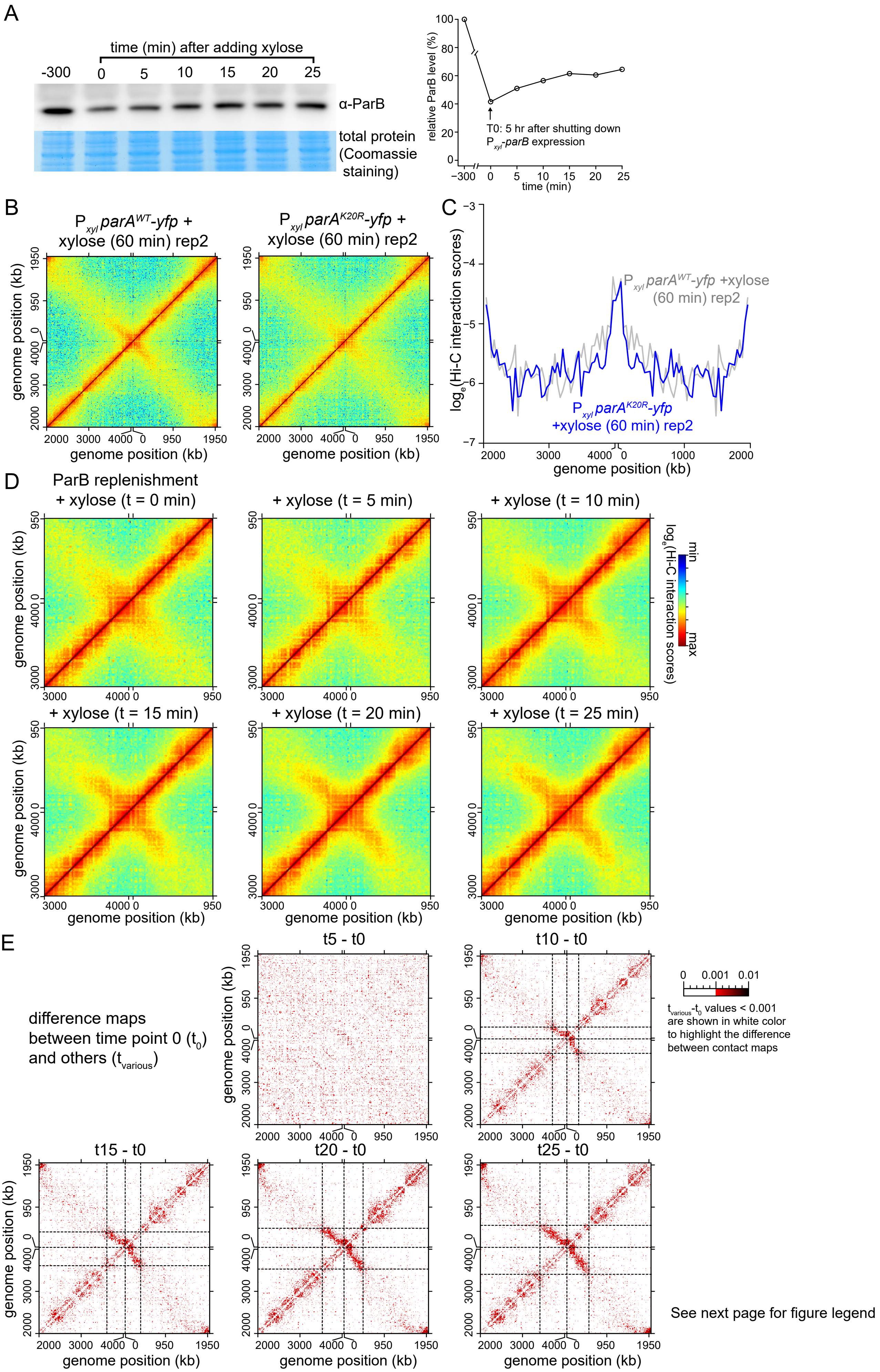

See next page for figure legend

Fig. S1. ParB is required for the progressive alignment of chromosomal arms by SMC, Related to Fig. 2.

(A) Immunoblot analysis of ParB level before the start of the depletion experiment (t = -300 min), at the end of the depletion experiment (t = 0 min), and at time point 5, 10, 15, 20 and 25 minutes after adding back xylose to replenish ParB. **(B)** Normalized Hi-C maps for cells over-expressing *parA*^{WT}*-yfp* (replicate 2) and *parA*^{K20R}*-yfp* (replicate 2) after adding xylose for 1 hr. **(C)** Hi-C interaction scores along the secondary diagonal for contact maps of cells over-expressing *parA*^{WT}*-yfp* (replicate 2) (grey), and *parA*^{K20R}*-yfp* (replicate 2) (blue) after adding xylose for 1 hr. **(D)** A time-resolved Hi-C contact maps for cells that are replenishing of ParB. *parB::*P_{xyl} *parB* cells at the end of the 5 hr depletion period was washed off glucose and supplemented with xylose to induce ParB production. Time (in minutes) after adding back xylose was indicated on top of each Hi-C map. A 1000 kb region surrounding *parS/ori* were zoomed in. **(E)** Hi-C difference maps between time point 0 and others ($t_{various}$ – t_0). The black dotted lines indicate the leading front of the aligned DNA. The rates of progression for each arm of the chromosome are shown in Fig. 2G.

Figure S2

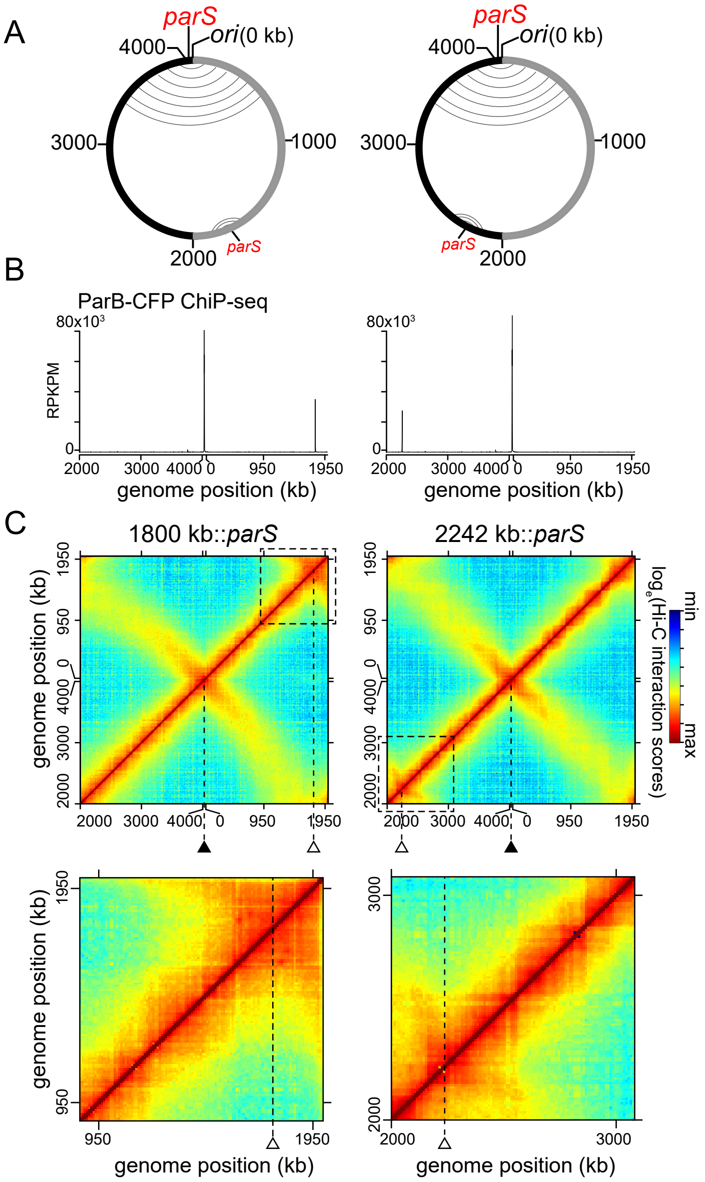

Fig. S2. An ectopic *parS* induces the alignment of its flanking DNA, Related to Fig. 3.

(A) Genomic maps show the locations of a 260 bp DNA fragment containing *parS* site that were engineered at +1800 kb and +2242 kb. Aligned DNA regions are presented as grey curved lines connecting the left and the right flanking of each *parS* site (see also panel **C**). **(B)** ChIP-seq profiles show ParB distribution in cells harbouring a second *parS* site at +1800 kb or at +2242 kb. ChIP-seq using α-GFP antibody were performed in the above cells with *parB*::*cfp-parB* as the sole source of ParB. ChIP-seq enrichment was expressed as number of reads per kilobases per million of mapped reads (RPKPM). **(C)** Normalized Hi-C maps for the +1800 kb::*parS* and +2242 kb::*parS* cells. The solid and open triangles shows the position of the native and ectopic *parS* site, respectively. A 1000 kb region surrounding the ectopic *parS* site (black dashed box) were also zoomed in and presented below each whole-genome Hi-C contact map.

Figure S3

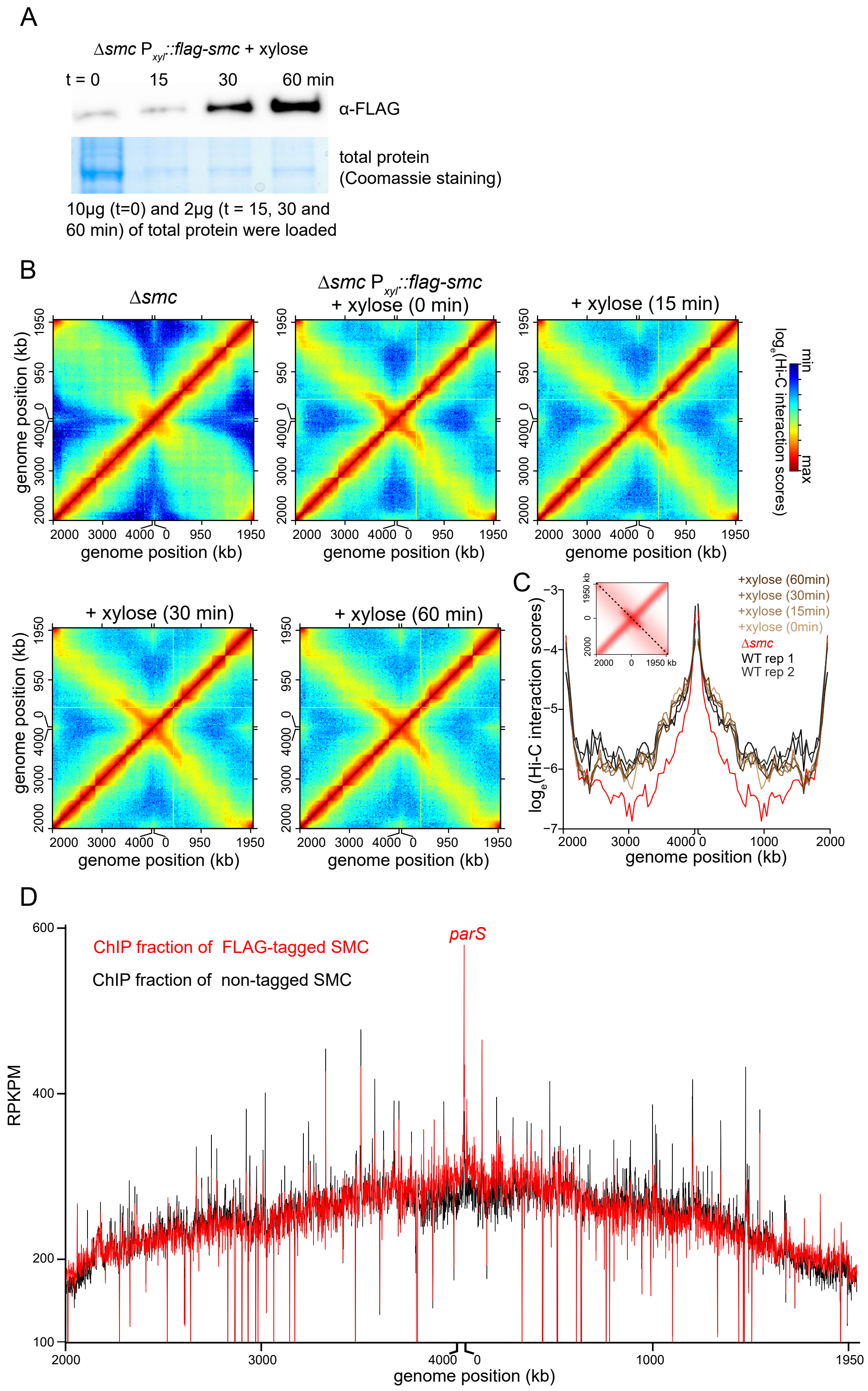

See next page for figure legend

Fig. S3. A FLAG-tagged SMC restores chromosomal arm alignment to *Δsmc Caulobacter*, Related to Fig. 3.

(A) Immunoblot analysis of SMC level at time point 0 (leaky expression), 15, 30, and 60 minutes after adding xylose. For time point t = 0, 10 µg total protein is loaded. For time point t = 15, 30 and 60 min, only 2 µg total protein is loaded to avoid oversaturating the signal on an immunoblot. **(B)** Normalized Hi-C contact maps showing the natural logarithm of DNA-DNA contacts for pairs of 10kb-bins across the genome for *Δsmc* cells (Le et al., 2013), and *Δsmc* P_{xyl}*::flag-smc* after adding xylose for 0, 15, 30 and 60 min. A leaky expression of P_{xyl} (0 min, no added glucose) is sufficient to restore the chromosomal arm alignment. **(C)** Hi-C interaction scores along the secondary diagonal for contact maps of WT, *Δsmc* and for *Δsmc* P_{xyl}*::flag-smc* after adding xylose for 0, 15, 30 and 60 min. **(D)** ChIP fraction (expressed as RPKPM) of *Δsmc* P_{xyl}*::flag-smc* (red) from a ChIP-seq experiment using α-FLAG antibody. ChIP fraction of a non-tagged SMC strain (black) from a ChIP-seq experiment using α-FLAG antibody. Both strains were grown to exponential phase in rich medium at 30°C before fixation with 1% formadehyde and crosslinker Gold.

Figure S4

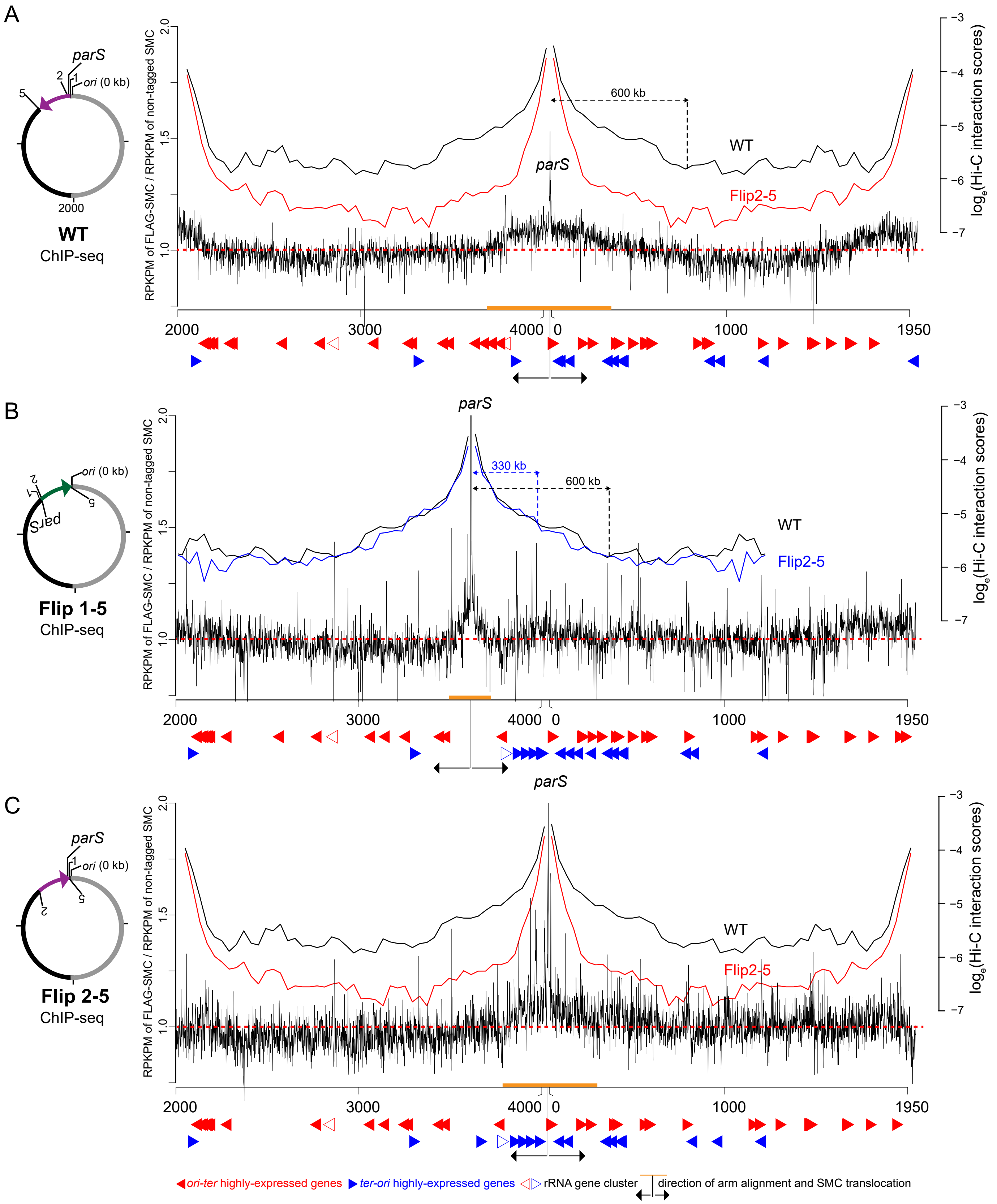

See next page for figure legend

Fig. S4. The genome-wide distribution of FLAG-tagged SMC, Related to Fig. 3 and Fig. 4.

(A) The distribution of FLAG-tagged SMC on WT *Caulobacter* chromosome. DNA from both the α-FLAG ChIP fraction of tagged-SMC and un-tagged SMC were deep sequenced. ChIP-seq signals were reported as the number of reads within every 1 kb bin along the genome in the ChIP fraction of FLAG-tagged SMC divide by that of untagged SMC. The dashed red line shows y-axis value at 1. Below the ChIP-seq profile are the position of highly-expressed genes that transcribe in the *ori-ter* (solid red arrows) or *ter-ori* (solid blue arrows) direction. The positions of rRNA gene cluster are indicated with open red or blue arrows. The direction and extent of SMC translocation from *parS* site were shown as black arrows and orange bar, respectively. High expression genes (RPKPM*gene length > 1000) were determined from α-FLAG ChIP-seq in cells expressing *rpoC-flag* (See Fig. 4A and Fig. S6). A schematic genomic map of *Caulobacter* showing the position of *parS* and *ori* are presented on the left hand side of each ChIP-seq profile. The inverted DNA segment (purple/green arrow) as in the Flip 1-5 or Flip 2-5 strains is indicated together with the end points of the inversion (1, 2, and 5). The aligned DNA regions, as observed by Hi-C, are presented as grey curved lines connecting the left and the right chromosomal arm. Overlaid on the ChIP-seq profile is Hi-C interaction scores along the diagonal from the upper left corner to the lower right corner of each Hi-C contact map for WT (black) and Flip 2-5 (red). **(B)** The distribution of FLAG-tagged SMC on the chromosome of Flip 1-5 *Caulobacter*. Overlaid on the ChIP-seq profile is Hi-C interaction scores along the diagonal from the upper left corner to the lower right corner of each Hi-C contact map for WT (black) and Flip 1-5 (blue). The Hi-C interaction scores along the diagonal for WT contact map was shifted to the same position as for Flip 1-5 map to enable comparison between strains **(C)** The distribution of FLAG-tagged SMC on the chromosome of Flip 2-5 *Caulobacter*. Overlaid on the ChIP-seq profile is Hi-C interaction scores along the diagonal from the upper left corner to the lower right corner of each Hi-C contact map for WT (black) and Flip 2-5 (red).

Figure S5

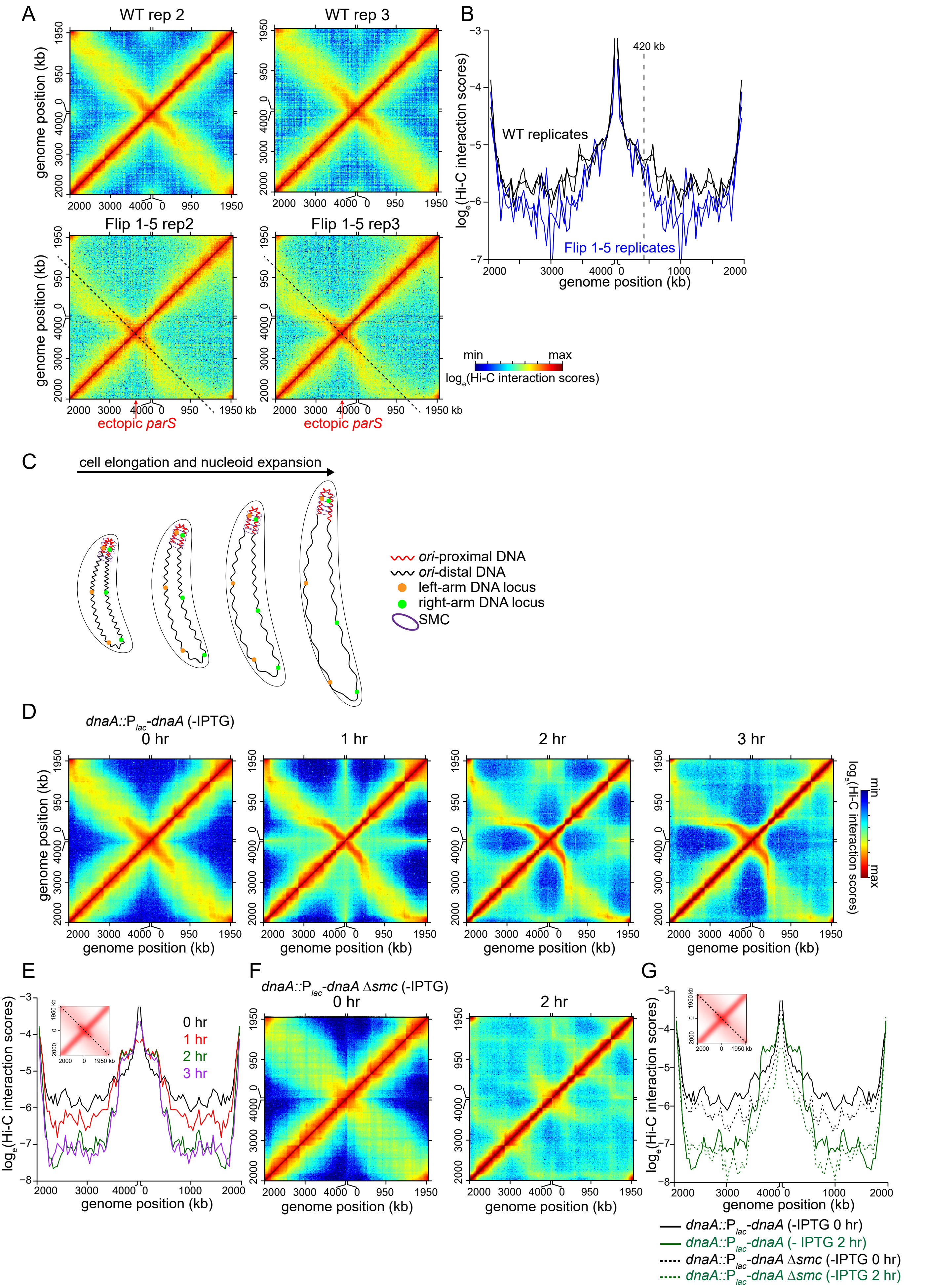

See next page for figure legend

Fig. S5. Hi-C analysis of Flip 1-5 strain and of elongated *Caulobacter* cells, Related to Fig. 3.

(A) Normalized Hi-C maps for the Flip 1-5 (replicate 2-3) and WT cells (replicate 2-3). The genomic position of the relocated *parS* site in Flip 1-5 is indicated with a red arrow. **(B)** Hi-C interaction scores along the diagonal from the upper left corner to the lower right corner for contact maps of WT (black), Flip 1-5 (blue). The Hi-C interaction scores along the secondary diagonal of Flip 1-5 (black dashed lines in panel **A**) was shifted to the same position as that of WT to enable comparison between strains. The vertical black dashed line at ~420 kb away from *ori* shows the position where Hi-C interaction scores along the secondary diagonal start to reduce in Flip 1-5 strain in comparison to WT. **(C)** A schematic presentation of cell elongation and chromosome expansion due to DnaA depletion. Cells depleted of DnaA are arrested in G1 phase but continue to grow, resulting in an elongated morphology with the nucleoid expanding to fill the larger available cytoplasmic volume (Kahng and Shapiro, 2003; Le and Laub, 2016). Pair of DNA loci (orange and green dots) were labeled fluorescently using the orthogonal ParB/*parS* system (Badrinarayanan et al., 2015). SMC, *ori*-proximal DNA and *ori*-distal DNA were labeled as purple eclipse, red and black wavy lines, respectively. **(D)** Hi-C maps for cells depleted of DnaA for the times indicated (Le and Laub, 2016). **(E)** Hi-C interaction scores along the secondary diagonal for Hi-C contact maps of cells depleted of DnaA at various time points. **(F)** Hi-C maps for *Δsmc* cells depleted of DnaA for the times indicated. **(G)** Hi-C interaction scores along the secondary diagonal for Hi-C contact maps of *Δsmc* cells depleted of DnaA at various time points.

Figure S6

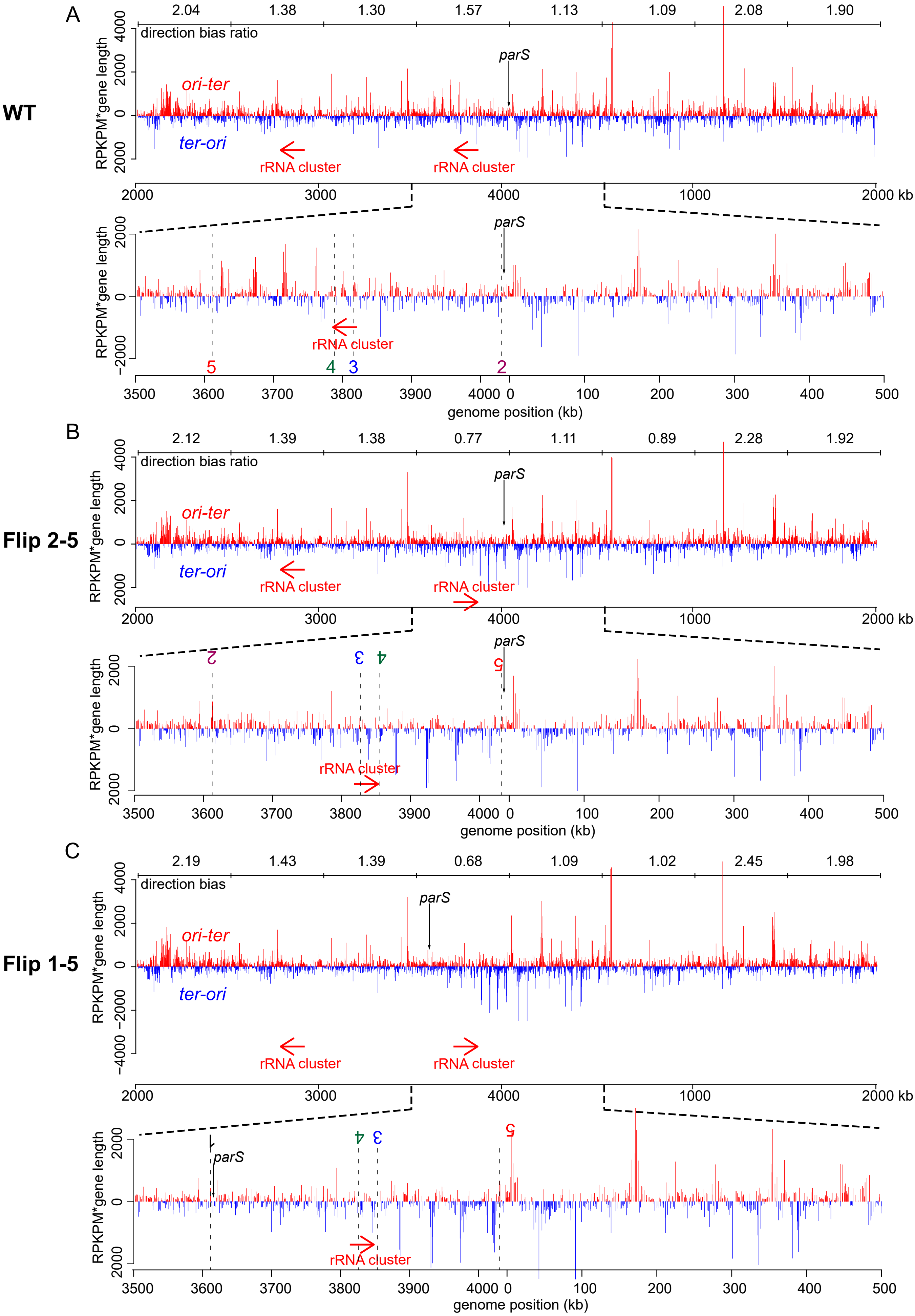

See next page for figure legend

Fig. S6. ChIP-seq of FLAG-tagged RpoC to quantify RNA polymerase abundance and the bias in direction of transcription, Related to Fig. 4.

The abundance of RNA polymerases on genes that are transcribing in the *ori-ter* direction (red) and in the *ter-ori* direction (blue) is shown for WT (Panel **A**), Flip 2-5 (Panel **B**) and Flip 1-5 cells (Panel **C**). Anti-FLAG antibody ChIP-seq was performed on exponentially-growing cells expressing RpoC-FLAG from its native locus. Only pulled-down DNA from translocating RNA polymerases were used to generate this plot. Pulled-down DNA from initiating RNA polymerases at promoter regions were discarded *in silico*. The abundance of RNA polymerases was represented as RPKPM*gene length for each gene and plotted against the genomic location of that gene. The direction bias ratio was calculated as the ratio of RPKPM*gene length for *ori-ter* genes to that of *ter-ori* genes in every 500 kb region along the *Caulobacter* genome. The direction bias ratio (greater than 1) indicates a preference for *ori-ter* transcription throughout the *Caulobacter* genome. Due to short sequencing reads (50 bp) and the high similarity between the two ribosomal RNA clusters, it is not reliable to estimate the RNA polymerase density within each rRNA cluster. Therefore, the enrichment data for rRNA clusters are not shown. Nevertheless, we indicate the genomic positions of highly-expressed rRNA clusters on *Caulobacter* genome with red arrows. A region between +3500 kb and +500 kb was further zoomed in. Vertical black dashed lines with numbering 2, 3, 4 and 5 indicate the inversion end points (See Fig. 3-4 and Fig. 6).

Figure S7

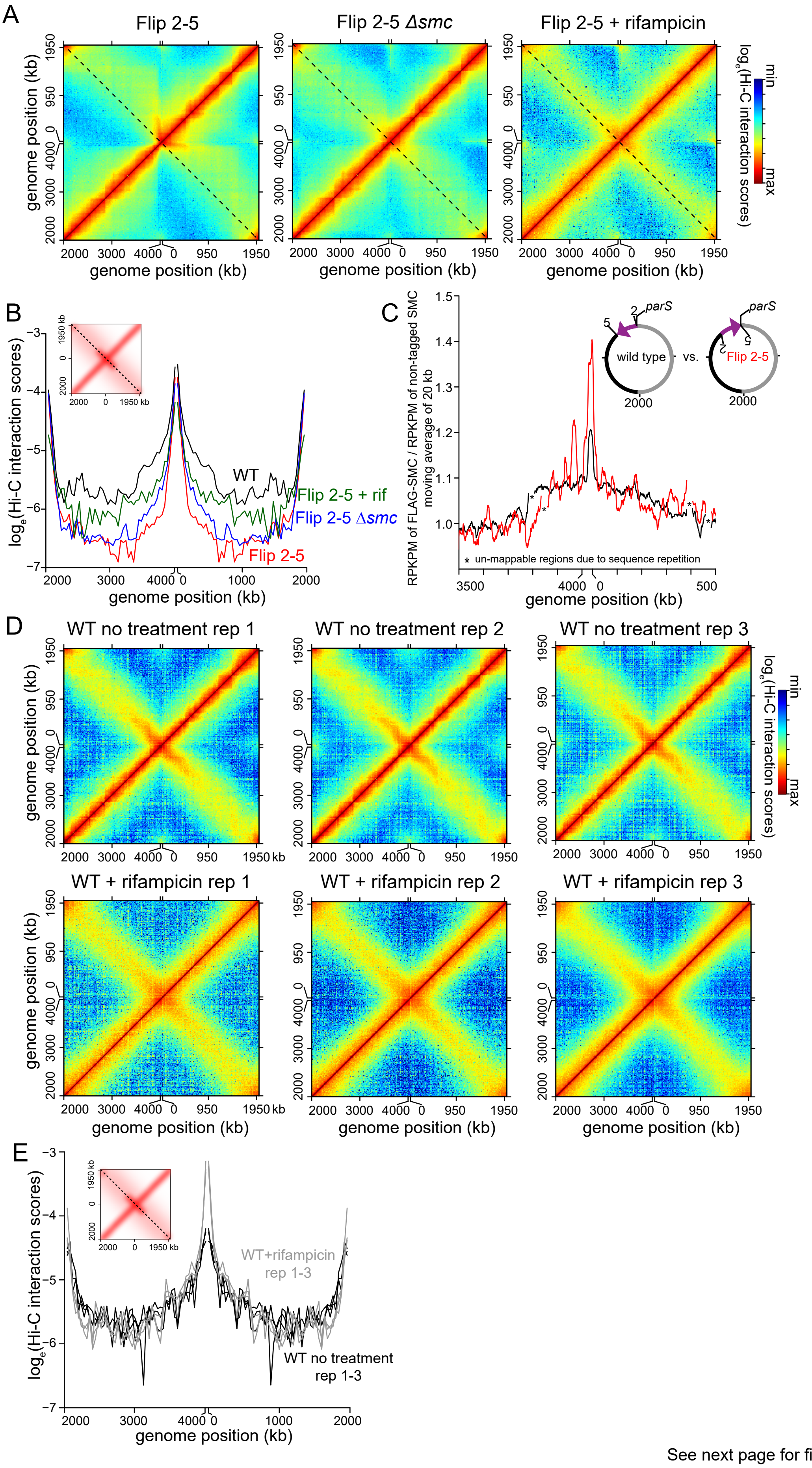

See next page for figure legend

Fig. S7. Hi-C interaction scores along the secondary diagonal for contact maps of the Flip 2-5 strains and the effect of inhibiting transcription elongation on inter-arm interactions in WT cells, Related to Fig. 4 and Fig. 5.

(A) Normalized Hi-C contact maps for Flip 2-5, Flip 2-5 *Δsmc* cells, and Flip 2-5 cells treated with rifampicin (25 µg/ml) for 30 minutes. **(B)** Hi-C interaction scores along the diagonal from the upper left corner to the lower right corner (black dashed line in panel **A**) for contact maps of WT, Flip 2-5, Flip 2-5 *Δsmc*, and Flip 2-5 cells treated with rifampicin (25 µg/ml) for 30 minutes. **(C)** The distribution of FLAG-tagged SMC on WT *Caulobacter* chromosome (black) and on Flip 2-5 chromosome (red). A moving average of twenty 1-kb bins was applied to highlight the difference between the ChIP-seq profiles of WT and Flip 2-5. See also Fig. 6A for profiles without a moving average smoothing. Only DNA segment between +3500 kb and +500 kb was shown. Asterisks (*) indicate the un-mappable DNA regions due to sequence repetition and the short-read nature of Illumina sequencing (50-bp long reads). **(D)** Normalized Hi-C contact maps showing DNA-DNA contacts for pairs of 10kb-bins across the genome of wild-type and cells treated with rifampicin (25 µg/ml) for 30 min. **(E)** Hi-C interaction scores along the secondary diagonal for Hi-C contact maps of wild-type and cells treated with rifampicin (25 µg/ml) for 30 min.

SUPPLEMENTARY EXPERIMENTAL PROCEDURES

Synchronization and growth conditions

Synchronizations of *C. crescentus* were performed on mid-exponential phase cells using Percoll (Sigma) and density gradient centrifugation. Briefly, 250 mL cultures of the wild-type CB15N or its derivatives were grown in PYE at 30°C to an OD_{600} of ~0.4 and pelleted via centrifugation at 8000 *g* for 10 min. Cells were resuspended in 5 ml of 1x M2 salts (6.1 mM Na_2HPO_4, 3.9 mM KH_2PO_4, 9.3 mM NH_4Cl, 0.5 mM $MgSO_4$, 10 µM $FeSO_4$, 0.5 mM $CaCl_2$) via pipetting and 5 mL of ice-cold Percoll was added to the resuspension. The resulting mixture was transferred to a 15 mL Falcon tube that was subsequently centrifuged for 20 min (10000 *g*) at 4°C. G1-phase swarmer cells formed a discrete band near the bottom of the tube. This band was removed via pipetting and the cells within this band were washed three times with 1 ml of ice-cold 1x M2 salts.

After synchronization, swarmer cells were released into PYE+1% formadehyde for fixation for chromosome conformation capture assays (Hi-C).

For antibiotic treatment, swarmer cells were incubated with rifampicin at 25 µg/mL final concentration for 30 minutes before fixing with 1% formadehyde for Hi-C assay.

For xylose induction experiments, xylose was added to mid-exponential phase cultures to 0.3% final concentration. Cultures were incubated with shaking for an additional hour before formadehyde was added to 1% final concentration to fix cells for Hi-C assay.

For the ParB-depletion experiment, MT148 (*parB::*P_{xyl}-*parB*) cells (Thanbichler and Shapiro, 2006) at mid-exponential phase were washed off xylose by repeated centrifugation and resuspension in fresh PYE before releasing to PYE+0.2% glucose. The culture was left shaking at 30°C for five more hours before formadehyde was added to 1% final concentration to fix cells for Hi-C assay.

For the ParB-replenishment experiment, MT148 (*parB::*P_{xyl}-*parB*) cells (Thanbichler and Shapiro, 2006) were first depleted of ParB using the above procedure. At the end of the five-hour period, cells were washed twice in fresh PYE to remove glucose. Xylose (0.3% final concentration) was then added (time point 0). Subsequently, cultures were withdrawn at regular time points (5, 10, 15, 20, and 25 minutes after the addition of xylose) and immediately fixed with 1% formadehyde for Hi-C assays.

Plasmids and Strains construction

All strains used are listed in Supplementary Table S1. All plasmids and primers used in strain and plasmid construction are listed in Supplementary Table S2.

pMCS2-ΔscpA

A 500 bp N-terminal sequence of *scpA* (CCNA_02084) was amplified by PCR using primers scpA_500N_F and scpA_500N-R and purified *Caulobacter* genomic DNA as template. The PCR product was gel-purified and assembled to an NdeI-NheI-cut pMCS2 (Thanbichler et al., 2007) using a 2x Gibson master mix (NEB). Briefly, 2.5 µL of each DNA fragment at equimolar concentration was added to 5 µL Gibson master mix (NEB), and the mixture was incubated at 50°C for 60 minutes. 5 µL was used to transform chemically-competent *E. coli* DH5α cells. Gibson assembly was possible due to a 23 bp sequence shared between the PCR fragment and the NdeI-NheI-cut pMCS2 backbone. The resulting plasmid was sequence verified by Sanger sequencing (Eurofins, Germany).

Electro-competent *Caulobacter* cells were electroporated with pMCS2-*ΔscpA* plasmid to allow for a single integration at *scpA*, thereby inactivating this gene. The correct integration of pMCS2-*ΔscpA* was verified by PCR using primers outside of the homologous regions.

pNPTS138-ΔscpB

A 500 bp upstream of the ScpB-encoding gene (CCNA_02083) that includes the first 36 nucleotides of *scpB* was amplified by PCR using primers scpBfrag1_F and scpBfrag1-R. A 500 bp downstream of the *scpB* including the last 36 nucleotides of *scpB* was amplified by PCR using primers scpBfrag2_F and scpBfrag2_R. PCR products were gel purified and assembled together with a BamHI-EcoRI-cut pNPTS138 using Gibson assembly technique. Briefly, 1.5 µL of each DNA fragment at equimolar concentration was added to 5 µL 2x Gibson master mix (NEB), and the mixture was incubated at 50°C for 60 minutes. 5 µL was used to transform chemically-competent *E. coli* DH5α cells. Gibson assembly was possible due to a 23 bp sequence shared between the three PCR fragments. This homology was incorporated during the primer design to amplify the upstream and downstream region of *scpB*. The resulting plasmid was sequence verified by Sanger sequencing (Eurofins, Germany).

For the deletion of *scpB*, pNPTS138-*ΔscpB* plasmid was first transformed to *Caulobacter* CB15N competent cells via electroporation. A double crossover gene knock-out was performed as described previously (Skerker et al., 2005). The deletion was verified using PCR with primers outside of the homologous flanking regions.

pENTR::smc

The SMC (CCNA_00377) encoding sequence was amplified by PCR from *Caulobacter* genomic DNA using primers smc_pentr_topo_F and smc_pentr_topo_R. The PCR product was gel purified before being ligated to the pENTR backbone in a reaction consisting of 2 µL of gel-purified PCR product, 0.5 µL of D-TOPO reaction buffer, and 0.5 µL of pENTR-D-TOPO vector (Invitrogen) and 2 µL of water. The reaction was incubated for an hour at room temperature before transformation into TOP10 *E. coli* cells (Invitrogen). The resulting plasmid was verified by Sanger dye-termination sequencing (Genewiz, USA).

pML477-smc

The *smc* genes were recombined into a Gateway-compatible destination vector pML477 via LR recombination reaction (Invitrogen). For LR recombination reactions: 1 µL of purified pENTR harbouring *smc* was incubated with 1 µL of the destination vector pML477, 1 µL of LR Clonase II mastermix, and 2 µL of water in a total volume of 5 µL. The reaction was incubated for an hour at room temperature before transformation into DH5α *E. coli* cells. Cells were then plated out on LB agar + spectinomycin. Resulting colonies were restruck onto LB agar + spectinomycin and LB agar + kanamycin. Only colonies that survived on LB + spectinomycin plates were subsequently used for culturing and plasmid extraction.

pNPTS138::inversion insertion point 1 to 6

These plasmids were used to insert a ΦC31 attachment site (*attP* or *attB*) at the intended locations (insertion points) on the *Caulobacter* chromosome. Insertion points are at the end of converging genes so that inverting a chromosome segment is less likely to disrupt transcription promoters near both ends of the inversion. DNA fragments were chemically-synthesized by BioBasics (Canada) to contain: 500 bp on the left hand side of the insertion point + *attP* or *attB* sequence + 500 bp on the right hand side of the insertion point. The sequence of *attP* (50bp, forward direction) is

gtagtgccccaactggggtaacctttgagttctctcagttgggggcgtag. The sequence of *attB* (40bp, reverse direction) is gtacgcgcccggggagcccaagggcacgccctggcacccg. The chemically-synthesized DNA fragments (insertion point 1, 2, 4, 5) were liberated from its carrying plasmid (pUC57, BioBasics) by a double digestion with HindIII and BamHI restriction enzymes. The dropped-out insert was gel-purified before being ligated to a HindIII-BamHI-cut pNPTS138. 10 µL of the ligation reaction was used to transform *E. coli* DH5α cells. The chemically-synthesized DNA fragment (insertion point 3) was liberated from its carrying plasmid (pUC57) by digestion with HindIII and BglII instead. The dropped-out insert was gel-purified before being ligated to a HindIII-BglII cut pNPTS138 backbone. 5 µL of the ligation reaction was used to transform *E. coli* DH5α cells.

pMCS5::parS^{Caulobacter}

DNA fragment (260 bp) containing *Caulobacter parS* sites (Toro et al., 2008) was amplified by PCR using primers CCparS_gibMCS5F and CCparS_gibMCS5R, and purified *Caulobacter* genomic DNA as template. The PCR fragment was gel-purified and assembled to an EcoRI-cut pMCS5 backbone (Thanbichler et al., 2007) using a 2x Gibson master mix (NEB). Briefly, 2.5 µL of each DNA fragment at equimolar concentration was added to 5 µL Gibson master mix (NEB), and the mixture was incubated at 50°C for 60 minutes. 5 µL was used to transform chemically-competent *E. coli* DH5α cells. Gibson assembly was possible due to 23 bp sequences shared between the two PCR fragments. These 23 bp regions were incorporated during the primer design to amplify the *parS*-containing sequence. The resulting plasmid was sequence verified by Sanger sequencing (Eurofins, Germany).

pMCS5::parS^{Caulobacter} at +1800kb and pMCS5::parS^{Caulobacter} at +2242kb

For insertion of a 260 bp sequence containing *Caulobacter parS* sites at +1800 kb in the *Caulobacter* genome, primers label1800-NdeI-F and label1800-SacI-R were used to amplify a ~500 bp fragment by PCR, using *Caulobacter* genomic DNA as template. This fragment was 5’ phosphorylated by T4 PNK (NEB) before being blunt-end ligated to a SmaI-cut pUC19 (Fermentas). The resulting construct was sequence verified. The NdeI-SacI-ended fragment was then liberated from the pUC19-based plasmid by NdeI and SacI double digestion before being cloned into the same sites on *pMCS5::parS^{Caulobacter}*. The construction of *pMCS5::parS^{Caulobacter}* for insertion at +2242 kb were carried out essentially as above, except the pairs of primers used were: label2242-NdeI-F and label2242-SacI-R.

Electro-competent *Caulobacter* cells were electroporated with these plasmids to allow for a single integration at the site of interest. The correct integration was verified by PCR using a primer specific to the *parS^{Caulobacter}* site and another primer upstream of the ~500 bp homologous region used to drive integration.

pMT619::parS^{pMT1} at +200 kb, +1000 kb and +1800 kb

For insertion of *parS^{pMT1}* at +200 kb in the *Caulobacter* genome, primers label200-NdeI-F and label200-SacI-R were used to amplify a ~500 bp fragment by PCR, using *Caulobacter* genomic DNA as template. This fragment was 5’ phosphorylated by T4 PNK (NEB) before being blunt-end ligated to a SmaI-cut pUC19 (Fermentas). The resulting construct was sequence verified. The NdeI-SacI-ended fragment was then liberated from the pUC19-based plasmid by NdeI and SacI double digestion before being cloned into the same sites on pMT619::*parS^{pMT1}* (Badrinarayanan et al., 2015). The constructions of pMT619::*parS^{pMT1}* for insertion at +1000 kb, and +1800 kb were carried out essentially as above, except the pairs of primers used were: label1000-NdeI-F and label1000-SacI-R; label1800-NdeI-F and label1800-SacI-R, respectively.

Electro-competent *Caulobacter* cells were electroporated with these plasmids to allow for a single integration at the site of interest. The correct integration was verified by PCR using a primer specific to the *parS*^{pMT1} site and another primer upstream of the ~500 bp homologous region used to drive integration.

pMT632::parS^{P1} at +3842 kb, +3042 kb and +2242 kb

To insert *parS*^{P1} sites at +3842 kb, +3042 kb and +2242 kb in the *Caulobacter* genome, the same procedure as above was used, except that the pairs of primers used were: label3842-NdeI-F and label3842-SacI-R; label3042-NdeI-F and label3042-SacI-R; and label2242-NdeI-F and label2242-SacI-R respectively. Also, the NdeI-SacI-ended fragment was then liberated from the pUC19-based plasmid by NdeI and SacI double digestion before being cloned into the same sites on pMT632::*parS*^{P1} (Badrinarayanan et al., 2015).

Electro-competent *Caulobacter* cells were electroporated with these plasmids to allow for a single integration at the site of interest. The correct integration was verified by PCR using a primer within the *parS*^{P1} site and another upstream of the ~500 bp homologous region used to drive insertion.

pGADT7-parB
The ParB coding sequence was amplified by PCR from *Caulobacter* genomic DNA using primers parB_pGAD424_F and parB_pGAD424_R. The PCR product was purified and assembled to BamHI-EcoRI-cut pGAD424 (Clonetech) using 2x Gibson mastermix. Briefly, 2.5 µL of the PCR product and the BamHI-EcoRI-cut pGAD424 at equimolar concentration was added to 5 µL Gibson master mix (NEB), and the mixture was incubated at 50°C for 60 minutes. 5 µL was used to transform chemically-competent *E. coli* DH5α cells. The resulting plasmid was sequence verified by Sanger sequencing (Eurofins, Germany). The *parB* insert was subsequently dropped out by digesting pGAD424-parB with EcoRI and BamHI, and ligated to EcoRI-BamHI-cut pGADT7 using T4 ligase (NEB). pGADT7 was used for yeast-two hybrid assay since *parB* is driven by a stronger promoter than that of pGAD424.

pGBKT7-parA WT and pGBKT7-parAK20R

The ParA coding sequence was amplified by PCR from *Caulobacter* genomic DNA using primers parA_pGBT_F and parA_pGBT_R. The PCR product was purified and assembled to BamHI-EcoRI-cut pGBT9 (Clonetech) using 2x Gibson master mix. Briefly, 2.5 µL of the PCR product and the BamHI-EcoRI-cut pGBT9 at equimolar concentration was added to 5 µL Gibson master mix (NEB), and the mixture was incubated at 50°C for 60 minutes. 5 µL was used to transform chemically-competent *E. coli* DH5α cells. The resulting plasmid was sequence verified by Sanger sequencing (Eurofins, Germany). The *parA* insert was subsequently dropped out by digesting pGBT9-parA with EcoRI and BamHI, and ligated to EcoRI-BamHI-cut pGBKT7 using T4 ligase (NEB). pGBKT7 was used for yeast two hybrid assay since *parA* is driven by a stronger promoter than that in pGBT9. The *parA (K20R)* was amplified by PCR using the exact same primers as for ParA^{WT} but from a plasmid carrying P_{xyl}-ParA^{K20R}

Generation of Caulobacter strains with knock-in attB/attP attachment site:

For the insertion of *attP/attB* attachment site on the chromosome, pNPTS138::inversion was first introduced to *Caulobacter* CB15N via electroporation. A homology-mediated double crossover was performed as described previously to knock in the *attP/attB* attachment site (Skerker et al., 2005). The insertion was verified using PCR with primers located 50 bp away on the left and on the right hand side of *attP/attB*.

To generate the inversion between insertion point 2 (nt: 4030311) and insertion point 5 (nt: 3611123):

The *attP* site (forward direction) was inserted first at the insertion point 5 via a homolog-mediated double crossover. The resulting strain was made competent and electroporated with pNPTS138::inversion insertion point 2. Again, a homology-mediated double crossover to knock in *attB* (reverse direction) at the insertion point 2 was performed as described previously (Skerker et al., 2005). The insertion at point 2 was verified using PCR with primers located 50 bp away on the left and on the right hand side of *attB*. The resulting strain was again made electroporation competent, transformed with pTORO78 that has ΦC31 integrase under the control of a vanillate-inducible promoter (Toro et al., 2008), and selected for on PYE + chloramphenicol plates. Colonies that formed on PYE + chloramphenicol were restruck on fresh PYE + chloramphenicol twice. PCR amplification was used to verify that an inversion has occurred. A leaky expression of ΦC31 integrase from the P_{van} promoter is sufficient to drive the *in vivo* recombination between *attP* at the insertion point 2 and *attB* at the insertion point 5.

To generate the inversion between insertion point 3 (nt: 3815825) and insertion point 5 (nt: 3611123):

The exact same procedure as above but pNPTS138::inversion insertion point 3 was used instead of pNPTS::inversion insertion point 2

To generate the inversion between insertion point 4 (nt: 3788231) and insertion point 5 (nt: 3611123):

The exact same procedure as above but pNPTS138::inversion insertion point 4 was used instead of pNPTS::inversion insertion point 3.

To generate the inversion between insertion point 1 (nt: 4038385) and insertion point 5 (nt: 3611123):

The exact same procedure as above but pNPTS138::inversion insertion point 1 was used instead of pNPTS::inversion insertion point 4

To generate the inversion between insertion point 2 (nt: 4030311) and insertion point 4 (nt: 3788231):

The *attP* site (forward direction) was inserted first at the insertion point 4 by homology-mediated double crossover. The resulting strain was made competent and electroporated with pNPTS138::inversion insertion point 2. Again, a homology-mediated double crossover to knock in *attB* (reverse direction) at the insertion point 2 was performed as described previously (Skerker et al., 2005).The insertion at point 2 was verified using PCR with primers located 50 bp away on the left and on the right hand side of *attB*. The resulting strains were again made electroporation competent, transformed with pTORO78 that has ΦC31 integrase under the control of a vanillate-inducible promoter (Toro et al., 2008), and selected for on PYE + chloramphenicol plates. Colonies that formed on PYE + chloramphenicol plate were restruck on fresh PYE + chloramphenicol twice. PCR amplification was used to verify that inversion has occurred. A leaky expression of ΦC31 integrase from the P_{van} promoter is sufficient to drive the *in vivo* recombination between *attP* at the insertion point 4 and *attB* at the insertion point 2.

To generate the inversion between insertion point 3 (nt: 3815825) and insertion point 4 (nt: 3788231):

The exact same procedure as above but pNPTS138::inversion insertion point 3 was used instead of pNPTS::inversion insertion point 2

Generation of Δsmc strains with inverted chromosome segment

We employed ΦCr30 generalized phage transduction (Ely, 1991) to move *Δsmc*::kanamycin^{R} or *Δsmc*::tetracycline^{R} alleles from the original deletion strains (ML2117 or ML2118) to the inversion strains.

For complementation of *Δsmc* strains with a plasmid-borne pML477::*flag-smc*, electroporation-competent *Caulobacter Δsmc* was transformed with pML477::*flag-smc* and plated out on PYE + spectinomycin. Colonies that formed on PYE + spectinomycin were restruck out on fresh PYE + spectinomycin plates twice to purify the strain.

Generation of Caulobacter *strains carrying rpoC-flag*

We employed ΦCr30 generalized phage transduction to move *rpoC::rpoC-flag::kanamycin^{R}* allele from the ML2299 strain (Haakonsen et al., 2015) to the wanted background to generate strains TLS1609, TLS1613.

Generation of Caulobacter *strains carrying cfp-parB and an ectopic parS at +1800 kb or +2242 kb*

We employed ΦCr30 generalized phage transduction to move 1800kb::*parS^{Caulobacter}* (marked with tetracycline^{R}) from TLS1619 to MT190 (CB15N *parB::cfp-parB*) to result in strain TLS1620. Similarly, 2242kb::*parS^{Caulobacter}* (marked with tetracycline^{R}) from TLS1621 was transduced into MT190 (CB15N *parB::cfp-parB*) to result in strain TLS1622.

Generation of yeast two hybrid strains

A combination of pGBKT7-based plasmid and pGADT7-based plasmid was co-transformed into the yeast strain Y187 using the Frozen-EZ yeast transformation kit (ZymoResearch) and plated out on SD agar + leucine + tryptophan. The resulting colonies were restruck on SD agar + leucine + tryptophan plate to purify the strain before β-galactosidase assays.

Strains for labelling individual DNA loci:

For strain TLS1602 (CB15N *parS^{pMT1}*::200kb *parS^{P1}*::3842kb *dnaA*::P$_{van}$-*dnaA xyl*::P$_{xyl}$-*mcherry-parB^{P1}-yfp-parB^{pMT1}*): First, *parS^{pMT1}* at +200 kb (marked with spectinomycin^{R}) was transduced into *Caulobacter* strain *dnaA*::P$_{van}$-*dnaA*, then *parS^{P1}* at +3842 kb (marked with chloramphenicol^{R}) was transduced into the resulting strain. Finally, *xyl*::P$_{xyl}$-*mcherry-parB^{P1}-yfp-parB^{pMT1}* (marked with kanamycin^{R}) was transduced in. For strain TLS1605 (CB15N *parS^{pMT1}*::200kb *parS^{P1}*::3842kb *dnaA*::P$_{van}$-*dnaA xyl*::P$_{xyl}$-*mcherry-parB^{P1}-yfp-parB^{pMT1}* *Δsmc*::tetracycline^{R}): ΦCr30 generalized phage transduction was used to move *Δsmc*::tetracycline^{R} allele from the original deletion strains ML2117 to TLS1602.

For strain TLS1603 (CB15N *parS^{pMT1}*::1000kb *parS^{P1}*::3042kb *dnaA*::P$_{van}$-*dnaA* *xyl*::P$_{xyl}$-*mcherry-parB^{P1}-yfp-parB^{pMT1}*): First, *parS^{pMT1}* at +1000kb (marked with spectinomycin^{R}) was transduced into *Caulobacter* strain *dnaA*::P$_{van}$-*dnaA*, then *parS^{P1}* at +3042kb (marked with chloramphenicol^{R}) was transduced into the resulting strain. Finally, *xyl*::P$_{xyl}$-*mcherry-parB^{P1}-yfp-parB^{pMT1}* (marked with kanamycin^{R}) was transduced in. For strain TLS1606 (CB15N *parS^{pMT1}*::1000kb *parS^{P1}*::3042kb *dnaA*::P$_{lac}$-*dnaA* *xyl*::P$_{xyl}$-*mcherry-parB^{P1}-yfp-parB^{pMT1}* *Δsmc*::tetracycline^{R}): ΦCr30 generalized phage transduction was used to move *Δsmc*::tetracycline^{R} allele from the original deletion strains ML2117 to TLS1603.

For strain TLS1604 (CB15N *parS*^{pMT1}::1800kb *parS*^{P1}::2242kb *dnaA*::P$_{van}$-*dnaA* *xyl*::P$_{xyl}$-*mcherry-parB*^{P1}-*yfp-parB*^{pMT1}): First, *parS*^{pMT1} at +1800kb (marked with spectinomycin^{R}) was transduced into *Caulobacter* strain *dnaA*::P$_{van}$-*dnaA*, then *parS*^{P1} at +2242kb (marked with chloramphenicol^{R}) was transduced into the resulting strain. Finally, *xyl*::P$_{xyl}$-*mcherry-parB*^{P1}-*yfp-parB*^{pMT1} (marked with kanamycin^{R}) was transduced in. For strain TLS1607 (CB15N *parS*^{pMT1}::1800kb *parS*^{P1}::2242kb *dnaA*::P$_{lac}$-*dnaA* *xyl*::P$_{xyl}$-*mcherry-parB*^{P1}-*yfp-parB*^{pMT1} *Δsmc*::tetracycline^{R}): ΦCr30 generalized phage transduction was used to move *Δsmc*::tetracycline^{R} allele from the original deletion strains ML2117 to TLS1604.

For strain TLS1669 (CB15N *parS*^{pMT1}::1800kb *parS*^{P1}::1600kb *dnaA*::P$_{van}$-*dnaA* *xyl*::P$_{xyl}$-*mcherry-parB*^{P1}-*yfp-parB*^{pMT1}): First, *parS*^{pMT1} at +1800kb (marked with spectinomycin^{R}) was transduced into *Caulobacter* strain *dnaA*::P$_{van}$-*dnaA*, then *parS*^{P1} at +1600kb (marked with chloramphenicol^{R}) was transduced into the resulting strain. Finally, *xyl*::P$_{xyl}$-*mcherry-parB*^{P1}-*yfp-parB*^{pMT1} (marked with kanamycin^{R}) was transduced in. For strain TLS1670 (CB15N *parS*^{pMT1}::1800kb *parS*^{P1}::1600kb *dnaA*::P$_{lac}$-*dnaA* *xyl*::P$_{xyl}$-*mcherry-parB*^{P1}-*yfp-parB*^{pMT1} *Δsmc*::tetracycline^{R}): ΦCr30 generalized phage transduction was used to move *Δsmc*::tetracycline^{R} allele from the original deletion strains ML2117 to TLS1669.

Chromatin immunoprecipitation with deep sequencing (ChIP-seq) and generation of ChIP-seq profiles

Caulobacter cell cultures (25 mL) were grown in PYE to mid exponential phase (OD ~0.4) and fixed with formaldehyde to a final concentration of 1%. For ChIP-seq of FLAG-tagged SMC, mid-exponential phase cultures were washed twice in 1x PBS buffer before addition of formadehyde and crosslinker Gold (Diagenode) to a final concentration of 1%. Fixed cells were incubated at room temperature for 30 minutes, then quenched with 0.125 M glycine for 15 min at room temperature. Cells were washed three times with 1x PBS (pH 7.4) and resuspended in 1 mL of buffer 1 (20 mM K-HEPES pH 7.9, 50 mM KCl, 10% Glycerol and Roche EDTA-free protease inhibitors). Subsequently, the cell suspension was sonicated on ice using a Soniprep 150 probe-type sonicator (11 cycles, 15s ON, 15s OFF, at setting 8) to shear the chromatin to below 1 kb, and the cell debris was cleared by centrifugation (20 minutes at 13,000 rpm at 4°C).

The supernatant was then transferred to a new 2 mL tube and the buffer conditions were adjusted to 10 mM Tris-HCl pH 8, 150 mM NaCl and 0.1% NP-40. Fifty microliters of the supernatant were transferred to a separate tube for control (the INPUT fraction) and stored at -20°C. In the meantime, antibodies-coupled beads were washed off storage buffers before being added to the above supernatant. We employed α-GFP antibodies coupled to sepharose beads (Abcam, UK) for ChIP-seq of CFP-ParB, α-FLAG antibodies coupled to agarose beads (Sigma, UK) for ChIP-seq of RpoC-FLAG and FLAG-SMC. Briefly, 25 μL of α-GFP beads or 100 μL α-FLAG beads was washed off storage buffer by repeated centrifugation and resuspension in IPP150 buffer (10 mM Tris-HCl pH 8, 150 mM NaCl and 0.1% NP-40). Beads were then introduced to the cleared supernatant and incubated with gentle shaking at 4°C overnight. In the next day, beads were then washed five times at 4°C for 2 min each with 1 mL of IPP150 buffer, then twice at 4°C for 2 min each in 1x TE buffer (10 mM Tris-HCl pH 8 and 1 mM EDTA). Protein-DNA complexes were then eluted twice from the beads by incubating the beads first with 150 μL of the elution buffer (50 mM Tris-HCl pH 8, 10 mM EDTA and 1% SDS) at 65°C for 15 min, then with 100 μL of 1X TE buffer + 1% SDS for another 15 min at 65°C. The supernatant (the ChIP fraction) was then separated from the beads and further incubated at 65°C overnight to completely reverse crosslink. The INPUT fraction was also de-crosslinked by incubation with 200 μL of 1X TE buffer + 1% SDS at 65°C overnight. DNA from the ChIP and INPUT fraction were then purified using the PCR

purification kit (Qiagen) according to the manufacturer's instruction, then eluted out in 40 µL of a 10-fold-diluted EB buffer (Qiagen). The purified DNA was then used directly for qPCR or being made in to library suitable for Illumina sequencing using the NEXT Ultra library preparation kit (NEB). ChIP libraries were sequenced on the Illumina Hiseq 2500 at the Tufts University Genomics facility. For the list of ChIP-seq datasets in this study, see Supplementary Table S3.

For analysis of ChIP-seq data, Hiseq 2500 Illumina short reads (50 bp) were mapped back to the *Caulobacter* NA1000 reference genome (NCBI Reference Sequence: NC-011916.1) using Bowtie 1 (Langmead et al., 2009) using the following command:

*bowtie -m 1 -n 1 --best --strata -p 4 --chunkmbs 512 NA1000-2014-bowtie --sam *.fastq > output.sam*

For *Caulobacter* strains with an inverted DNA segment, a reconstructed fasta file with the correct orientation for the inverted segment was used as reference genome for Bowtie instead. Subsequently, sequencing reads were allocated to their corresponding 1-kb bins along the chromosome, and were normalized for the total number of reads to give the RPKPM value (number of reads per kb per million mapped reads). Finally, the profiles of SMC enrichment were plotted with the x-axis representing genomic positions and the y-axis is RPKPM from the ChIP fraction of FLAG-tagged SMC divided by RPKPM from the ChIP fraction of un-tagged SMC, using a custom R script.

Chromosome conformation capture with deep sequencing (Hi-C)
Hi-C experiments were performed exactly as described previously (Le et al., 2013). The restriction enzyme BglII were used for all Hi-C experiments in this study. Cells at OD_{600} of 0.2 were fixed with 1% formadehyde for 30 minutes before quenching by 0.125 M glycine. Fixed cells were washed twice in 1x M2 buffer before being resuspended in 1x TE buffer (10 mM Tris-HCl pH8 and 1 mM EDTA) and subjected to the Hi-C procedure (Le et al., 2013).

For Hi-C of ParB-depleted MT148 (Thanbichler and Shapiro, 2006), cells at mid-exponential phase were washed off xylose by repeated centrifugation and resuspension in fresh PYE before releasing to PYE+0.2% glucose. The culture was left shaking at 30°C for five more hours. The culture was adjusted with fresh PYE to OD_{600} of 0.2 before formadehyde was added to 1% final concentration to fix cells for Hi-C assay. Glycine (0.125M final concentration) was then added to quench the fixation. Fixed cells were washed twice in 1x M2 buffer before being resuspended in 1x TE buffer and subjected to the Hi-C procedure (Le et al., 2013).

Generation of Hi-C contacts maps and inter-arm Hi-C intensity plot
Each end of paired-end sequencing reads was mapped independently to the genome of *Caulobacter crescentus* NA1000 using Bowtie 2.1.0 and an algorithm used that iteratively increases truncation length to maximize the yield of valid Hi-C interactions (Imakaev *et al*, 2012). The *Caulobacter* NA1000 genome was then divided into restriction fragments (700 BglII fragments). Each read of a read pair was sorted into its corresponding restriction fragment. Read pairs were classified as valid Hi-C products, non-ligation, or self-ligation products (Imakaev et al., 2012). Only valid Hi-C products that are uniquely aligned to the *Caulobacter* genome were employed to generate Hi-C contact maps (Supplementary Table S3). To create interaction matrices, the *Caulobacter* genome was first divided into 405 10-kb bins. We then assigned valid Hi-C products to the 10-kb bins and normalised using an iterative correction procedure as described previously (Imakaev et al., 2012; Le et al., 2013). Subsequent analysis and visualization of the contact maps was done using R scripts. For *Caulobacter* strains with an inverted DNA segment, a reconstructed fasta file with the correct

orientation for the inverted segment was used as reference genome for Bowtie instead. All Hi-C contact maps in this manuscript report the log_e-scale of Hi-C interaction between 10-kb bins of DNA loci. Note that, in contrast to Hi-C maps reported previously (Le et al., 2013), here we use the logarithm of interaction frequencies to facilitate visualization of these weaker inter-arm interactions. The axes of Hi-C maps are oriented such as the origin of replication (*ori*: 0 kb or +4043 kb) is at the center of the x- and y-axis, and the left and the right chromosomal arm are on either side.

To determine the extent of inter-chromosomal-arm interactions, inter-arm Hi-C intensity was plotted against genomic positions (see also Fig. 1D). Inter-arm interaction was defined as Hi-C interactions between a 10-kb DNA bin and another bin equidistant from *ori* but on the opposite chromosomal arm. Essentially, this is the Hi-C interactions along the secondary diagonal spanning from the upper left corner to the lower right corner of each Hi-C contact map.

Calculate the rate of arm progression following ParB replenishment

First, the difference Hi-C maps between various time points (5, 10, 15, 20, 25 minutes after adding back xylose) and time point 0 were calculated by subtracting Hi-C maps at t5, t10, t15 or t25 from the t0 map. The procedure revealed the difference in DNA-DNA interactions between difference time points (Fig. S1E). The locator() function in R was then used to determine the genomic position of the leading edge of aligned DNA (black dashed lines in Fig. S1E), and the distance from the edge to *parS* site was calculated for each arm. We plotted the distance from *parS* to the leading edge against time points after adding xylose. We did not observe noticeable difference in arm progression between t0 and t5, therefore we used data from t10, t15, t20 and t25 to estimate the rate of arm progression. To estimate the rate, a linear regression line was fitted to these data. The rates of arm progression and the standard errors were calculated from the slope and its standard error of the best-fit lines.

Analyses of gene expression, RNA polymerase density and transcription orientation

RNA-seq data (Le and Laub, 2016) from CB15N *Caulobacter* growing at exponential phase in rich medium at 30°C were used to analyze the expression of genes that are oriented in the *ori-ter* or in the opposite direction i.e. *ter* to *ori*. For genes residing on the right arm of the chromosome (0 kb to +2000 kb), *ori-ter* oriented genes are on the plus strand (denoted with a + on a standard general feature fomat (gff) file) and *ter-ori* genes are on the minus strand (annotated as - as in the gff file). The opposite is true for genes on the left arm of the chromosome (+2000 kb to +4043 kb). The general feature format (gff) file for *Caulobacter* NA1000 was downloaded from NCBI (ftp://ftp.ncbi.nlm.nih.gov/genomes/Bacteria/*Caulobacter*-crescentus-NA1000-uid59307/), and used as a basis to separate genes to the plus or minus strand. Absolute expression value for each gene was calculated as the number of reads per million of mapped read (Le and Laub, 2016).

When using the enrichment of RpoC-FLAG ChIP-seq, only reads within the coding sequence i.e. excluding the promoter region, are used. This reflects the RNA polymerases that are elongating within the coding sequence instead of at the initiation phase at the promoter region. Pulled-down DNA from initiating RNA polymerases at promoter regions were discarded *in silico*. Again, genes are separated to the *ori-ter* or *ter-ori* direction as for the RNA-seq data above. Absolute RpoC enrichment value for each gene was calculated as the number of reads per kb per million of mapped read (RPKPM) * gene length (in kb). High expression genes are defined as genes with RPKPM*gene length values greater than 1000 (See Fig. 3A-B, Fig. 4A, and Fig. S6A and C).

Immunoblot analysis

For Western blot analysis, *Caulobacter* cells were pelleted and resuspended directly in 1x SDS sample buffer, then heated to 95°C for 5 min before loading. Total protein was run on 10% Tris-HCl gels (Bio-Rad) at 150 V for separation. Resolved proteins were transferred to polyvinylidene fluoride membranes using the Trans-Blot Turbo Transfer System (Biorad) and probed with 1:5,000 dilution of primary α-FLAG antibodies (Sigma-Aldrich), or 1:5,000 dilution of α-ParB antibody (custom antibody from CRB Cambridge, UK) and subsequently by a secondary horseradish peroxidase–conjugated antibody (1:10,000). Blots were imaged using an Amersham Imager 600 (GE Healthcare), and quantified using Image Studio Lite (LI-COR Biosciences).

Orthogonal ParB/*parS* system to label DNA loci and microscopy analysis

C. crescentus strains with *parS*^{pMT1} or *parS*^{P1} inserted at various locations on the chromosome was created as described above. These strains also harboured a P_{xyl}-m*cherry-parB*^{P1}-*yfp-parB*^{pMT1} cassette at the *xyl* locus. Strains were grown to OD_{600}=0.4 in the presence of appropriate antibiotics, vanillate, and glucose before the cells were collected by centrifugation and washed of residual vanillate, antibiotics and glucose twice with fresh PYE. Cells were then resuspended in PYE plus xylose (0.3% final concentration) (vanillate omitted) to deplete DnaA for 90 min before synchronization, thereby inducing cell elongation. Cells were imaged at 0 hr, 1 hr, 2 hr and 3 hr post synchronization. Phase contrast (150 ms exposure) and fluorescence images (2000 ms exposure) were collected. MicrobeTracker (http://microtracker.org) was used to detect cell outlines and SpotFinderZ to detect fluorescent foci positions (Sliusarenko et al., 2011). Only cells with a single Mcherry-ParB^{P1} and YFP-ParB^{pMT1} focus were used for construction of boxplots. Cells were then sorted into bins (<2.5 µm, 2.5-3.5 µm, 3.5-4.5 µm and 4.5-5.5 µm) according to their length. The number of cells used for construction of boxplots in Fig. 3 was as follows:

Labeled loci		< 2.5 µm	2.5-3.5 µm	3.5-4.5 µm	4.5-5.5 µm
200 kb-3842 kb	*smc*$^{+}$	232	915	593	351
	smc^{-}	218	1400	878	354
1000 kb-3042 kb	*smc*$^{+}$	177	922	697	396
	smc^{-}	179	1328	1246	746
1800 kb-2242 kb	*smc*$^{+}$	109	1059	855	414
	smc^{-}	118	1199	990	428
1600 kb-1800 kb	*smc*$^{+}$	85	868	903	495
	smc^{-}	106	1085	1112	616

Boxplots show the distribution of inter-foci distances for cells of different sizes were plotted using R programme. A one-tailed student's t-test was employed to test if inter-foci distances in *Δsmc* cells is greater than in wild-type cells.

Yeast-two hybrid assay

Yeast-two hybrid assays were performed exactly as described in the Clonetech manual. ParB was expressed as a fusion to the activation domain of Gal4 (AD), and ParA^{WT} or ParA^{K20R} as a fusion to the DNA-binding domain of Gal4 (BD). Four biological replicates were performed to obtain the mean and standard deviation of β-galactosidase activity.

TABLE S1, Related to the Experimental Procedures

**Strains**	**Description**	**Source**
***Caulobacter crescentus* strains**		
CB15N	Wild-type synchronizable *Caulobacter crescentus*	Lab collection
ML2000	CB15N *hfa::lacI dnaA::*P_{lac}-*dnaA*	Badrinarayanan et al., 2015
ML2413	ML2000 *xyl::*P_{xyl}-*parA*^{K20R}-*yfp* (gentamycin^{R})	Badrinarayanan et al., 2015
ML2414	ML2000 *xyl::*P_{xyl}-*parA*^{WT}-*yfp* (gentamycin^{R})	Badrinarayanan et al., 2015
ML2299	CB15N *rpoC::rpoC-flag*::kanamycin^{R}	Haakonsen et al., 2015
ML2118	CB15N *Δsmc*::kanamycin^{R}	Le et al., 2013
ML2117	CB15N *Δsmc*::tetracycline^{R}	Le et al., 2013
MT148	CB15N *parB::*P_{xyl}-*parB*	gift from Martin Thanbichler, Thanbichler and Shapiro, 2006
MT190	CB15N *parB::cfp-parB*	gift from Martin Thanbichler, Thanbichler and Shapiro, 2006
TLS1599	CB15N *Δsmc*::kanamycin^{R} pML477::*flag-smc*	
TLS1600	CB15N *ΔscpA*::kanamycin^{R}	This study
TLS1601	CB15N *ΔscpB*::markerless	This study
TLS1602	CB15N *parS*^{pMT1}::200 kb *parS*^{P1}::3842 kb *dnaA::*P_{van}-*dnaA xyl::*P_{xyl}-*mcherry-parB*^{P1}-*yfp-parB*^{pMT1}	This study
TLS1603	CB15N *parS*^{pMT1}::1000 kb *parS*^{P1}::3042 kb *dnaA::*P_{van}-*dnaA xyl::*P_{xyl}-*mcherry-parB*^{P1}-*yfp-parB*^{pMT1}	This study
TLS1604	CB15N *parS*^{pMT1}::1800 kb *parS*^{P1}::2242 kb *dnaA::*P_{van}-*dnaA xyl::*P_{xyl}-*mcherry-parB*^{P1}-*yfp-parB*^{pMT1}	This study
TLS1605	CB15N *parS*^{pMT1}::200 kb *parS*^{P1}::3842 kb *dnaA::*P_{van}-*dnaA xyl::*P_{xyl}-*mcherry-parB*^{P1}-*yfp-parB*^{pMT1}*Δsmc*::tetracycline^{R}	This study
TLS1606	CB15N *parS*^{pMT1}::1000 kb *parS*^{P1}::3042 kb *dnaA::*P_{van}-*dnaA xyl::*P_{xyl}-*mcherry-parB*^{P1}-*yfp-parB*^{pMT1}*Δsmc*::tetracycline^{R}	This study
TLS1607	CB15N *parS*^{pMT1}::1800 kb *parS*^{P1}::2242 kb *dnaA::*P_{van}-*dnaA xyl::*P_{xyl}-*mcherry-parB*^{P1}-*yfp-parB*^{pMT1}*Δsmc*::tetracycline^{R}	This study
TLS1669	CB15N *parS*^{pMT1}::1800 kb *parS*^{P1}::1600 kb *dnaA::*P_{van}-*dnaA xyl::*P_{xyl}-*mcherry-parB*^{P1}-*yfp-parB*^{pMT1}	This study

TLS1670	CB15N *parS*^{pMT1}::1800 kb *parS*^{P1}::1600 kb *dnaA::*P$_{van}$-*dnaA xyl::*P$_{xyl}$-*mcherry-parB*^{P1}-*yfp-parB*^{pMT1} *Δsmc*::tetracycline^{R}	This study
TLS1608	Flip 1-5: inversion between end point 1 (nt 4038432) and end point 5 (nt 3611123)	This study
TLS1609	Flip 1-5 *rpoC::rpoC-flag*::kanamycin^{R}	This study
TLS1610	Flip 1-5 *Δsmc*::kanamycin^{R}	This study
TLS1611	Flip 1-5 *Δsmc*::kanamycin^{R} pML477::*flag-smc*	This study
TLS1612	Flip 2-5: inversion between end point 2 (nt 4030311) and end point 5 (nt 3611123)	This study
TLS1613	Flip 2-5 *rpoC::rpoC-flag*::kanamycin^{R}	This study
TLS1614	Flip 2-5 *Δsmc*::kanamycin^{R}	This study
TLS1615	Flip 2-5 *Δsmc*::kanamycin^{R} pML477::*flag-smc*	This study
TLS1616	Flip 4-5: CB15N, inversion between end point 4 (nt 3788231) and end point 5 (nt 3611123)	This study
TLS1617	Flip 2-4 : CB15N, inversion between endpoint 2 (nt 4030311) and endpoint 4 (nt 3788231)	This study
TLS1618	Flip 3-4: CB15N, inversion between endpoint 3 (nt 3815825) and endpoint 4 (nt 3788231)	This study
TLS1619	CB15N 1800kb::*parS*^{Caulobacter} (260bp)	This study
TLS1620	CB15N *parB::cfp-parB* 1800kb::*parS*^{Caulobacter} (260bp)	This study
TLS1621	CB15N 2242kb::*parS*^{Caulobacter} (260bp)	This study
TLS1622	CB15N *parB::cfp-parB* 2242kb::*parS*^{Caulobacter} (260bp)	This study
TLS1623	ML2000 *Δsmc*::kanamycin^{R}	This study
**Yeast strains**		
	Y187	Clonetech
TLS1624	Y187 pGADT7-*parB* + pGBKT7-*parA*^{WT}	This study
TLS1625	Y187 pGADT7-*parB* + pGBKT7-*parA*^{K20R}	This study
TLS1626	Y187 pGADT7-*parB* + pGBKT7-empty	This study

TABLE S2, Related to the Experimental Procedures

**Plasmids**	**Description**	
pENTR-D-TOPO	ENTRY vector for gateway cloning, kanamycin^{R}	Invitrogen
pML477	Gateway-cloning destination vector for fusion of protein interest to an N-terminally FLAG tag, xylose-inducible promoter, medium-copy number plasmid, spectinomycin^{R}	Laub lab strain collection
pNPTS138	integrative vector for *Caulobacter* gene knockout/knock-in, kanamycin^{R}	Laub lab strain collection
pMCS2	integrative vector for *Caulobacter* gene knockout, kanamycin^{R}	Thanbichler et al., 2007
pNPTS138::*ΔscpB*	for the deletion of *scpB*	This study

pMCS2::*ΔscpA*	for the deletion of *scpA*	This study
pGADT7	vector for yeast-two hybrid assay, carbenicilin^R	Clonetech
pGBKT7	vector for yeast-two hybrid assay, carbenicilin^R	Clonetech
pGADT7::*parB*	expressing AD-ParB fusion protein, carbenicilin^R	This study
pBGKT7::*parA*^{K20R}	expressing DB-ParA fusion protein, carbenicilin^R	This study
pGBKT7::*parA*^{WT}	expressing DB-ParA^{K20R} fusion protein, carbenicilin^R	This study
pMT619:: *parS*^{pMT1} at +200kb	label +200 kb with *parS*^{pMT1} site, spectinomycin^R	This study
pMT632:: *parS*^{P1} at +3842kb	label +3842 kb with *parS*^{P1} site, chloramphenicol^R	This study
pMT619:: *parS*^{pMT1} at +1000kb	label +1000 kb with *parS*^{pMT1} site, spectinomycin^R	This study
pMT632: *parS*^{P1} at +3042kb	label +3042 kb with *parS*^{P1} site, chloramphenicol^R	This study
pMT619:: *parS*^{pMT1} at +1800kb	label +1800 kb with *parS*^{pMT1} site, spectinomycin^R	This study
pMT632:: *parS*^{P1} at +2242kb	label +2242 kb with *parS*^{P1} site, chloramphenicol^R	This study
pMCS5-*parS*^{Caulobacter}	plasmid for insertion of a second *Caulobacter parS* site (260 bp) at an ectopic location on the chromosome, tetracycline^R	This study
pMCS5:: *parS*^{Caulobacter} at +1800 kb	label +1800 kb with *Caulobacter parS* site (260 bp), tetracycline^R	This study
pMCS5:: *parS*^{Caulobacter} at +2242 kb	label +2242 kb with *Caulobacter parS* site (260 bp), tetracycline^R	This study
pNPTS138::inversion insertion point 1	for an insertion of *attP* (forward direction) at nucleotide (nt) position 4038385	This study
pNPTS138::inversion insertion point 2	for an insertion of *attB* (reverse direction) at nucleotide (nt) position 4030311	This study
pNPTS138::inversion insertion point 3	for an insertion of *attB* (reverse direction) at nucleotide (nt) position 3815825	This study
pNPTS138::inversion insertion point 4	for an insertion of *attP* (forward direction) at nucleotide (nt) position 3788231	This study
pNPTS138::inversion insertion point 5	for an insertion of *attB* (reverse direction) at nucleotide (nt) position 3611123	This study
pTORO78	pMT425-ΦC31, expressing *Streptomyces* ΦC31 integrase from a vanilate-inducible promoter, low-copy number plasmid, chloramphenicol^R	Gift from Lucy Shapiro, Toro et al 2008
pENTR::*smc*	entry vector harboring the coding sequence of SMC, kanamycin^R	This study
pML477:*flag-smc*	expressing flag-smc, spectinomycin^R	This study

**Primers**	**Sequence**	
**For the construction of pMCS2::*ΔscpA***		
scpA_500N_F	gctttcgcgagacgtccaattgcacacgggctttcagcccacctttgac	This study
scpA_500N_R	aactagtggatcccccgggctgcagcatcaggccatagaggtcgccctc	This study
**For the construction of pNPTS138::*ΔscpB***		
scpBfrag1_F	ccaagcttctctgcaggatatctggcctacctgaaatcgcgcctgctgctg	This study
scpBfrag1_R	gaaatcctgcgcgcagcgctcgacgaagagaggatcc	This study
scpBfrag2_F	gtcgagcgctgcgcgcaggatttcctgggcgagcccgaaaaag	This study
scpBfrag2_F	gagacgcgtcacggccgaagctagcgcggcggcgatgatgagaagttctgtg	This study
**For the construction of pENTR::*smc***		
smc-ENTR-D-TOPO-F	CACCgtgcagttccagcgcctccgcctg	This study
smc-ENTR-D-TOPO-R	ttaagccgccaccagcttctccgccg	This study
**For the construction of pMCS5:: *parS*^{Caulobacter}**		
CCparS_gibMCS5F	accttaagatctcgagctccggaggcccgggccctggagcgcatctccg	This study
CCparS_gibMCS5R	ctagcaccggtacgcgtaacgttcgcaagacgctcgcctcaatgcgaac	This study
**For the construction of pGADT7-*parB***		
parB-pGAD424_F	accaaacccaaaaaaagagatcgaattcggtggtggttccatgtccgaagggcgtcgtggtc	This study
parB-pGAD424_R	tcatagatctctgcaggtcgacggatccctcagatcccgcgcgtcagtcggttg	This study
**For the construction of pGBKT7-*parA*^{WT} and pGBKT7-*parA*^{K20R}**		
parA-pGBT_F	aaagacagttgactgtatcgccggaattcggtggtggttccgtgtccgctaatcctctccgcg	This study

parA-pGBT_R	attagcttggctgcaggtcgacggatcccttaggcggccttggcctggc gatcg	This study
**For the construction of pMT619::*parS*^{pMT1}**		
label200-NdeI-F	CATATGatcgaaaagaccttcaagctg	This study
label200-SacI-R	GAGCTCtcacgcctttcccatatagatgaac	This study
label1000-NdeI-F	CATATGttgggcttaggtgtggaccacg	This study
label1000-SacI-R	GAGCTCctaccgccgcttcaacttcgccag	This study
label1800-NdeI-F	CATATGctgccgatgacggaggcggcctac	This study
label1800-SacI-R	GAGCTCtcatggacgggcgctcccgtgac	This study
**For the construction of pMT632::*parS*^{P1}**		
label2242-NdeI-F	CATATGgtgaacggacaagtgggggaacac	This study
label2242-SacI-R	GAGCTCtcaggcggggaacatcctcgccag	This study
label3042-NdeI-F	CATATGatcagacccaccctgaccgcc	This study
label3042-SacI-R	GAGCTCctactcctccaccccatgggcgtag	This study
label3842-NdeI-F	CATATGttgcgtaacggcgaactgcggcgc	This study
label3842-SacI-R	GAGCTCttactgacgcgccttggccacc	This study

Uppercase letters denote restriction enzyme recognition site or sequence required for Gateway TOPO cloning

TABLE S3, Related to the Experimental Procedures

**Hi-C datasets**	**Restriction enzymes**	**GEO**
CB15N in PYE, synchronized	BglII	GSE45966
ML2118 in PYE, synchronized	BglII	GSE45966
TLS1612, synchronized, rep 1	BglII	This study GSE97330
TLS1612, synchronized, rep 2	BglII	This study GSE97330
TLS1614, synchronized	BglII	This study GSE97330
TLS1612 + 25 µg/ml rifampicin for 30min, synchronized	BglII	This study GSE97330
TLS1608, synchronized, rep 1	BglII	This study GSE97330
TLS1608, synchronized, rep 2	BglII	This study GSE97330
TLS1608, synchronized, rep 3	BglII	This study GSE97330
TLS1610, synchronized	BglII	This study

		GSE97330
TLS1616, synchronized	BglII	This study GSE97330
TLS1616 + 25 µg/ml rifampicin for 30min, synchronized	BglII	This study GSE97330
TLS1617, synchronized	BglII	This study GSE97330
TLS1617 + 25 µg/ml rifampicin for 30min, synchronized	BglII	This study GSE97330
TLS1618, synchronized	BglII	This study GSE97330
TLS1618 + 25 µg/ml rifampicin for 30min, synchronized	BglII	This study GSE97330
ML2413 + xylose (0.3%) for 1 hr, rep1	BglII	This study GSE97330
ML2413 + xylose (0.3%) for 1 hr, rep2	BglII	This study GSE97330
ML2414 + xylose (0.3%) for 1 hr, rep1	BglII	This study GSE97330
ML2414 + xylose (0.3%) for 1 hr, rep2	BglII	This study GSE97330
MT148 + glucose (0.2%) for 5 hrs	BglII	This study GSE97330
MT148 ParB depletion, 5hr after xylose withdrawal, rep 1	BglII	This study GSE97330
MT148 ParB depletion, 5hr after xylose withdrawal, rep 2	BglII	This study GSE97330
MT148 ParB replenishment, 5 min after adding xylose (0.3%)	BglII	This study GSE97330
MT148 ParB replenishment, 10 min after adding xylose (0.3%)	BglII	This study GSE97330
MT148 ParB replenishment, 15 min after adding xylose (0.3%)	BglII	This study GSE97330
MT148 ParB replenishment, 20 min after adding xylose (0.3%)	BglII	This study GSE97330
MT148 ParB replenishment, 25 min after adding xylose (0.3%)	BglII	This study GSE97330
TLS1599, 0 min after adding xylose (0.3%)	BglII	This study GSE97330
TLS1599, 15 min after adding xylose (0.3%)	BglII	This study GSE97330
TLS1599, 30 min after adding xylose (0.3%)	BglII	This study GSE97330
TLS1599, 60 min after adding xylose (0.3%)	BglII	This study GSE97330
TLS1600, synchronized, rep 1	BglII	This study GSE97330
TLS1600, synchronized, rep 2	BglII	This study GSE97330

TLS1601, synchronized	BglII	This study GSE97330
TLS1619, synchronized	BglII	This study GSE97330
TLS1619, mixed population	BglII	This study GSE97330
TLS1621, synchronized	BglII	This study GSE97330
CB15N + 25 µg/ml rifampicin for 30min, synchronized, rep 1	BglII	This study GSE97330
CB15N + 25 µg/ml rifampicin for 30min, synchronized, rep 2	BglII	This study GSE97330
CB15N + 25 µg/ml rifampicin for 30min, synchronized, rep 3	BglII	This study GSE97330
CB15N, synchronized, rep 1	BglII	This study GSE97330
CB15N, synchronized, rep 2	BglII	This study GSE97330
CB15N, synchronized, rep 3	BglII	This study GSE97330
ML2000, 90 minutes after IPTG withdrawal, synchronized	BglII	GSE74364
ML2000, 150 minutes after IPTG withdrawal, synchronized	BglII	GSE74364
ML2000, 210 minutes after IPTG withdrawal, synchronized	BglII	GSE74364
ML2000, 270 minutes after IPTG withdrawal, synchronized	BglII	GSE74364
TLS1623, 90 minutes after IPTG withdrawal, synchronized	BglII	GSE74364
TLS1623, 210 minutes after IPTG withdrawal, synchronized	BglII	GSE74364

**ChIP-seq datasets**	**GEO**
CB15N, fixation with 1% formadehyde and crosslinker Gold (Diagenode), α-FLAG antibody (Sigma), ChIP fraction	This study GSE97330
TLS1599, fixation with 1% formadehyde and crosslinker Gold (Diagenode), α -FLAG antibody (Sigma), ChIP fraction	This study GSE97330
TLS1608, fixation with 1% formadehyde and crosslinker Gold (Diagenode), α -FLAG antibody (Sigma), ChIP fraction	This study GSE97330
TLS1611, fixation with 1% formadehyde and crosslinker Gold (Diagenode), α -FLAG antibody (Sigma), ChIP fraction	This study GSE97330
TLS1612, fixation with 1% formadehyde and crosslinker Gold (Diagenode), α-FLAG antibody (Sigma), ChIP fraction	This study GSE97330
TLS1615, fixation with 1% formadehyde and	This study

crosslinker Gold (Diagenode), α-FLAG antibody (Sigma), ChIP fraction	GSE97330
ML2299, fixation with 1% formadehyde, α-FLAG antibody (Sigma), ChIP fraction	This study GSE97330
TLS1609, fixation with 1% formadehyde, α-FLAG antibody (Sigma), ChIP fraction	This study GSE97330
TLS1613, fixation with 1% formadehyde, α-FLAG antibody (Sigma), ChIP fraction	This study GSE97330
MT190, fixation with 1% formadehyde, α-GFP antibody (Abcam), ChIP fraction	This study GSE97330
TLS1620, fixation with 1% formadehyde, α-GFP antibody (Abcam), ChIP fraction	This study GSE97330
TLS1622, fixation with 1% formadehyde, α-GFP antibody (Abcam), ChIP fraction	This study GSE97330
CB15N, fixation with 1% formadehyde, α-FLAG antibody (Sigma), ChIP fraction	This study GSE97330

SUPPLEMENTARY REFERENCES

Badrinarayanan, A., Le, T.B.K., and Laub, M.T. (2015). Rapid pairing and resegregation of distant homologous loci enables double-strand break repair in bacteria. J. Cell Biol. *210*, 385–400.

Ely, B. (1991). Genetics of Caulobacter crescentus. Methods Enzym. *204*, 372–384.

Haakonsen, D.L., Yuan, A.H., and Laub, M.T. (2015). The bacterial cell cycle regulator GcrA is a σ70 cofactor that drives gene expression from a subset of methylated promoters. Genes Dev. *29*, 2272–2286.

Imakaev, M., Fudenberg, G., McCord, R.P., Naumova, N., Goloborodko, A., Lajoie, B.R., Dekker, J., and Mirny, L.A. (2012). Iterative correction of Hi-C data reveals hallmarks of chromosome organization. Nat Methods *9*, 999–1003.

Kahng, L.S., and Shapiro, L. (2003). Polar localization of replicon origins in the multipartite genomes of Agrobacterium tumefaciens and Sinorhizobium meliloti. J.Bacteriol. *185*, 3384–3391.

Langmead, B., Trapnell, C., Pop, M., and Salzberg, S.L. (2009). Ultrafast and memory-efficient alignment of short DNA sequences to the human genome. Genome Biol *10*, R25.

Le, T.B., and Laub, M.T. (2016). Transcription rate and transcript length drive formation of chromosomal interaction domain boundaries. EMBO J. *35*, 1582–1595.

Le, T.B., Imakaev, M.V., Mirny, L.A., and Laub, M.T. (2013). High-resolution mapping of the spatial organization of a bacterial chromosome. Science *342*, 731–734.

Skerker, J.M., Prasol, M.S., Perchuk, B.S., Biondi, E.G., and Laub, M.T. (2005). Two-component signal transduction pathways regulating growth and cell cycle progression in a bacterium: a system-level analysis. PLoS Biol *3*, e334.

Sliusarenko, O., Heinritz, J., Emonet, T., and Jacobs-Wagner, C. (2011). High-throughput, subpixel precision analysis of bacterial morphogenesis and intracellular spatio-temporal dynamics. Mol Microbiol *80*, 612–627.

Thanbichler, M., and Shapiro, L. (2006). MipZ, a Spatial Regulator Coordinating Chromosome Segregation with Cell Division in Caulobacter. Cell *126*, 147–162.

Toro, E., Hong, S.-H., McAdams, H.H., and Shapiro, L. (2008). Caulobacter requires a dedicated mechanism to initiate chromosome segregation. Proc. Natl. Acad. Sci. *105*, 15435–15440.
