# Supplementary material for: SMC Progressively Aligns Chromosomal Arms in Caulobacter crescentus but Is Antagonized by Convergent Transcription
Source: Cell Rep. 2017 Aug 29;20(9):2057–71. doi: 10.1016/j.celrep.2017.08.026 (PMC5583512; doi:10.1016/j.celrep.2017.08.026)
Supplement: Document S2. Article plus Supplemental Information [file mmc2.pdf]

Cell Reports

Article

SMC Progressively Aligns Chromosomal Arms in *Caulobacter crescentus* but Is Antagonized by Convergent Transcription

Graphical Abstract

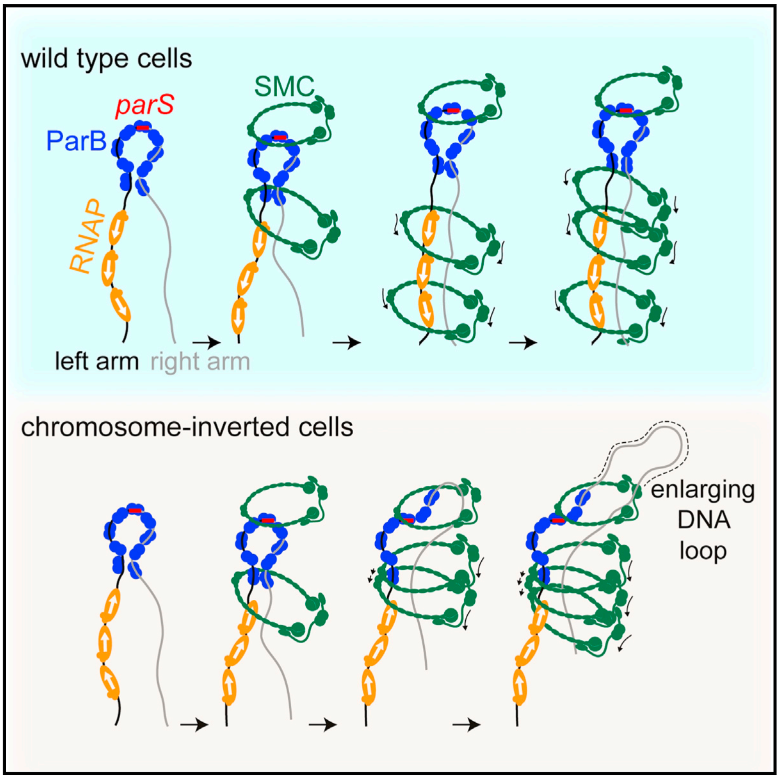

Highlights

- *Caulobacter* SMC aligns the two chromosomal arms progressively from *ori* to *ter*
- SMC is loaded at *parS*, and ParB is essential for SMC-mediated arm alignment
- SMC likely functions as a tether to cohese *parS*-proximal DNA together
- Head-on transcription interferes with SMC translocation from *parS*

Authors

Ngat T. Tran, Michael T. Laub, Tung B.K. Le

Correspondence

tung.le@jic.ac.uk

In Brief

Tran et al. investigate the mechanism and function of SMC in the global organization of the *Caulobacter* chromosome. The findings suggest that SMC functions as a tether to actively cohese the chromosomal arms together and show that head-on transcription profoundly interferes with SMC translocation from the centromeric *parS* site.

Accession Numbers

GSE97330

Tran et al., 2017, Cell Reports *20*, 2057–2071
August 29, 2017 © 2017 The Author(s).
http://dx.doi.org/10.1016/j.celrep.2017.08.026

CellPress

Cell Reports
Article

OPEN ACCESS
CellPress

SMC Progressively Aligns Chromosomal Arms in *Caulobacter crescentus* but Is Antagonized by Convergent Transcription

Ngat T. Tran,[1] Michael T. Laub,[2,3] and Tung B.K. Le[1,4,*]
[1]Department of Molecular Microbiology, John Innes Centre, Norwich NR4 7UH, UK
[2]Department of Biology
[3]Howard Hughes Medical Institute
Massachusetts Institute of Technology, Cambridge, MA 02139, USA
[4]Lead Contact
*Correspondence: tung.le@jic.ac.uk
http://dx.doi.org/10.1016/j.celrep.2017.08.026

SUMMARY

The structural maintenance of chromosomes (SMC) complex plays an important role in chromosome organization and segregation in most living organisms. In *Caulobacter crescentus*, SMC is required to align the left and the right arms of the chromosome that run in parallel down the long axis of the cell. However, the mechanism of SMC-mediated alignment of chromosomal arms remains elusive. Here, using genome-wide methods and microscopy of single cells, we show that *Caulobacter* SMC is recruited to the centromeric *parS* site and that SMC-mediated arm alignment depends on the chromosome-partitioning protein ParB. We provide evidence that SMC likely tethers the *parS*-proximal regions of the chromosomal arms together, promoting arm alignment. Furthermore, we show that highly transcribed genes near *parS* that are oriented against SMC translocation disrupt arm alignment, suggesting that head-on transcription interferes with SMC translocation. Our results demonstrate a tight interdependence of bacterial chromosome organization and global patterns of transcription.

INTRODUCTION

The chromosomes of all organisms must be compacted nearly three orders of magnitude to fit within the limited volume of a cell. However, DNA cannot be haphazardly packed, and instead, it must be organized in a way that is compatible with numerous cellular processes that share the same DNA template, including transcription, DNA replication, and chromosome segregation. This is particularly challenging in bacteria because these chromosome-based transactions happen concomitantly rather than being separated temporally, as in eukaryotes. Application of microscopy-based analyses of fluorescently labeled DNA loci along with genome-wide chromosome conformation capture assays (Hi-C) have revealed a well-defined, in vivo, three-dimensional organization of bacterial chromosomes (Badrinarayanan et al., 2015a; Le et al., 2013; Umbarger et al., 2011; Viollier et al., 2004). Hi-C provides quantitative information about the spatial proximity of DNA loci in vivo by measuring the frequencies of crosslinking between different regions of the chromosome (Lieberman-Aiden et al., 2009). The first application of Hi-C to bacteria examined the *Caulobacter crescentus* chromosome (Le et al., 2013). Hi-C analysis of *Caulobacter* confirmed the global pattern of chromosome organization: in cells with a single chromosome, the origin of replication (*ori*) is at one cell pole, the terminus (*ter*) is near the opposite pole, and the two chromosomal arms are well-aligned, running in parallel down the long axis of the cell (Le et al., 2013; Viollier et al., 2004; Figure 1A). We discovered that a structural maintenance of chromosomes protein (SMC) is crucial for the alignment of the left and right arm of the chromosome in *Caulobacter* (Le et al., 2013), but how SMC achieves this alignment remains poorly understood.

SMC proteins are widely conserved from bacteria to humans. In eukaryotes, SMC1 and SMC3, together with accessory proteins, form a cohesin complex that holds sister chromatids together until after they achieve bipolar attachment to the mitotic spindle. The related condensin complex, comprised of SMC2, SMC4, and interacting partners, promotes the compaction of individual chromosomes during mitosis. In most bacteria, there is a single SMC composed of an ATPase "head" domain, a dimerization "hinge" domain, and an extended antiparallel coiled-coil region in the middle (reviewed in Nolivos and Sherratt, 2014). Two SMC monomers dimerize and, together with the bacteria-specific proteins ScpA and ScpB, form a tripartite ring that can bring distal DNA segments close together to help organize bacterial chromosomes (Bürmann et al., 2013; Mascarenhas et al., 2002). The topological entrapment of DNA by a ring-shaped SMC complex has been shown for cohesin and condensin and for *Bacillus subtilis* SMC (Cuylen et al., 2011; Ivanov and Nasmyth, 2005; Wilhelm et al., 2015). It is likely that topological entrapment is a general feature of all SMC complexes.

How SMC gets loaded onto DNA and topologically entraps DNA is generally well studied in eukaryotes, but not yet completely understood (reviewed in Uhlmann, 2016). In bacteria, SMC loading, translocation, and DNA entrapment is less well known but likely involves the ParA-ParB-*parS* system (Gruber and

CrossMark

This is an open access article under the CC BY license (http://creativecommons.org/licenses/by/4.0/).

OPEN
ACCESS
CellPress

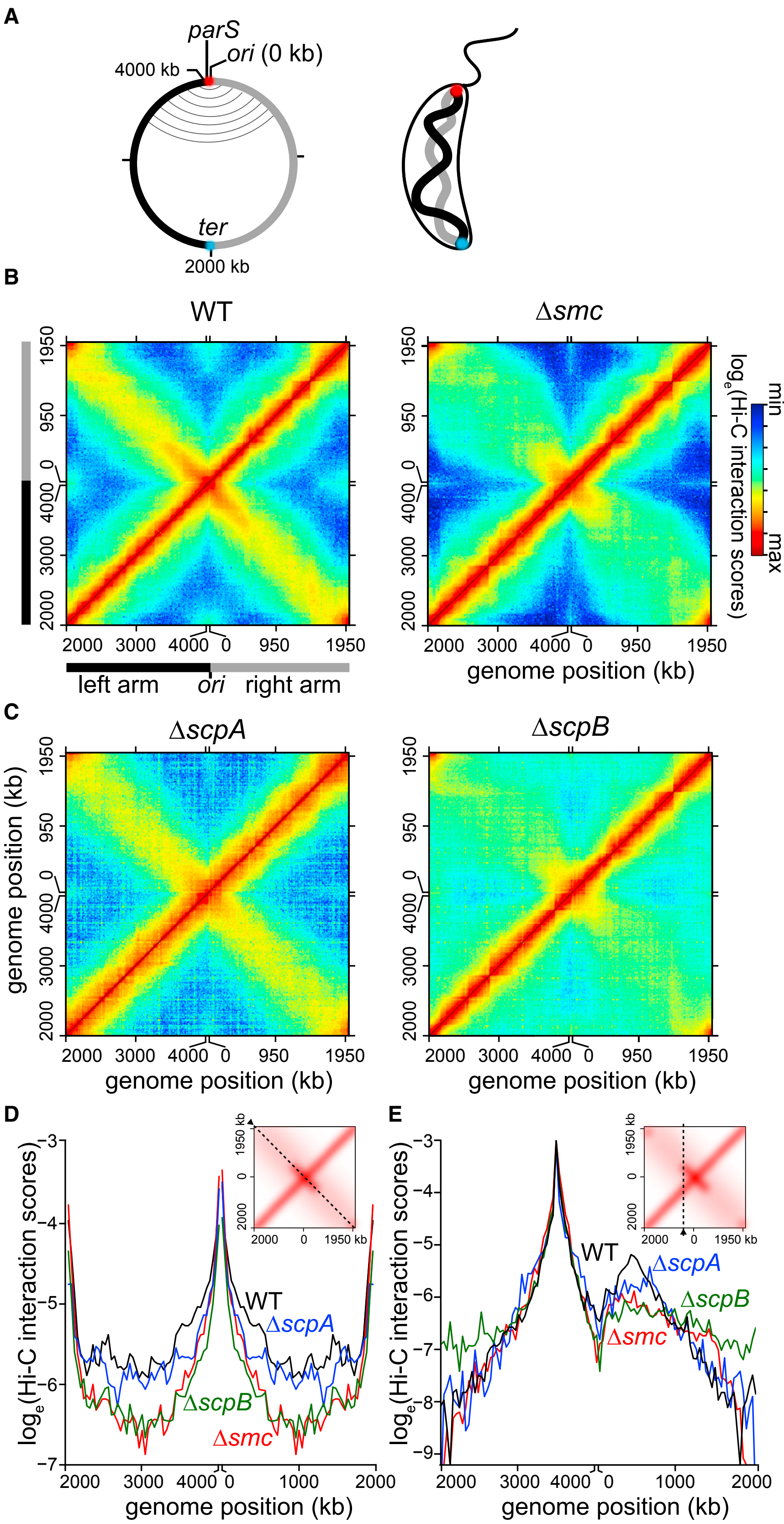

Figure 1. The SMC-ScpA-ScpB Complex Promotes the Alignment of Chromosomal Arms

(A) A simplified genomic map of *Caulobacter* showing the origin of replication (*ori*), the *parS* site, and the terminus (*ter*), together with left (black) and the right (gray) chromosomal arms. On the genomic map, aligned DNA regions are presented schematically as gray curved lines connecting the two chromosomal arms. Spatially, *ori* (red) and *ter* (cyan) are at opposite poles, and the two arms run in parallel down the long axis of the cell.

(B) Normalized Hi-C contact maps showing the natural logarithm of DNA-DNA contacts for pairs of 10-kb bins across the genome of WT and *Δsmc* cells (Le et al., 2013).

(C) Normalized Hi-C contact maps for *ΔscpA* and *ΔscpB* cells.

(D) Hi-C interaction scores along the secondary diagonal (black dashed line in the inset) for contact maps of WT, *Δsmc*, *ΔscpA*, and *ΔscpB* cells. Bins near *ori* or near *ter* are dominated by intra-arm instead of inter-arm DNA-DNA interactions due to the circular nature of the chromosome.

(E) Hi-C interaction scores along the vertical line (black dashed line in the inset) for contact maps of WT, *Δsmc*, *ΔscpA*, and *ΔscpB* cells.

Errington, 2009; Lin and Grossman, 1998; Minnen et al., 2011). ParB is a DNA-binding protein that nucleates on a centromere-like *parS* sequence (Mohl and Gober, 1997) and then spreads non-specifically along the DNA, likely forming a large nucleoprotein complex (Breier and Grossman, 2007; Graham et al., 2014). ParA, a Walker-box ATPase, interacts with ParB and is required for the segregation of replicated chromosomes to daughter cells (Figge et al., 2003). In *B. subtilis*, ParB loads SMC onto the chromosome mainly at the *ori*-proximal *parS* sites (Gruber and Errington, 2009; Marbouty et al., 2015; Wang et al., 2015). Loaded SMC then translocates from *parS* to distal parts of the chromosome in an ATP hydrolysis-dependent manner (Minnen et al., 2016; Wang et al., 2017). This action is thought to individualize the origins of replicated chromosomes, thereby helping to segregate replicated chromosomes. It has been proposed that *B. subtilis* SMC loaded at *parS* translocates the full length of the chromosome to *ter* to promote chromosome arm alignment (Gruber and Errington, 2009; Minnen et al., 2016; Wang et al., 2017). However, chromatin immunoprecipitation with deep sequencing (ChIP-seq) studies indicate that *B. subtilis* SMC is most enriched near *ori*, so whether SMC directly promotes arm alignment uniformly across the genome is unclear (Minnen et al., 2016; Wang et al., 2017).

Caulobacter harbors both a canonical SMC-ScpA-ScpB complex as well as a ParA-ParB-*parS* system. The ParA-ParB-*parS* complex is essential (Mohl and Gober, 1997). In contrast, *Caulobacter* SMC is not required for survival in laboratory conditions (Le et al., 2013). *Caulobacter* cells lacking SMC grow slightly slower but are not temperature sensitive or prone to accumulating suppressor mutations as originally suggested (Jensen and Shapiro, 1999). Nevertheless, ectopic overexpression of an ATPase-defective *smc* mutant shows a severe defect in sister chromosome segregation in this bacterium, consistent with *Caulobacter* SMC playing a role in chromosome segregation (Schwartz and Shapiro, 2011).

How SMC influences other cellular processes, like transcription, and is, in turn, influenced by these processes is not well understood. In yeast, cohesin is pushed along the chromosome in the same direction as transcription, without dissociating (Ocampo-Hafalla et al., 2016). Similarly, in budding yeast, RNA polymerase can drive the short-range relocation of condensin (D'Ambrosio et al., 2008; Johzuka and Horiuchi, 2007). Whether bacterial RNA polymerase affects SMC has not been systematically investigated. Notably, in almost all bacteria, most genes, especially highly expressed genes, are transcribed from *ori* to *ter* (reviewed in Rocha, 2008). This co-orientation of genes could help push SMC toward *ter*; alternatively, or in addition, genes transcribed in a head-on orientation could antagonize the translocation of SMC.

Here, we use Hi-C and ChIP-seq, together with microscopy-based analysis of single cells to elucidate the role and the mechanism of SMC in the global organization of the *Caulobacter* chromosome. We provide evidence that (1) SMC is required for the progressive alignment of the two chromosomal arms, proceeding in the *ori-ter* direction, (2) *Caulobacter* SMC is loaded onto the chromosome at the *parS* site and ParB is essential for the SMC-mediated arm alignment, (3) *Caulobacter* SMC most likely functions as a tether to actively cohese ~600-kb *parS*-proximal regions of the chromosomal arms together, and (4) head-on transcription can profoundly disrupt the alignment of chromosomal arms, likely by interfering with SMC translocation from *parS*. Altogether, our results demonstrate a tight interdependence of bacterial chromosome organization and global patterns of transcription.

RESULTS

The SMC-ScpA-ScpB Complex Is Required for the Alignment of Chromosomal Arms

Caulobacter cells lacking SMC show a dramatic reduction in inter-arm DNA-DNA interactions (Le et al., 2013; Figure 1B). To test whether the *Caulobacter* ScpA and ScpB homologs are also required for inter-arm interactions, we generated Hi-C contact maps for homogeneous G1-phase populations of Δ*scpA* and Δ*scpB* cells (Figures 1B and 1C). We divided the *Caulobacter* genome into 10-kb bins and assigned to corresponding bins the interaction frequencies of informative ligation products. Interaction frequencies were visualized as a matrix, with each matrix element, m_{ij}, indicating the natural logarithm of the relative interaction frequency of DNA loci in bin *i* with those in bin *j*. To emphasize the *ori*-proximal region, we oriented the Hi-C contact maps such that the *ori* (0 kb or 4,043 kb) is at the center of the x and y axis and the left and the right chromosomal arm are on either side (Figures 1B and 1C).

On the contact map of wild-type (WT) *Caulobacter*, the primary and high-interaction diagonal represents interactions between DNA on the same arm of the chromosome, i.e., intra-arm contacts, whereas the less prominent secondary diagonal represents DNA-DNA interactions between opposite arms, i.e., inter-arm contacts (Figure 1B). The diagonal pattern of inter-arm contacts on a Hi-C map indicates that each locus on one chromosomal arm interacts with a locus roughly equidistant from the *ori* on the opposite arm, reflecting a global alignment of the chromosomal arms. Consistent with our previous studies (Le et al., 2013), the inter-arm interactions are significantly reduced in a Δ*smc* strain. The Hi-C map for Δ*scpB* revealed a similar decrease in inter-arm interactions, with no strong or obvious change in intra-arm interactions (Figures 1C–1E). The Hi-C map for Δ*scpA* also exhibited a decrease in inter-arm interactions, though not nearly as significant as for Δ*smc* and Δ*scpB* (Figures 1C–1E). These data are consistent with ScpA and ScpB forming a complex with SMC that promotes the co-linearity of chromosomal arms in *Caulobacter*.

ParB Induces a Progressive Alignment of Chromosomal Arms from *ori* to *ter*

In Gram-positive bacteria, such as *B. subtilis* and *Streptococcus pneumoniae*, SMC is loaded onto the chromosome by ParB at *ori*-proximal *parS* sites (Gruber and Errington, 2009; Minnen et al., 2011). To test whether this mechanism is conserved in *Caulobacter*, a Gram-negative bacterium, we used a strain where ParB, which is essential for viability, can be depleted. The promoter of *parB* at its native chromosomal locus was replaced with a xylose-inducible promoter, P_{xyl}. Cells grown to exponential phase in the presence of xylose were then washed free of xylose and incubated for five hours in a rich medium supplemented with glucose to inhibit P_{xyl} activity. Immunoblot

OPEN
ACCESS
CellPress

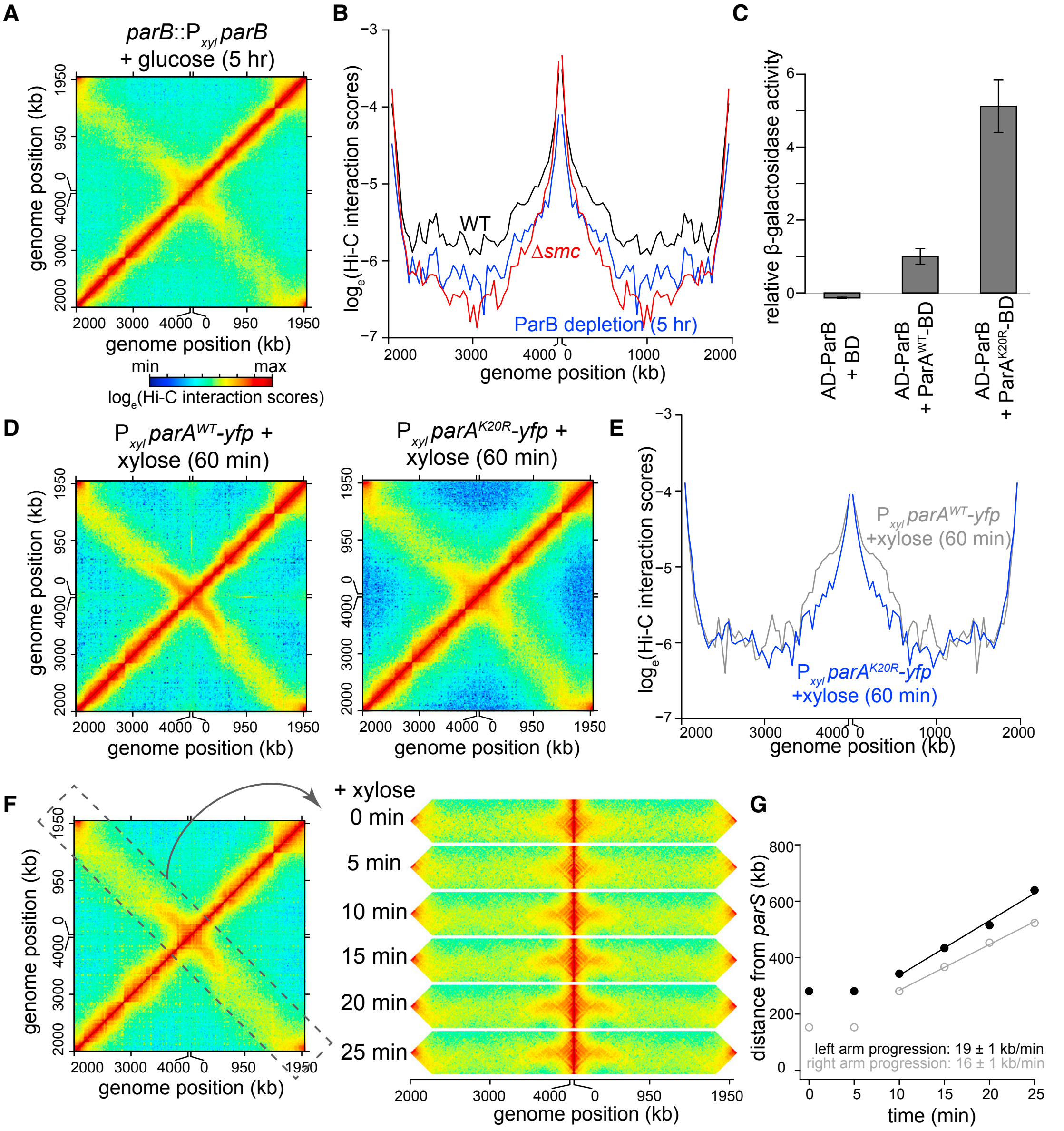

Figure 2. ParB Is Required for the Progressive Alignment of Chromosomal Arms by SMC

(A) Normalized Hi-C maps for *parB::P*$_{xyl}$ *parB* cells 5 hr after starting the depletion experiment.

(B) Hi-C interaction scores along the secondary diagonal for contact maps of WT (black), *Δsmc* (red), and *parB::P*$_{xyl}$ *parB* cells (blue) 5 hr after starting the depletion experiment.

(C) Yeast two-hybrid assay to compare ParB-ParA^{WT} interaction to that of ParB-ParA^{K20R}. The β-galactosidase activity was assayed for each strain and is presented relative to the value obtained for the ParB-ParA^{WT} interaction. Error bars represent SD from four biological replicates.

(D) Normalized Hi-C maps for cells overexpressing *parA*^{WT}*-yfp* and *parA*^{K20R}*-yfp* after adding xylose for 1 hr.

(E) Hi-C interaction scores along the secondary diagonal for contact maps of cells overexpressing *parA*^{WT}*-yfp* (gray) and *parA*^{K20R}*-yfp* (blue) after adding xylose for 1 hr.

(F) A time-resolved Hi-C for cells that are replenishing of ParB. Time after adding back xylose was indicated next to each Hi-C strip. For presentation purposes, the secondary diagonal (black dashed box) was rotated and laid out horizontally.

(G) Analysis of the progression of chromosomal arm alignment. The extent of DNA alignment at each time point after adding back xylose was plotted for each chromosomal arm. The black and gray lines are linear best fit lines for data from time point 10 min to 25 min.

analysis with an α-ParB antibody indicated ~2.5-fold decrease in ParB concentration after the five hours in glucose (T = 0 min; Figure S1A). The contact map of ParB-depleted cells exhibited a clear reduction in inter-arm contacts, similar to Δ*smc* cells, indicating a role of ParB in maintaining chromosomal arm alignment, possibly by loading SMC onto DNA (Figures 2A and 2B).

As with *B. subtilis* SMC (Gruber and Errington, 2009; Wang et al., 2015), the *Caulobacter* SMC complex may be recruited and loaded onto DNA via a direct interaction with ParB. If so, we reasoned that overexpressing a strong ParB-interacting protein might prevent interactions with SMC and, in turn, disrupt alignment of the chromosomal arms. To test this hypothesis, we performed Hi-C on cells overexpressing a YFP-tagged variant of ParA harboring a K20R substitution. ParA(K20R) is defective in ATP binding but binds ParB more tightly than ParA (WT), at least in a yeast two-hybrid assay (Figure 2C; Shebelut et al., 2010). The Hi-C contact map for a strain overexpressing ParA(K20R) showed a modest but significant decrease in chromosomal arm alignment compared to a control strain overexpressing ParA (WT) ($p < 10^{-12}$; paired Student's t test; Figures 2D, 2E, S1B, and S1C). This result reinforces our conclusion that ParB is required for the SMC-mediated alignment of chromosomal arms in *Caulobacter*.

To investigate the directionality and dynamics of chromosome arm alignment, we again depleted ParB by growing cells in rich medium with glucose for 5 hr and then added back xylose to induce ParB de novo. Samples were taken 0, 5, 10, 15, 25, and 30 min after xylose addition for Hi-C analysis. Immunoblot analysis with α-ParB antibody showed gradual accumulation of new ParB after adding back xylose (Figure S1A). At the 0- and 5-min time points, we observed very little inter-arm interaction, as above (Figures 2F, S1D, and S1E). However, over time, the inter-arm interactions increased, beginning close to *ori* and *parS* and then extending toward *ter* (Figures 2F, 2G, S1D, and S1E). The two arms aligned directionally at a rate of ~19 kb per minute and ~16 kb per minute for the left and right arm, respectively (Figures 2F and 2G). These data are consistent with a model in which SMC is loaded by ParB, likely at *ori*/*parS*, and then translocates down the arms toward *ter*, driving their alignment. Nevertheless, we cannot formally exclude the possibility that ParB might have additional, SMC-independent roles in chromosomal arm alignment.

The *parS* site is ~8 kb from *ori* in *Caulobacter* (Toro et al., 2008). To directly test the model that SMC is loaded onto DNA at *parS* sites, we inserted a 260-bp DNA fragment containing a *parS* site either at +1,800 kb or +2,242 kb from *ori* (Figure S2A). We verified that this ectopic *parS* site was sufficient to recruit ParB, using ChIP-seq analysis of ParB, which binds to the native *parS* site (Figure S2B). We then performed Hi-C on the strain harboring the additional *parS* site and observed a new secondary diagonal, emanating from the approximate position of the ectopic *parS* site (Figure S2C). The extent of alignment from this ectopic site was less than that associated with the original *parS*, an issue examined in depth below. Taken together, our results support a model in which ParB loads SMC at *parS* sites, leading to the subsequent progressive alignment of flanking DNA.

SMC Promotes DNA Alignment Most Effectively for *parS*-Proximal Genomic Regions

After being loaded at *parS* sites, SMC likely translocates down the chromosomal arms to drive their alignment. However, the extent of inter-arm interactions was not uniform across the entire chromosome, indicating that each loaded SMC complex may not travel the entire length of the chromosome. In fact, we noted that, in WT cells, inter-arm interactions reduced gradually away from *parS*, leveling out after ~600 kb in either direction (Figure 1D). These observations suggested that SMC is not uniformly distributed across the chromosome and instead may be enriched in the regions showing highest inter-arm interactions. To test this hypothesis, we first used ChIP-seq to map the genome-wide distribution of epitope-tagged SMC. We fused the SMC-encoding gene to a FLAG tag at the N terminus and placed this allele downstream of P_{xyl} on a medium-copy-number plasmid. We then performed Hi-C on *Δsmc* cells that produced this FLAG-tagged SMC via leaky expression from P_{xyl} (Figure S3A). Chromosomal arm alignment was comparable to the WT level (Figures S3B and S3C), indicating that FLAG-SMC is functional. Overproducing FLAG-SMC in the *Δsmc* background by adding xylose did not extend arm alignment beyond the WT level (Figures S3B and S3C). We performed α-FLAG ChIP-seq with the *Δsmc* P_{xyl}-*flag-smc* strain. As a negative control, we performed α-FLAG ChIP-seq on WT *Caulobacter*, i.e., cells with untagged SMC (Figure S3D). Although only a small percentage of ChIP DNA is enriched by FLAG-SMC, we observed a clear enrichment of SMC-bound DNA near the *parS* site (Figure 3A), consistent with SMC loading at *parS*. SMC decreases in enrichment away from *parS* but is enhanced close to the *ter* area (Figure 3A; Discussion). We also noted the enrichment of SMC at highly transcribed genes (Figures 3A and 3B), most likely as an artifact of non-specific immunoprecipitation (Teytelman et al., 2013).

Notably, FLAG-tagged SMC was enriched above background in a region overlapping *parS* that extended from approximately +3,680 kb to +345 kb, the same approximate region that shows the most extensive inter-arm alignment by Hi-C (Pearson's correlation coefficient = 0.75; $p < 10^{-12}$; Figures 3A and S4A). We could not detect significant enrichment of FLAG-tagged SMC beyond this *parS*-proximal region, indicating that SMC is either not appreciably bound to *parS*-distal regions of the chromosome or its association drops below our limit of detection with ChIP-seq. In either case, we conclude that (1) SMC is not uniformly distributed across the genome and (2) the enrichment of SMC correlates with the extent of chromosome arm alignment at the *ori*-proximal region.

To further test the relationship of SMC enrichment and the alignment of flanking DNA, we generated a strain bearing a relatively large inversion (involving genomic DNA normally between +3,611 and +4,038 kb) such that *parS* is relocated ~427 kb away from *ori*; this strain is referred to as the Flip 1-5 strain (Figure 3B). ChIP-seq of FLAG-tagged SMC in the Flip 1-5 inversion strain showed an enrichment of DNA surrounding the relocated *parS* site at a peak level comparable to the *ori*-proximal *parS* (Figures 3A and 3B), further supporting the conclusion that SMC is loaded at *parS* and that SMC enrichment near *parS* is independent of *ori*.

The Hi-C contact map of the Flip 1-5 inversion strain also showed a secondary diagonal (Figures 3C and S5A). However, the starting point of the flanking DNA alignment was shifted and coincided with the genomic position of the relocated *parS* (Figures 3C and S5A). This ectopic arm alignment was reduced dramatically in the absence of *smc* (Figures 3C and 3D),

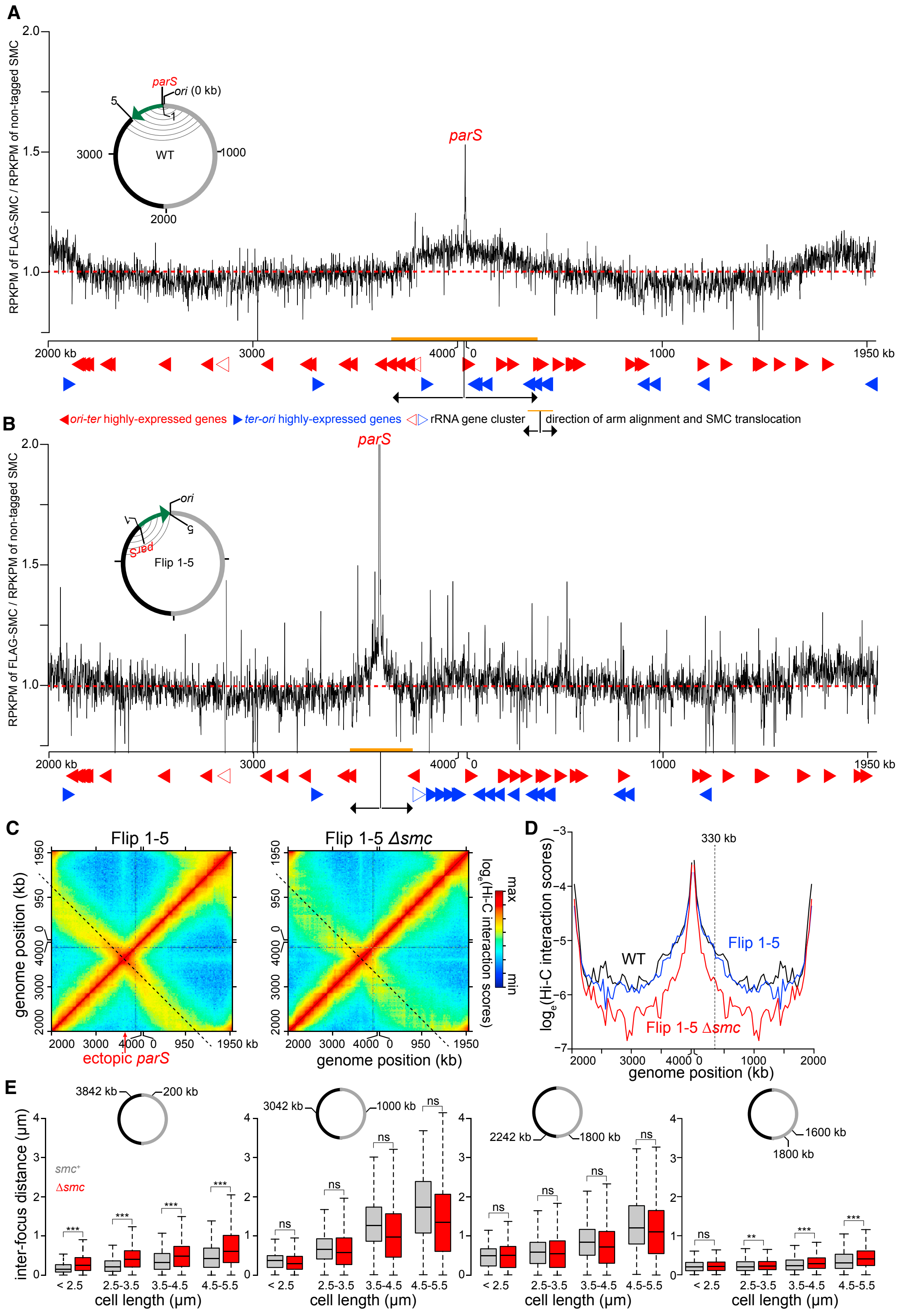

(legend on next page)

indicating that arm alignment in the Flip 1-5 strain still depends on SMC. For the Flip 1-5 strain, the inter-arm alignment was again strongest in a limited region around *parS*, extending approximately 330–420 kb in each direction (Figures 3D and S5B). The extent of arm alignment in the Flip 1-5 strain was reduced compared to WT cells (~600 kb), consistent with the reduced region showing SMC enrichment by ChIP-seq (Figures 3B and S4B).

Collectively, the data presented thus far suggest that SMC loaded at *parS* may only translocate a limited distance down the chromosome. If so, we predicted that if the chromosome expanded, as occurs in elongating, G1-arrested cells, then *parS*-proximal regions would (1) remain better aligned than other chromosomal regions and (2) exhibit a stronger dependence on *smc* for alignment (Figure S5C). To test this model, we fluorescently labeled pairs of DNA loci, at equivalent distances from *ori*, but on opposite arms of the chromosome, using an orthogonal ParB/*parS* system (Badrinarayanan et al., 2015b). A pair of DNA loci were engineered at +200 kb and +3,842 kb, i.e., within the *parS*-proximal domain showing strongest SMC enrichment and highest inter-arm Hi-C values. Another pair of DNA loci was labeled in the middle of each arm (+1,000 kb and +3,042 kb). And finally, to investigate chromosomal arm alignment near *ter*, a pair of DNA loci at +1,800 kb and +2,242 kb were fluorescently labeled. We then measured, for each pair of loci, inter-focus distances in a population of otherwise WT and Δ*smc* cells (Figure 3E). To allow cells to elongate and expand their chromosomes, we measured inter-focus distances in cells where the only copy of *dnaA* was driven by a vanillate-regulated P_{van} promoter. Washing cells free of vanillate produced a population of cells that each contained just one copy of the chromosome and continued to grow but were unable to divide, leading to an elongated cell where the chromosome fills the entire available cytoplasmic space (Le and Laub, 2016).

As cell length increased with time, we observed that the mean inter-focus distances for each pair of DNA loci also increased, consistent with an overall expansion of the chromosome (Figure 3E). However, the rate of expansion was different, depending on genomic locations of labeled DNA loci. The inter-focus distance for the *parS*-proximal, 200- to 3,842-kb pair increased only modestly, ~0.5 μm as the cell length tripled (Figure 3E). In contrast, the inter-focus distance for mid-arm loci increased ~1.5 μm on average as the cell length tripled (Figure 3E). Importantly, the inter-focus distance of the *parS*-proximal loci, but not the other locus pairs, increased in the Δ*smc* background (Figure 3E). These microscopy-based analyses support our hypothesis that SMC most effectively aligns the chromosome arms nearest the *ori* and *parS* site where it is loaded.

In parallel, we analyzed Hi-C data on the elongated cells resulting from DnaA depletion (Figures S5D and S5E; Le and Laub, 2016). We observed that ~300 kb surrounding *parS* remains well aligned, even in elongated cells 3 hr after starting the depletion (Figures S5D and S5E). In contrast, chromosomal arm alignment elsewhere on the chromosome was rapidly lost to the same extent as in Δ*smc* cells 2 hr after starting the depletion (Figures S5F and S5G). These results agree well with the single-cell microscopy results and together suggest that *Caulobacter* SMC functions most effectively to align chromosomal arms near *ori*/*parS*.

Conflicts with Transcription Influence the SMC-Mediated Alignment of Chromosomal Arms

We noted that the region showing the strongest inter-arm interactions did not extend as far from *parS* in the Flip 1-5 strain or in the strains harboring the ectopic *parS* sites near *ter* (Figures 3C, 3D, S5A, and S5B). These observations suggested that the genomic context surrounding *parS* may influence the translocation of SMC along each arm of the chromosome and, consequently, the patterns of inter-arm interaction.

We hypothesized that highly expressed genes, particularly those oriented toward *parS*, may limit the translocation of SMC. To test this idea, we engineered a strain, hereafter called Flip 2-5, in which a ~419-kb DNA segment between +3,611 kb and +4,030 kb on the left arm was inverted (Figure 4A). This inversion leaves *parS* at its original location but dramatically changes the genomic context of the flanking DNA on one side of *parS* while leaving the other side unperturbed (Figures 4A, S6A, and S6B). The inverted segment contains several highly

Figure 3. SMC Is Enriched at the *parS* Site and Promotes DNA Alignment Most Effectively for *parS*-Proximal Regions

(A) The distribution of FLAG-tagged SMC on WT *Caulobacter* chromosome. ChIP-seq signals were reported as the number of reads within every 1-kb bin along the genome (RPKPM) in the ChIP fraction of FLAG-tagged SMC divided by untagged SMC. The dashed red line shows y axis value at 1. Below the ChIP-seq profile is the position of highly expressed genes that transcribe in the *ori*-*ter* (solid red arrows) or *ter*-*ori* (solid blue arrows) direction. The positions of rRNA gene cluster are indicated with open red or blue arrows. The direction and extent of SMC translocation from *parS* site were shown as black arrows and orange bar, respectively. A schematic genomic map of *Caulobacter* showing the position of *parS* (red) and *ori* are presented in the inset. The inverted DNA segment (green arrow) is indicated together with the end points of the inversion (1 and 5). On the genomic map, aligned DNA regions, as observed by Hi-C, are presented schematically as gray curved lines connecting the two chromosomal arms.

(B) The distribution of FLAG-tagged SMC on the chromosome of Flip 1-5 *Caulobacter*.

(C) Normalized Hi-C maps for the Flip 1-5 and Flip 1-5 Δ*smc* cells.

(D) Hi-C interaction scores along the secondary diagonal for contact maps of WT (black), Flip 1-5 (blue), and Flip 1-5 Δ*smc* cells (red). The Hi-C interaction scores along the secondary diagonal of Flip 1-5 and Flip 1-5 Δ*smc* cells (black dashed lines in C) were shifted to the same position as that of WT to enable comparison between strains. The vertical black dashed line at ~330 kb away from *ori* shows the position where Hi-C interaction scores along the secondary diagonal start to reduce in Flip 1-5 strain in comparison to WT.

(E) Inter-focus distances expand differentially in elongated *Caulobacter* cells, depending on their genomic locations. Pairs of DNA loci were labeled with YFP-$ParB^{pMT1}$/$parS^{pMT1}$ and mCherry-$ParB^{P1}$/$parS^{P1}$ near *ori* (+200 kb and +3,842 kb), at the middle of each arm (+1,000 kb and +3,042 kb), near *ter* (+1,800 kb and +2,242 kb), or on the same arm (+1,600 kb and +1,800 kb). Boxplots show the distribution of inter-focus distances for cells of different sizes with SMC (gray) or without (red) SMC. Asterisks indicate statistical significance (***$p < 0.001$; ns, not significant; one-tailed Student's t test; null hypothesis: inter-focus distance in Δ*smc* is greater than in WT cells).

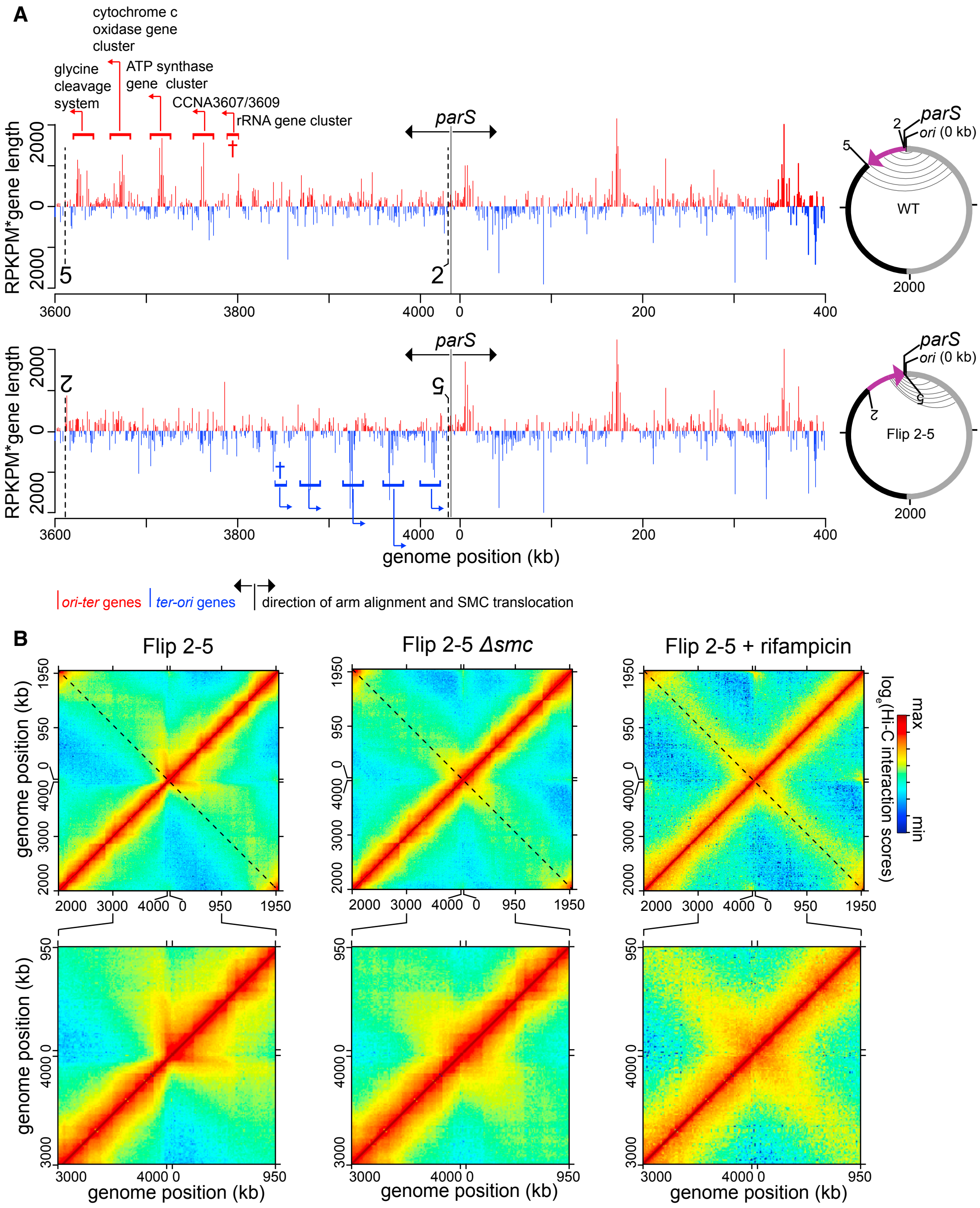

Figure 4. Genomic Context and Transcription Influence the SMC-Mediated Alignment of Chromosomal Arms

(A) The abundance of RNA polymerases on genes that transcribe in the *ori-ter* direction (red) or in the *ter-ori* direction (blue) in WT and Flip 2-5 cells for the DNA segment between +3,600 kb and +400 kb. The position of *parS* and the direction of SMC translocation are indicated with black arrows. For the whole genome plot, see Figure S6. ChIP-seq using α-FLAG antibody was performed on cells expressing *rpoC-flag* from its native locus in WT background (upper panel) or in Flip 2-5 background (lower panel). The abundance of RNA polymerases was represented as RPKPM × gene length for each gene and plotted against the genomic location of that gene. Due to short sequencing reads and the high similarity between the two rRNA clusters, it is not reliable to estimate the RNA polymerase density within each rRNA cluster. Therefore, enrichment data for rRNA gene clusters are not shown. Nevertheless, we indicate the genomic position of a highly expressed rRNA cluster on *Caulobacter* genome with a dagger (†) symbol. Vertical black dashed lines with numbering 2 and 5 indicate the inversion end points.

(B) Normalized Hi-C contact maps for Flip 2-5, Flip 2-5 *Δsmc* cells, and Flip 2-5 cells treated with rifampicin. A 1,000-kb region surrounding *parS/ori* was also zoomed in and presented below each whole-genome Hi-C contact map.

expressed genes, including a rRNA gene cluster and genes encoding ATP synthase, the glycine cleavage system, and cytochrome *c* oxidase, that all normally read in the *ori-ter* direction, i.e., co-directionally with SMC translocation (Figures 4A, S6A, and S6B; Le and Laub, 2016).

To assess the density of RNA polymerases directly, we performed ChIP-seq on exponentially growing cells producing RpoC-FLAG as the only version of RNA polymerase β' subunit (Figures 4A, S6A, and S6B). Separating sequencing reads based on the direction of transcription clearly demonstrated an enrichment of highly expressed genes transcribed in the *ori-ter* direction in WT cells between +3,611 and +4,030 kb (Figures 4A and S6A). We also confirmed, using ChIP-seq on RpoC-FLAG, that these same genes remain highly expressed in the Flip 2-5 background but now on the opposite strand such that they read in a *ter-ori* direction (Figures 4A and S6B).

The Hi-C contact map of G1-phase Flip 2-5 cells showed, in sharp contrast to WT cells, a pronounced asymmetrical pattern of inter-arm interactions (Figures 4B, S7A, and S7B). The inter-arm interactions in the Flip 2-5 strain manifested as a nearly vertical streak in the Hi-C map, still emanating from a *parS*-proximal position (Figure 4B). This vertical streak indicates that the strongest inter-arm interactions now occur between an ~50- to 80-kb region of DNA on the left side of *parS* with an ~400-kb segment on the right arm of the chromosome, a pronounced asymmetry compared to the pattern in WT cells (Figure 4B). We confirmed that this asymmetric pattern of inter-arm interactions in the Flip 2-5 strain is still dependent on SMC as this vertical streak disappeared from the contact map of Flip 2-5 *Δsmc* cells (Figure 4B). These results suggest that the genomic context of DNA flanking the *parS* site, and likely the orientation of highly expressed genes, can dramatically influence the pattern of inter-arm contacts.

To assess whether the Flip 2-5 strain also led to a change in the genomic distribution of SMC, we performed ChIP-seq on FLAG-SMC in the Flip 2-5 background (Figures 5 and S4C; Pearson's correlation coefficient between ChIP-seq and Hi-C data = 0.6; $p < 10^{-12}$). The enrichment of SMC dropped off slightly faster in the first ~150 kb away from *parS*, in the *ori-ter* direction, in the Flip 2-5 strain compared to WT (Figures 5A and S7C). However, most strikingly, the Flip 2-5 strain exhibited a series of new SMC ChIP-seq peaks, in addition to the one at *parS*, particularly in the region that was inverted (solid triangles, Figures 5A–5C). Comparing the SMC and RpoC ChIP-seq profiles indicated that these new SMC peaks coincided with the highly expressed gene clusters that had been reoriented in the inversion to read toward *ori* and *parS* (Figure 5C). These new peaks are not artifacts that are normally associated with highly transcribed genes because (1) they are unique in Flip 2-5 cells, but not in the WT or Flip 1-5 control, and (2) the shape and the magnitude of the unique peaks in Flip 2-5 are distinct from that of WT and the Flip 1-5 cells (Figure 5C). These data suggested that transcription in a head-on orientation with SMC translocation can either impede SMC translocation away from *parS*, unlink potential handcuffed SMC, or drive the dissociation of SMC from DNA, limiting the extent of inter-arm contacts and, in some cases, produce an asymmetrical pattern of inter-arm contacts by Hi-C.

To test whether the asymmetrical inter-arm interactions in the Flip 2-5 strain arise because of the reoriented, highly expressed genes within the inverted genomic region, we treated Flip 2-5 cells with rifampicin before fixing cells for Hi-C (Figure 4B). Rifampicin inhibits transcription elongation in bacteria, thereby eliminating actively translocating RNA polymerases from the chromosome. As we reported previously for WT cells, the inhibition of transcription reduced short-range intra-arm contacts (Le et al., 2013). In addition, for the Flip 2-5 strain, the vertical streak was eliminated and the inter-arm interactions reverted to a symmetric pattern on the diagonal (Figures 4B and S7B), demonstrating that transcription is required for an asymmetrical inter-arm interaction pattern.

To further test the relationship between the orientation of highly expressed genes and SMC-dependent inter-arm interactions, we constructed three additional strains with different chromosomal inversions (Figure 6A). We wondered whether reversing the transcription orientation of a single highly transcribing rRNA gene cluster would be sufficient to induce an asymmetrical inter-arm pattern. To test this possibility, we created the Flip 3-4 strain (Figure 6A). The Hi-C map of G1-phase cells of the Flip 3-4 strain showed negligible changes to the inter-arm alignment compared to non-flipped cells (Figures 6B and 6C). However, the inverted region in this strain is ~240 kb away from *parS* and, as noted above, SMC and SMC-dependent inter-arm interactions are strongest within a limited range around *parS*. Thus, we reasoned that the effect of an inverted rRNA cluster would be stronger if placed closer to *parS*. We did so by constructing the Flip 2-4 strain that has DNA between +3,788 kb and +4,030 kb inverted (Figure 6A). Hi-C on G1-phase cells of the Flip 2-4 strain showed a pronounced asymmetrical inter-arm interaction pattern (~20° deviation from a diagonal, starting after 80 kb from *ori*), though less dramatic than that of the Flip 2-5 cells, in which several highly expressed genes in addition to the rRNA locus were inverted (Figures 4B, 6B, and 6C).

We further investigated the effect of transcription orientation bias on chromosomal arm alignment by inverting a DNA segment between +3,611 kb and +3,788 kb, the Flip 4-5 strain. Although this section does not contain an rRNA gene cluster, it includes four highly transcribed operons that normally transcribe in the *ori-ter* direction in WT cells (Figure 6A; denoted with square brackets in Figure 4A). Preceding this segment, DNA between +3,788 kb and +4,030 kb is largely free of highly expressed genes oriented toward *ori*/*parS* (Figure 4A). Hi-C on G1-phase cells of the Flip 4-5 strain showed two distinct phases of inter-arm contacts (Figures 6B and 6C). The first phase (~290 kb) is a typical set of symmetrical inter-arm contacts as seen in WT cells. The second phase coincides with the inverted DNA segment and has a pronounced asymmetrical inter-arm pattern (Figures 6B and 6C). Collectively, our results emphasize that highly transcribed genes, depending on their transcriptional direction, can dramatically influence the action of SMC and the global organization of a chromosome.

DISCUSSION

Conflicts between SMC and RNA Polymerase and the Consequences for Bacterial Chromosome Organization

Chromosomes in all organisms are typically laden with DNA-bound proteins that likely influence the dynamics and movement

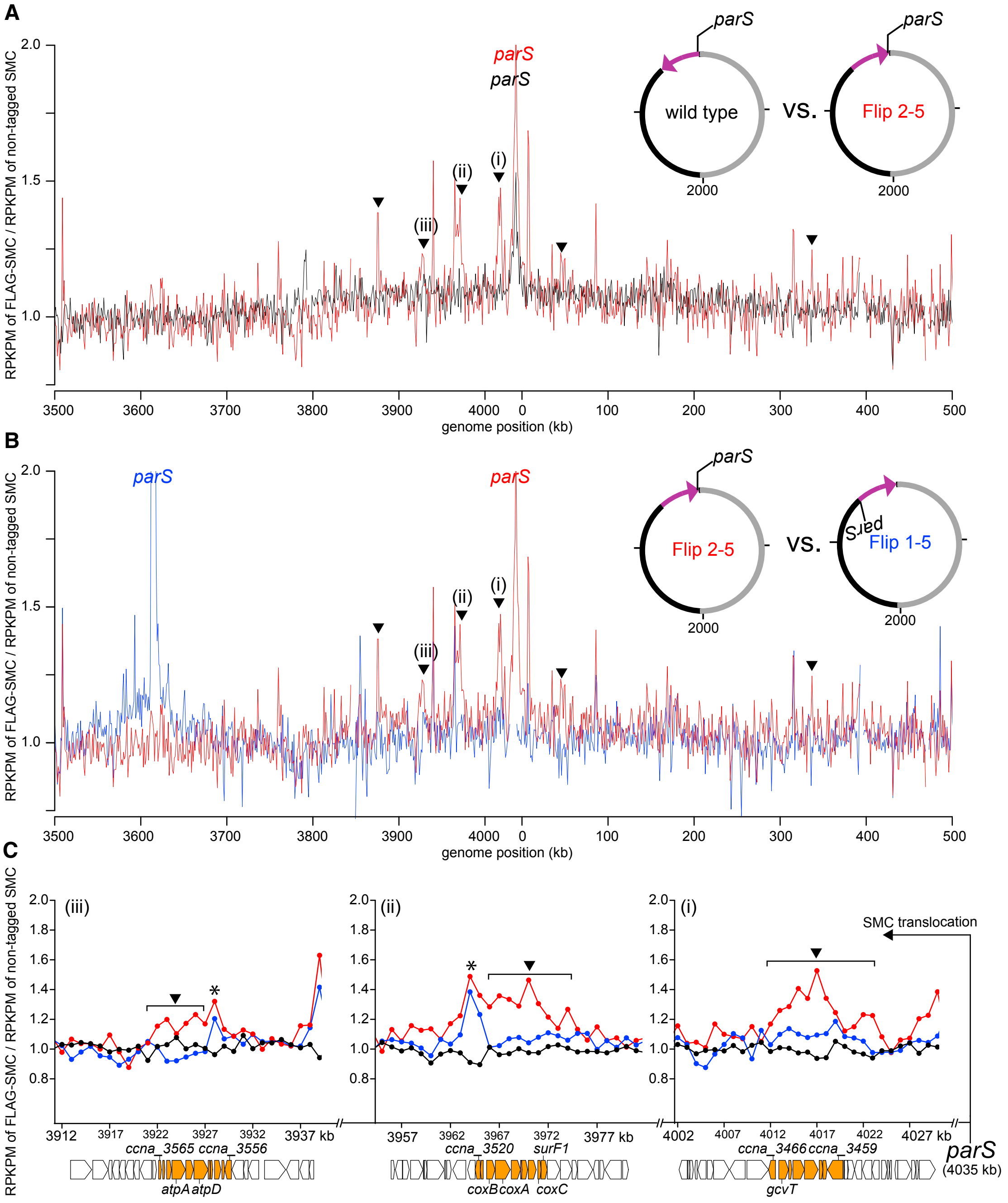

Figure 5. Head-On Transcription Alters the Distribution of SMC on the Chromosome

(A) The distribution of FLAG-tagged SMC on WT *Caulobacter* chromosome (black) and on Flip 2-5 chromosome (red). Only DNA segment between +3,500 kb and +500 kb was shown. For profiles of the whole genome, see Figure S4.

(B) The distribution of FLAG-tagged SMC on Flip 1-5 (blue) and Flip 2-5 chromosome (red). Black triangles indicate new peaks in the ChIP-seq profile of Flip 2-5, but not in the profiles of WT or Flip 1-5 strain. The ChIP-seq profile of FLAG-SMC in Flip 1-5 strain was slightly shifted to align to that of Flip 2-5 strain because the inverted DNA segment in Flip 1-5 is larger than in Flip 2-5 by 8 kb to encompass the native *parS* region (see Figures 4A and 4B).

(C) The distribution of FLAG-tagged SMC at the glycine cleavage system gene cluster (i, highlighted in orange), the cytochrome *c* oxidase gene cluster (ii, highlighted in orange), and the ATP synthase gene cluster (iii, highlighted in orange). The genomic position of *parS* and the direction of SMC translocation are shown with a black arrow. The genomic positions on the x axis and the gene direction are those of Flip 2-5 strain. We inverted in silico ChIP-seq profile and gene orientation of WT strain to enable comparison of superimposed ChIP-seq profiles. Black triangles indicate new peaks in the ChIP-seq profile of Flip 2-5, but not in the profiles of WT or Flip 1-5 strain. Black asterisks (*) indicate non-specific peaks that often associate with “hyper-ChIPable” regions at highly transcribed genes.

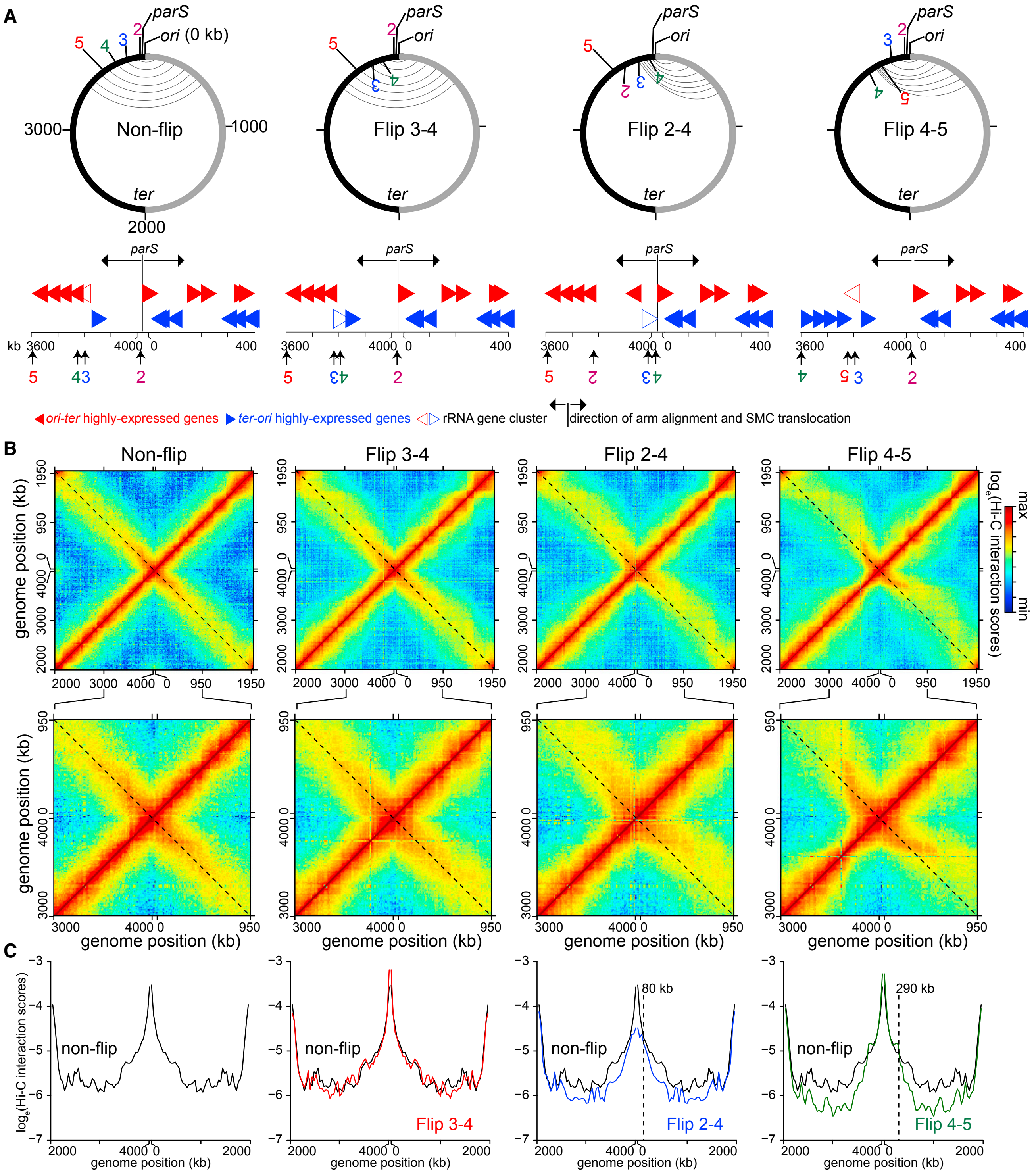

Figure 6. Inverting 180-kb DNA Segment Containing Highly Expressed *ori-ter* Genes Is Sufficient to Induce an Asymmetrical Pattern of Inter-arm Contacts

(A) Schematic genomic maps for WT cells (non-flip) and the inversion strain Flip 3-4, Flip 2-4, and Flip 4-5. The inversion end points (2, 3, 4, and 5), together with the genomic location of *parS* and *ori*, are indicated on the map. The aligned DNA regions, as observed by Hi-C (B), are presented schematically as gray curved lines connecting the two chromosomal arms. Below each genomic map are the positions of highly expressed genes that transcribe in the *ori-ter* (solid red arrows) or *ter-ori* (solid blue arrows) direction in each strain. The positions of rRNA gene cluster are indicated with open red or blue arrows. The genomic position of *parS* and the direction of SMC translocation are shown with black arrows. Only 400-kb DNA segments surrounding *ori* are shown.

(legend continued on next page)

of SMC (Ocampo-Hafalla et al., 2016; Stigler et al., 2016; Wang et al., 2017). Our results here indicate that the distribution and translocation of *Caulobacter* SMC, and the consequent alignment of chromosomal arms, is strongly influenced by actively transcribed genes, particularly those oriented toward *ori*. The observed chromosome organization defect likely stems from a conflict between SMC and transcription, without involving the replisome. This conclusion is based on two key observations. First, the experiments involving strains with inverted chromosomal regions were performed on synchronous G1-phase cells, i.e., non-replicating cells. Second, the Flip 1-5 strain, where the SMC loading site was relocated to a mid-arm position, has highly expressed genes (such as the rRNA gene cluster), transcribing in the opposite direction to that of the replisome in actively replicating cells. However, this strain did not exhibit a dramatic off-diagonal pattern of DNA interactions (Figure 3C). Thus, we suggest that head-on conflicts between translocating SMC and RNA polymerase can directly shape bacterial chromosome organization.

The mechanism(s) that drive translocation of bacterial SMC from *parS* remains unknown. It is tempting to speculate that the *ori*-*ter* transcription bias (Figure S6A) might indicate that transcription helps push SMC away from its *ori*-proximal loading site. However, chromosome arm alignment in WT *Caulobacter* cells treated with rifampicin is not reduced (Figures S7D and S7E). *E. coli* MukBEF, a non-canonical SMC, has been proposed to translocate via a "rope climber" mechanism (Badrinarayanan et al., 2012). In this model, a concerted opening and closing of just one MukBEF dimer in a handcuffed dimer-dimer allows an opened dimer to grab the next DNA segment before releasing the previously closed MukBEF, thereby "swinging" the dimer-dimer complex down DNA (Badrinarayanan et al., 2012). In *B. subtilis*, recent structural studies suggested that ATP binding, hydrolysis, and release can switch SMC between a rod- and a ring-like conformational state, with this motor-like cycling somehow mediating SMC translocation (Bürmann et al., 2013; Minnen et al., 2016). However, none of these current models can explain the *directional* movement of SMC from its loading site.

SMC Loading at the Bacterial Centromere *parS* Site: A Coupling between Chromosome Organization and Segregation

Our results support a model in which *Caulobacter* SMC is, like in *B. subtilis*, recruited at or near *parS*, loaded in a ParB-dependent manner, and then redistributed toward *ter*. ParB is a bacterial-specific protein but likely works closely with and coevolves with SMC to ensure chromosome organization and chromosome segregation in bacteria. Interestingly, *S. pneumoniae* lacks a ParA homolog, yet retains ParB-*parS* to recruit SMC (Minnen et al., 2011), underscoring the tight connection of ParB and SMC. It is worth noting that, unlike *B. subtilis*, deleting *smc* in a wide range of bacteria, including *Caulobacter*, does not cause sensitivity to high temperature or fast-growing conditions (Le et al., 2013; Nolivos and Sherratt, 2014). Conversely, however, ParA-ParB-*parS* is essential in *Caulobacter*, but not in *B. subtilis* (Mohl and Gober, 1997; Murray and Errington, 2008). The ParA-ParB-*parS* system and the SMC complex likely collaborate to ensure proper chromosome segregation and organization but with slightly different contributions or relative weights in different organisms. Finally, there is a distant SMC homolog, called the MukBEF system, in γ-proteobacteria (Nolivos and Sherratt, 2014). Notably, ParA-ParB-*parS* systems do not exist in these bacteria, leaving open the question of how MukBEF complex loads on the chromosome, assuming it requires a specific loader (Nolivos and Sherratt, 2014). *E. coli* MukBEF was found, by ChIP-seq, to enrich at *ter* (Nolivos et al., 2016). We also observed an enrichment of *Caulobacter* SMC near the *ter* region (Figures 3A and 3B) and a slight increase in inter-focus distance in *Δsmc* cells when intra-arm loci at +1,600 kb and +1,800 kb were labeled (Figure 3E). The enrichment of *Caulobacter* SMC near *ter* might occur via a *parS*-independent *E.-coli*-like mechanism; however, it does not result in DNA alignment in this area (Figure 1B). The mechanism of SMC enrichment at *ter* is currently unknown in *Caulobacter.*

Evidence that Bacterial SMC Tethers Chromosomal Arms Together

The *Caulobacter* SMC complex promotes interactions between loci at approximately equivalent positions on opposite arms of the chromosome up to at least 600 kb from *parS*. It could be that SMC physically tethers the arms together. Alternatively, SMC could promote alignment passively by compacting each arm separately, reducing the cytoplasmic mobility of each arm and thereby increasing inter-arm interactions. The Hi-C patterns documented here for the WT and various inversion strains are most easily explained by the active alignment model, in which SMC physically links DNA from both arms together (Figure 7). Such a model is also appealing given the notion that SMC can topologically entrap DNA. Moreover, contact probability curves derived from the Hi-C data, which reflect global chromosome compaction, were generally very similar for *Δsmc* and WT cells (Le et al., 2013), suggesting that SMC plays only a minor role in intra-arm compaction in *Caulobacter*. Nevertheless, we cannot completely rule out other possible ParB-independent roles of SMC on chromosomal arm compaction. In the active alignment model, also suggested by studies of *B. subtilis* SMC (Wang et al., 2017), the inter-arm interactions documented by Hi-C may reflect loop generation by SMC (Figure 7). In WT cells, DNA from each chromosomal arm may be effectively threaded through SMC at approximately similar rates as SMC moves toward *ter* and away from *parS* (Figures 7A and 7B). In the inversion strains, DNA on the left arm may be threaded through SMC less efficiently than the right arm due to conflicts with convergent transcription (Figure 7C). As suggested for eukaryotic SMC, loop enlargement may be a general mechanism for folding chromosomes or bringing distant loci together (Fudenberg et al., 2016; Nasmyth, 2001).

(B) Normalized Hi-C contact maps for non-flip, Flip 3-4, Flip 2-4, and Flip 4-5 cells. The black dashed line indicates the secondary diagonal of the square matrix. A 1,000-kb region surrounding *parS/ori* was zoomed in and presented below each whole-genome Hi-C contact map.

(C) Hi-C interaction scores along the secondary diagonal for contact maps of non-flip (black), Flip 3-4 (red), Flip 2-4 (blue), and Flip 4-5 cells (dark green). Vertical black dashed lines show position where Hi-C interaction scores start to reduce in the Flip strains compared to the non-flip strain.

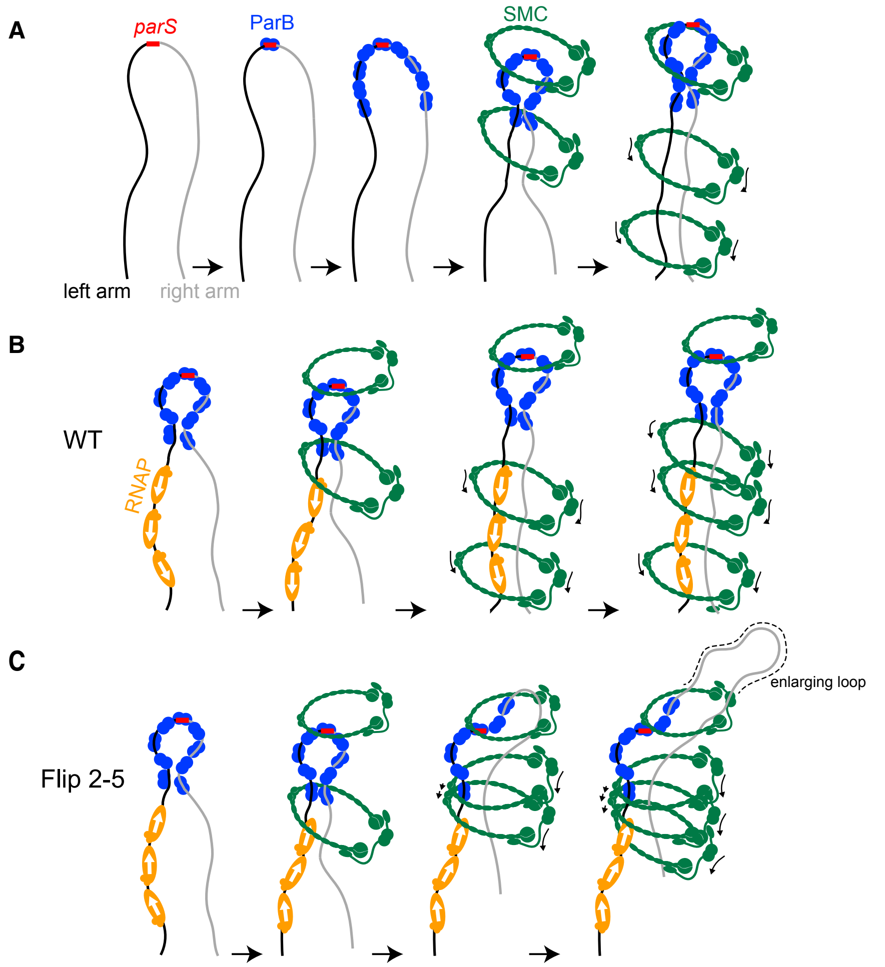

Figure 7. A Schematic Model for an Active Alignment of the Left and Right Chromosomal Arms by SMC

(A) ParB (blue) binds to the bacterial centromere *parS* site (red), spreads, and might bridge the left (black) and the right (gray) chromosomal arms together. SMC (dark green) is recruited by ParB and most likely tethers the two arms of the chromosome together. An SMC-ScpA-ScpB complex can either hold both chromosome arms within its lumen or two SMC complexes, each encircles one chromosome arm can handcuff to tether both arms together. For simplicity, only SMC encircling both arms is shown schematically.

(B) A schematic model of how SMC loads and translocates on the chromosome.

(C) A schematic model of how a high density of converging RNA polymerases (orange) might interact physically with SMC or create unfavorable DNA supercoiling that stalls or dissociates SMC from the left chromosomal arm as can happen in the Flip 2-5 strain. Schematic pictures are not to scale.

Caulobacter SMC Is Not Uniformly Distributed across the Genome

Caulobacter SMC may translocate only ∼600 kb toward *ter* whereas *B. subtilis* SMC loaded at *parS* translocates the full length of the chromosome to the terminus. It is possible that *Caulobacter* SMC may suffer more frequent conflict with convergent transcribed genes (see direction bias ratio; Figure S6A) or be more sensitive to dissociation following such conflicts, leading to less extensive arm-arm interaction. The extent to which the two arms "zip up" may not matter. If the primary role of SMC-mediated arm-arm interactions is to help enforce the individualization of sister chromosomes immediately after DNA replication, it may only be necessary to ensure that SMC can cohese *parS*-proximal regions of each chromosome. Indeed, in *Caulobacter*, ParA-ParB-*parS* are only required for the segregation of *ori*-proximal DNA, but not of the distal DNA loci (Badrinarayanan et al., 2015b). Once the *ori*-proximal DNA is properly segregated, by SMC and ParA-ParB-*parS*, distal DNA regions follow suit, driven by separate molecular machinery or more likely without the need of a dedicated system (Badrinarayanan et al., 2015b). In such a case, it may be sufficient to have SMC tether together only a limited region of DNA flanking the *parS* sites. This model would imply that conflicts between SMC and highly expressed genes oriented toward *ori* are most detrimental, with respect to chromosome segregation, if they occur in close proximity to *parS*. Testing this model and further understanding the relationship of SMC and gene expression and its influence on chromosome organization is an important challenge for the future.

EXPERIMENTAL PROCEDURES

Strains, Media, and Growth Conditions

E. coli and *C. crescentus* were grown in LB and PYE, respectively. When appropriate, media were supplemented with antibiotics at the following concentrations (liquid/solid media for *C. crescentus*; liquid/solid media for *E. coli* [μg/mL]): chloramphenicol (1/2; 20/30); kanamycin (5/25; 30/50); spectinomycin (25/100; 50/50); oxytetracycline (1/2; 12/12); and gentamycin (0.5/5; 15/20). Synchronizations of *C. crescentus* were performed on mid-exponential phase cells using Percoll (Sigma) and density gradient centrifugation. After synchronization, swarmer cells were released into PYE with 1% formaldehyde for fixation for Hi-C and ChIP-seq. All strains used in this study are listed in Table S1. All plasmids and primers used in strain and plasmid construction are listed in Table S2.

Chromosome Conformation Capture with Deep Sequencing (Hi-C) and Generation of Contacts Maps

After synchronization, swarmer cells were released into PYE + 1% formaldehyde to fix for Hi-C. Hi-C experiments were performed exactly as described previously (Le et al., 2013; Supplemental Information). After sequencing, each end of paired-end sequencing reads was mapped independently to the genome of *Caulobacter* using Bowtie 2.1.0 (Imakaev et al., 2012). The *Caulobacter* genome was then divided into 700 BglII restriction fragments. Each read of a read pair was sorted into its corresponding restriction fragment. Read pairs were classified as valid Hi-C, non-ligation, or self-ligation products (Imakaev et al., 2012). Only valid Hi-C products that are uniquely aligned to the *Caulobacter* genome were employed to generate Hi-C contact maps. To create interaction matrices, we assigned valid Hi-C products to the 10-kb bins along the genome and normalized using an iterative correction procedure as described previously (Imakaev et al., 2012; Le et al., 2013). Subsequent analysis and visualization of the Hi-C maps was done using R scripts. For *Caulobacter*

strains with an inverted DNA segment, a reconstructed fasta file with the correct orientation for the inverted segment was used as reference genome instead.

ChIP-Seq

For ChIP-seq of FLAG-tagged SMC, mid-exponential phase *Caulobacter* cultures were washed twice in 1× PBS buffer before addition of formaldehyde and crosslinker Gold (Diagenode) to a final concentration of 1%. Fixed cells were incubated at room temperature for 30 min and then quenched with 0.125 M glycine for 15 min at room temperature. For more details, see the Supplemental Information.

To analyze ChIP-seq data, Hiseq 2500 reads were mapped to the *Caulobacter* genome using Bowtie as follows:

*bowtie -m 1 -n 1–best–strata -p 4–chunkmbs 512 NA1000-2014-bowtie–sam *.fastq > output.sam.*

For *Caulobacter* strains with an inverted DNA segment, a reconstructed fasta file with the correct orientation for the inverted segment was used as reference genome instead. Subsequently, sequencing reads were allocated to their corresponding 1-kb bins along the chromosome and were normalized for the total number of reads to give the RPKPM value. Finally, the profiles of SMC enrichment were plotted with the x axis, representing genomic positions, and the y axis is RPKPM from the ChIP fraction of FLAG-tagged SMC divided by RPKPM from the ChIP fraction of untagged SMC.

Statistical Methods

A paired Student's t test was performed to test whether Hi-C interaction scores along the secondary diagonal of ParA^{K20R}-YFP-overexpression minus that of ParA^{WT}-YFP-overexpression is significantly different from 0, i.e., whether ParA^{K20R}-YFP shows reduced arm alignment to ParA^{WT}-YFP (Figures 2D and 2E).

The R program was used to compute the Pearson correlation coefficient between ChIP-seq data and Hi-C interactions along the secondary diagonal for WT and Flip 2-5 strains.

A one-tailed Student's t test (implemented in R) was performed to test whether the inter-focus distance in *Δsmc* is greater than in WT cells (Figure 3E). For the number of cells used in microscopy analysis, see the Supplemental Information.

ACCESSION NUMBERS

The accession number for the sequencing data reported in this paper is GEO: GSE97330.

SUPPLEMENTAL INFORMATION

Supplemental Information includes Supplemental Experimental Procedures, seven figures, and three tables and can be found with this article online at http://dx.doi.org/10.1016/j.celrep.2017.08.026.

AUTHOR CONTRIBUTIONS

Conceptualization, N.T.T., M.T.L., and T.B.K.L.; Data Analysis, N.T.T., M.T.L., and T.B.K.L.; Writing, N.T.T., M.T.L., and T.B.K.L.; Funding Acquisition, T.B.K.L.

ACKNOWLEDGMENTS

We thank Anjana Badrinarayanan, Hugo Brandao, Matt Bush, and Monica Guo for discussion and comments on the manuscript. We thank Lucy Shapiro, Martin Thanbichler, and Susan Schlimpert for materials. This study was supported by a Royal Society University Research Fellowship (UF140053 and RG150448) and a BBSRC grant (BB/P018165/1) to T.B.K.L. M.T.L. is an Investigator of the Howard Hughes Medical Institute.

Received: March 31, 2017
Revised: July 7, 2017
Accepted: August 3, 2017
Published: August 29, 2017

REFERENCES

Badrinarayanan, A., Reyes-Lamothe, R., Uphoff, S., Leake, M.C., and Sherratt, D.J. (2012). In vivo architecture and action of bacterial structural maintenance of chromosome proteins. Science *338*, 528–531.

Badrinarayanan, A., Le, T.B.K., and Laub, M.T. (2015a). Bacterial chromosome organization and segregation. Annu. Rev. Cell Dev. Biol. *31*, 171–199.

Badrinarayanan, A., Le, T.B.K., and Laub, M.T. (2015b). Rapid pairing and resegregation of distant homologous loci enables double-strand break repair in bacteria. J. Cell Biol. *210*, 385–400.

Breier, A.M., and Grossman, A.D. (2007). Whole-genome analysis of the chromosome partitioning and sporulation protein Spo0J (ParB) reveals spreading and origin-distal sites on the Bacillus subtilis chromosome. Mol. Microbiol. *64*, 703–718.

Bürmann, F., Shin, H.-C., Basquin, J., Soh, Y.-M., Giménez-Oya, V., Kim, Y.-G., Oh, B.-H., and Gruber, S. (2013). An asymmetric SMC-kleisin bridge in prokaryotic condensin. Nat. Struct. Mol. Biol. *20*, 371–379.

Cuylen, S., Metz, J., and Haering, C.H. (2011). Condensin structures chromosomal DNA through topological links. Nat. Struct. Mol. Biol. *18*, 894–901.

D'Ambrosio, C., Schmidt, C.K., Katou, Y., Kelly, G., Itoh, T., Shirahige, K., and Uhlmann, F. (2008). Identification of cis-acting sites for condensin loading onto budding yeast chromosomes. Genes Dev. *22*, 2215–2227.

Figge, R.M., Easter, J., and Gober, J.W. (2003). Productive interaction between the chromosome partitioning proteins, ParA and ParB, is required for the progression of the cell cycle in Caulobacter crescentus. Mol. Microbiol. *47*, 1225–1237.

Fudenberg, G., Imakaev, M., Lu, C., Goloborodko, A., Abdennur, N., and Mirny, L.A. (2016). Formation of chromosomal domains by loop extrusion. Cell Rep. *15*, 2038–2049.

Graham, T.G.W., Wang, X., Song, D., Etson, C.M., van Oijen, A.M., Rudner, D.Z., and Loparo, J.J. (2014). ParB spreading requires DNA bridging. Genes Dev. *28*, 1228–1238.

Gruber, S., and Errington, J. (2009). Recruitment of condensin to replication origin regions by ParB/SpoOJ promotes chromosome segregation in B. subtilis. Cell *137*, 685–696.

Imakaev, M., Fudenberg, G., McCord, R.P., Naumova, N., Goloborodko, A., Lajoie, B.R., Dekker, J., and Mirny, L.A. (2012). Iterative correction of Hi-C data reveals hallmarks of chromosome organization. Nat. Methods *9*, 999–1003.

Ivanov, D., and Nasmyth, K. (2005). A topological interaction between cohesin rings and a circular minichromosome. Cell *122*, 849–860.

Jensen, R.B., and Shapiro, L. (1999). The Caulobacter crescentus smc gene is required for cell cycle progression and chromosome segregation. Proc. Natl. Acad. Sci. USA *96*, 10661–10666.

Johzuka, K., and Horiuchi, T. (2007). RNA polymerase I transcription obstructs condensin association with 35S rRNA coding regions and can cause contraction of long repeat in Saccharomyces cerevisiae. Genes Cells *12*, 759–771.

Le, T.B., and Laub, M.T. (2016). Transcription rate and transcript length drive formation of chromosomal interaction domain boundaries. EMBO J. *35*, 1582–1595.

Le, T.B., Imakaev, M.V., Mirny, L.A., and Laub, M.T. (2013). High-resolution mapping of the spatial organization of a bacterial chromosome. Science *342*, 731–734.

Lieberman-Aiden, E., van Berkum, N.L., Williams, L., Imakaev, M., Ragoczy, T., Telling, A., Amit, I., Lajoie, B.R., Sabo, P.J., Dorschner, M.O., et al. (2009). Comprehensive mapping of long-range interactions reveals folding principles of the human genome. Science *326*, 289–293.

Lin, D.C.-H., and Grossman, A.D. (1998). Identification and characterization of a bacterial chromosome partitioning site. Cell *92*, 675–685.

Marbouty, M., Le Gall, A., Cattoni, D.I., Cournac, A., Koh, A., Fiche, J.-B., Mozziconacci, J., Murray, H., Koszul, R., and Nollmann, M. (2015). Condensin- and replication-mediated bacterial chromosome folding and origin condensation revealed by Hi-C and super-resolution imaging. Mol. Cell *59*, 588–602.

Mascarenhas, J., Soppa, J., Strunnikov, A.V., and Graumann, P.L. (2002). Cell cycle-dependent localization of two novel prokaryotic chromosome segregation and condensation proteins in Bacillus subtilis that interact with SMC protein. EMBO J. *21*, 3108–3118.

Minnen, A., Attaiech, L., Thon, M., Gruber, S., and Veening, J.-W. (2011). SMC is recruited to oriC by ParB and promotes chromosome segregation in Streptococcus pneumoniae. Mol. Microbiol. *81*, 676–688.

Minnen, A., Bürmann, F., Wilhelm, L., Anchimiuk, A., Diebold-Durand, M.-L., and Gruber, S. (2016). Control of Smc coiled coil architecture by the ATPase heads facilitates targeting to chromosomal ParB/parS and release onto flanking DNA. Cell Rep. *14*, 2003–2016.

Mohl, D.A., and Gober, J.W. (1997). Cell cycle-dependent polar localization of chromosome partitioning proteins in Caulobacter crescentus. Cell *88*, 675–684.

Murray, H., and Errington, J. (2008). Dynamic control of the DNA replication initiation protein DnaA by Soj/ParA. Cell *135*, 74–84.

Nasmyth, K. (2001). Disseminating the genome: joining, resolving, and separating sister chromatids during mitosis and meiosis. Annu. Rev. Genet. *35*, 673–745.

Nolivos, S., and Sherratt, D. (2014). The bacterial chromosome: architecture and action of bacterial SMC and SMC-like complexes. FEMS Microbiol. Rev. *38*, 380–392.

Nolivos, S., Upton, A.L., Badrinarayanan, A., Müller, J., Zawadzka, K., Wiktor, J., Gill, A., Arciszewska, L., Nicolas, E., and Sherratt, D. (2016). MatP regulates the coordinated action of topoisomerase IV and MukBEF in chromosome segregation. Nat. Commun. *7*, 10466.

Ocampo-Hafalla, M., Muñoz, S., Samora, C.P., and Uhlmann, F. (2016). Evidence for cohesin sliding along budding yeast chromosomes. Open Biol. *6*, 150178.

Rocha, E.P.C. (2008). The organization of the bacterial genome. Annu. Rev. Genet. *42*, 211–233.

Schwartz, M.A., and Shapiro, L. (2011). An SMC ATPase mutant disrupts chromosome segregation in Caulobacter. Mol. Microbiol. *82*, 1359–1374.

Shebelut, C.W., Guberman, J.M., van Teeffelen, S., Yakhnina, A.A., and Gitai, Z. (2010). Caulobacter chromosome segregation is an ordered multistep process. Proc. Natl. Acad. Sci. USA *107*, 14194–14198.

Stigler, J., Çamdere, G.Ö., Koshland, D.E., and Greene, E.C. (2016). Single-molecule imaging reveals a collapsed conformational state for DNA-bound cohesin. Cell Rep. *15*, 988–998.

Teytelman, L., Thurtle, D.M., Rine, J., and van Oudenaarden, A. (2013). Highly expressed loci are vulnerable to misleading ChIP localization of multiple unrelated proteins. Proc. Natl. Acad. Sci. USA *110*, 18602–18607.

Toro, E., Hong, S.-H., McAdams, H.H., and Shapiro, L. (2008). Caulobacter requires a dedicated mechanism to initiate chromosome segregation. Proc. Natl. Acad. Sci. USA *105*, 15435–15440.

Uhlmann, F. (2016). SMC complexes: from DNA to chromosomes. Nat. Rev. Mol. Cell Biol. *17*, 399–412.

Umbarger, M.A., Toro, E., Wright, M.A., Porreca, G.J., Baù, D., Hong, S.H., Fero, M.J., Zhu, L.J., Marti-Renom, M.A., McAdams, H.H., et al. (2011). The three-dimensional architecture of a bacterial genome and its alteration by genetic perturbation. Mol. Cell *44*, 252–264.

Viollier, P.H., Thanbichler, M., McGrath, P.T., West, L., Meewan, M., McAdams, H.H., and Shapiro, L. (2004). Rapid and sequential movement of individual chromosomal loci to specific subcellular locations during bacterial DNA replication. Proc. Natl. Acad. Sci. USA *101*, 9257–9262.

Wang, X., Le, T.B.K., Lajoie, B.R., Dekker, J., Laub, M.T., and Rudner, D.Z. (2015). Condensin promotes the juxtaposition of DNA flanking its loading site in Bacillus subtilis. Genes Dev. *29*, 1661–1675.

Wang, X., Brandão, H.B., Le, T.B.K., Laub, M.T., and Rudner, D.Z. (2017). Bacillus subtilis SMC complexes juxtapose chromosome arms as they travel from origin to terminus. Science *355*, 524–527.

Wilhelm, L., Bürmann, F., Minnen, A., Shin, H.-C., Toseland, C.P., Oh, B.-H., and Gruber, S. (2015). SMC condensin entraps chromosomal DNA by an ATP hydrolysis dependent loading mechanism in Bacillus subtilis. eLife *4*, e06659.

Cell Reports, Volume *20*

Supplemental Information

SMC Progressively Aligns Chromosomal Arms in *Caulobacter crescentus* but Is Antagonized by Convergent Transcription

Ngat T. Tran, Michael T. Laub, and Tung B.K. Le

SUPPLEMENTARY INFORMATION

SMC progressively aligns chromosomal arms in *Caulobacter crescentus* but is antagonized by convergent transcription

Ngat T. Tran, Michael T. Laub, and Tung B. K. Le

SUPPLEMENTARY FIGURES AND LEGENDS

SUPPLEMENTARY EXPERIMENTAL PROCEDURES

SUPPLEMENTARY TABLES S1-S3

SUPPLEMENTARY REFERENCES

Figure S1

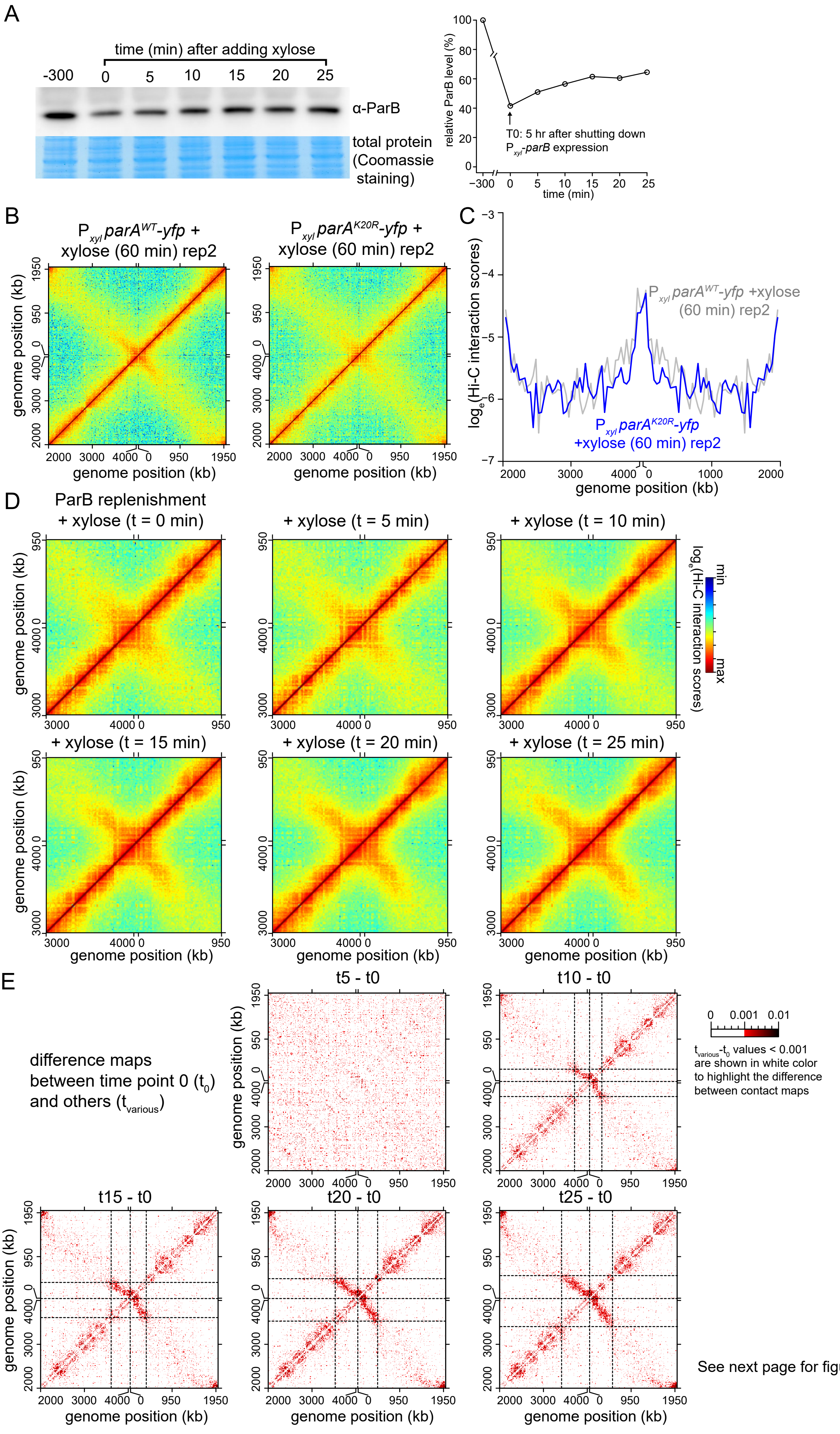

See next page for figure legend

Fig. S1. ParB is required for the progressive alignment of chromosomal arms by SMC, Related to Fig. 2.

(A) Immunoblot analysis of ParB level before the start of the depletion experiment (t = -300 min), at the end of the depletion experiment (t = 0 min), and at time point 5, 10, 15, 20 and 25 minutes after adding back xylose to replenish ParB. **(B)** Normalized Hi-C maps for cells over-expressing *parA*^{WT}*-yfp* (replicate 2) and *parA*^{K20R}*-yfp* (replicate 2) after adding xylose for 1 hr. **(C)** Hi-C interaction scores along the secondary diagonal for contact maps of cells over-expressing *parA*^{WT}*-yfp* (replicate 2) (grey), and *parA*^{K20R}*-yfp* (replicate 2) (blue) after adding xylose for 1 hr. **(D)** A time-resolved Hi-C contact maps for cells that are replenishing of ParB. *parB::*P_{xyl} *parB* cells at the end of the 5 hr depletion period was washed off glucose and supplemented with xylose to induce ParB production. Time (in minutes) after adding back xylose was indicated on top of each Hi-C map. A 1000 kb region surrounding *parS/ori* were zoomed in. **(E)** Hi-C difference maps between time point 0 and others ($t_{various}$ – t_0). The black dotted lines indicate the leading front of the aligned DNA. The rates of progression for each arm of the chromosome are shown in Fig. 2G.

Figure S2

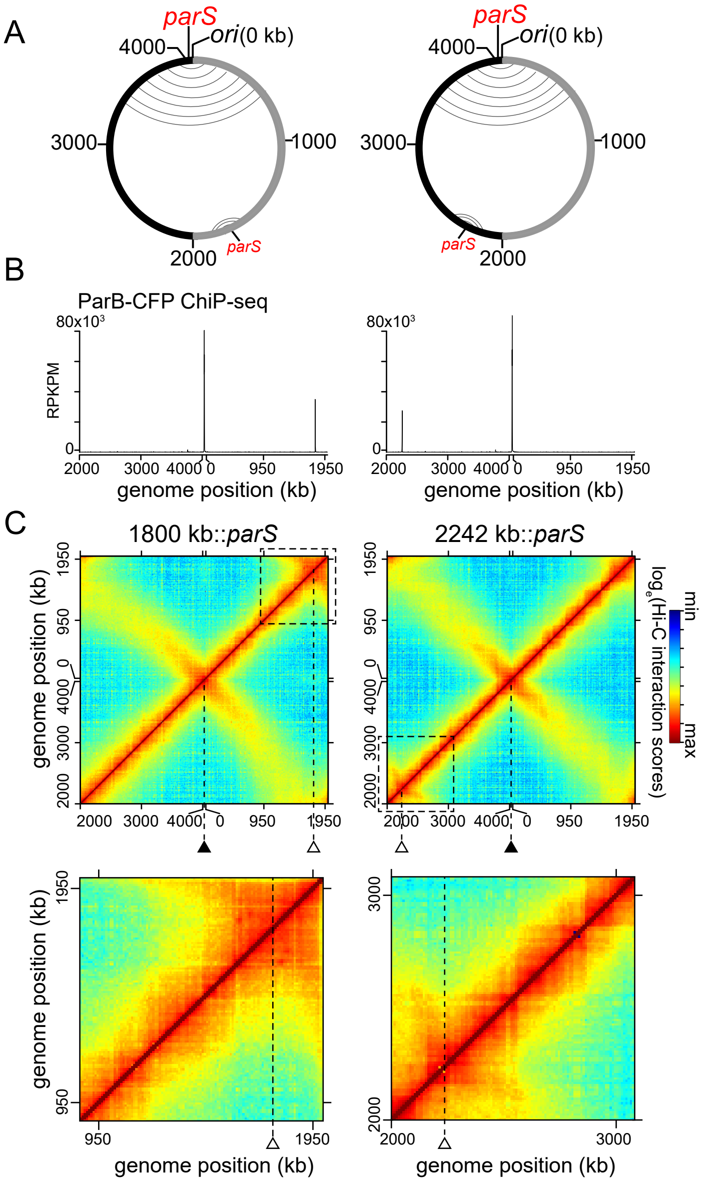

Fig. S2. An ectopic *parS* induces the alignment of its flanking DNA, Related to Fig. 3.

(A) Genomic maps show the locations of a 260 bp DNA fragment containing *parS* site that were engineered at +1800 kb and +2242 kb. Aligned DNA regions are presented as grey curved lines connecting the left and the right flanking of each *parS* site (see also panel **C**). **(B)** ChIP-seq profiles show ParB distribution in cells harbouring a second *parS* site at +1800 kb or at +2242 kb. ChIP-seq using α-GFP antibody were performed in the above cells with *parB*::*cfp-parB* as the sole source of ParB. ChIP-seq enrichment was expressed as number of reads per kilobases per million of mapped reads (RPKPM). **(C)** Normalized Hi-C maps for the +1800 kb::*parS* and +2242 kb::*parS* cells. The solid and open triangles shows the position of the native and ectopic *parS* site, respectively. A 1000 kb region surrounding the ectopic *parS* site (black dashed box) were also zoomed in and presented below each whole-genome Hi-C contact map.

Figure S3

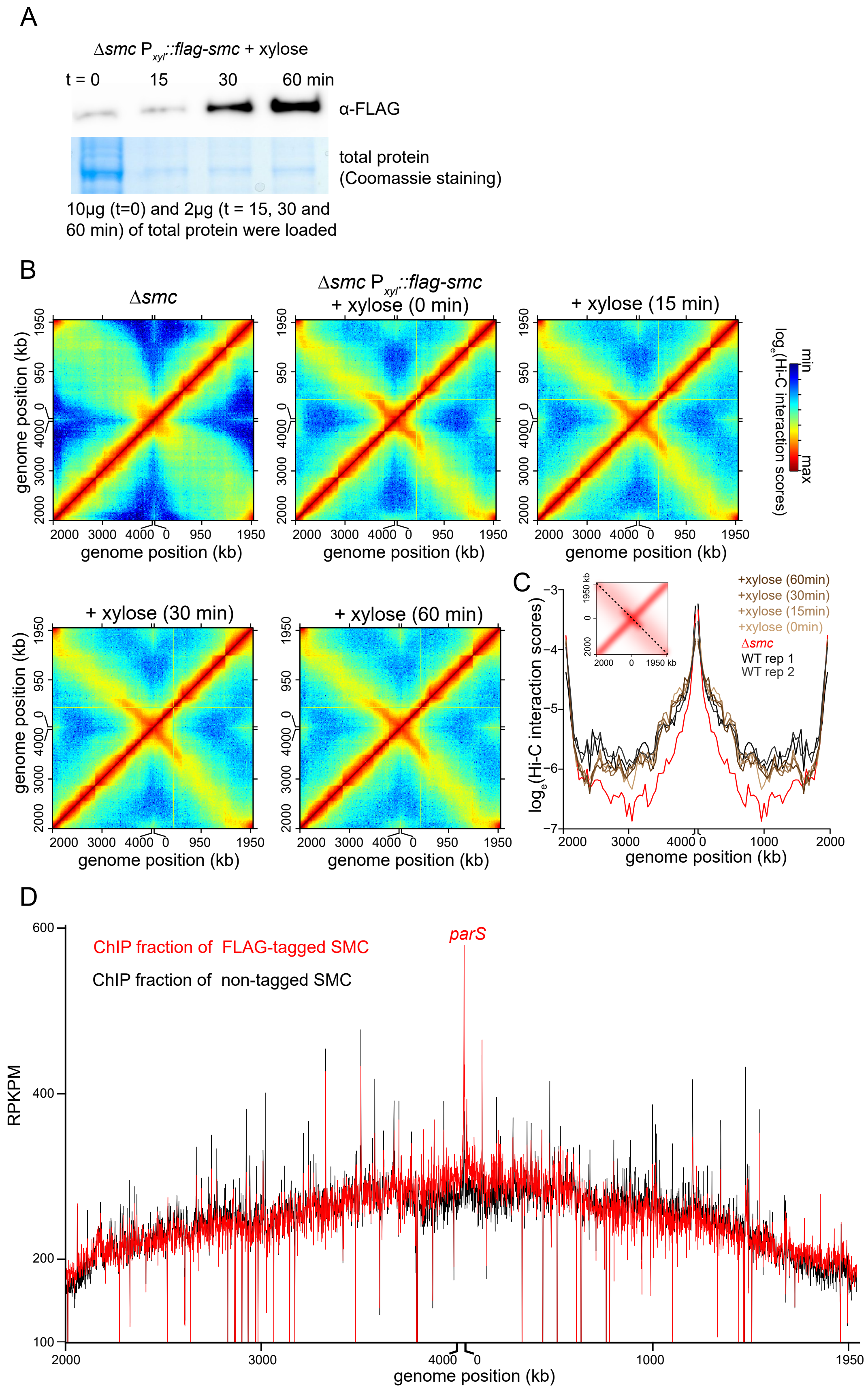

See next page for figure legend

Fig. S3. A FLAG-tagged SMC restores chromosomal arm alignment to *Δsmc Caulobacter*, Related to Fig. 3.

(A) Immunoblot analysis of SMC level at time point 0 (leaky expression), 15, 30, and 60 minutes after adding xylose. For time point t = 0, 10 µg total protein is loaded. For time point t = 15, 30 and 60 min, only 2 µg total protein is loaded to avoid oversaturating the signal on an immunoblot. **(B)** Normalized Hi-C contact maps showing the natural logarithm of DNA-DNA contacts for pairs of 10kb-bins across the genome for *Δsmc* cells (Le et al., 2013), and *Δsmc* P_{xyl}*::flag-smc* after adding xylose for 0, 15, 30 and 60 min. A leaky expression of P_{xyl} (0 min, no added glucose) is sufficient to restore the chromosomal arm alignment. **(C)** Hi-C interaction scores along the secondary diagonal for contact maps of WT, *Δsmc* and for *Δsmc* P_{xyl}*::flag-smc* after adding xylose for 0, 15, 30 and 60 min. **(D)** ChIP fraction (expressed as RPKPM) of *Δsmc* P_{xyl}*::flag-smc* (red) from a ChIP-seq experiment using α-FLAG antibody. ChIP fraction of a non-tagged SMC strain (black) from a ChIP-seq experiment using α-FLAG antibody. Both strains were grown to exponential phase in rich medium at 30°C before fixation with 1% formadehyde and crosslinker Gold.

Figure S4

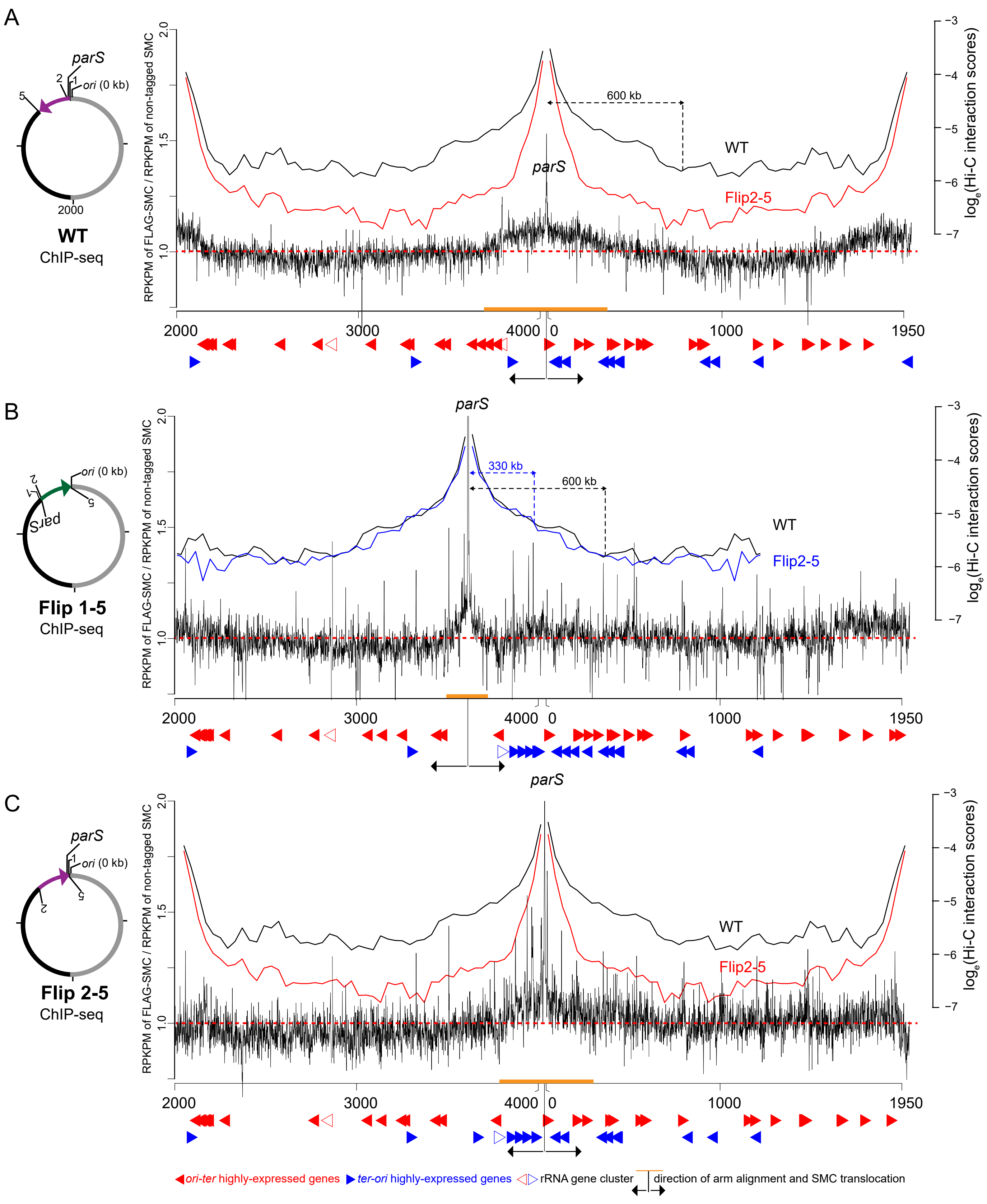

See next page for figure legend

Fig. S4. The genome-wide distribution of FLAG-tagged SMC, Related to Fig. 3 and Fig. 4.

(A) The distribution of FLAG-tagged SMC on WT *Caulobacter* chromosome. DNA from both the α-FLAG ChIP fraction of tagged-SMC and un-tagged SMC were deep sequenced. ChIP-seq signals were reported as the number of reads within every 1 kb bin along the genome in the ChIP fraction of FLAG-tagged SMC divide by that of untagged SMC. The dashed red line shows y-axis value at 1. Below the ChIP-seq profile are the position of highly-expressed genes that transcribe in the *ori-ter* (solid red arrows) or *ter-ori* (solid blue arrows) direction. The positions of rRNA gene cluster are indicated with open red or blue arrows. The direction and extent of SMC translocation from *parS* site were shown as black arrows and orange bar, respectively. High expression genes (RPKPM*gene length > 1000) were determined from α-FLAG ChIP-seq in cells expressing *rpoC-flag* (See Fig. 4A and Fig. S6). A schematic genomic map of *Caulobacter* showing the position of *parS* and *ori* are presented on the left hand side of each ChIP-seq profile. The inverted DNA segment (purple/green arrow) as in the Flip 1-5 or Flip 2-5 strains is indicated together with the end points of the inversion (1, 2, and 5). The aligned DNA regions, as observed by Hi-C, are presented as grey curved lines connecting the left and the right chromosomal arm. Overlaid on the ChIP-seq profile is Hi-C interaction scores along the diagonal from the upper left corner to the lower right corner of each Hi-C contact map for WT (black) and Flip 2-5 (red). **(B)** The distribution of FLAG-tagged SMC on the chromosome of Flip 1-5 *Caulobacter*. Overlaid on the ChIP-seq profile is Hi-C interaction scores along the diagonal from the upper left corner to the lower right corner of each Hi-C contact map for WT (black) and Flip 1-5 (blue). The Hi-C interaction scores along the diagonal for WT contact map was shifted to the same position as for Flip 1-5 map to enable comparison between strains **(C)** The distribution of FLAG-tagged SMC on the chromosome of Flip 2-5 *Caulobacter*. Overlaid on the ChIP-seq profile is Hi-C interaction scores along the diagonal from the upper left corner to the lower right corner of each Hi-C contact map for WT (black) and Flip 2-5 (red).

Figure S5

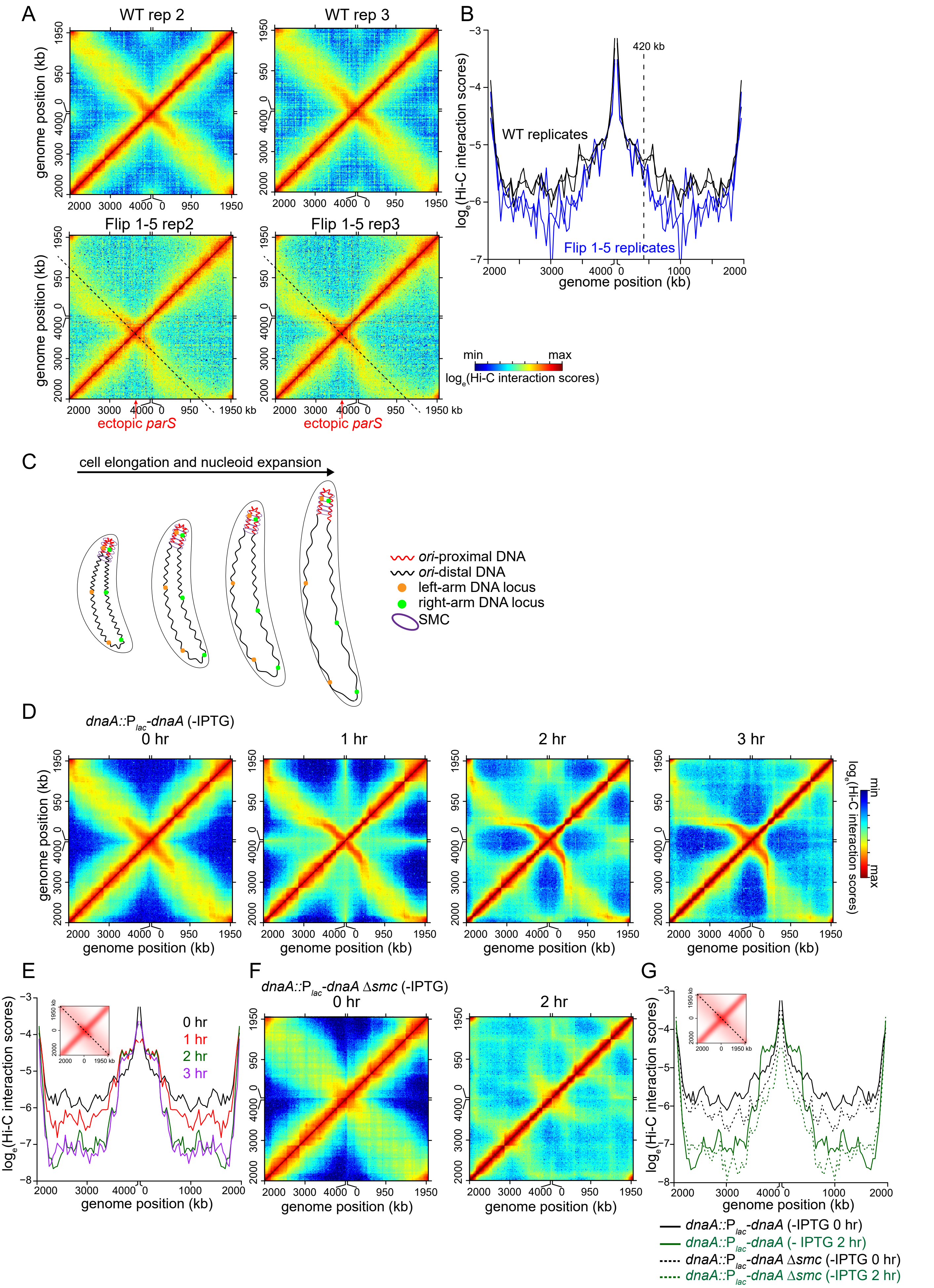

See next page for figure legend

Fig. S5. Hi-C analysis of Flip 1-5 strain and of elongated *Caulobacter* cells, Related to Fig. 3.

(A) Normalized Hi-C maps for the Flip 1-5 (replicate 2-3) and WT cells (replicate 2-3). The genomic position of the relocated *parS* site in Flip 1-5 is indicated with a red arrow. **(B)** Hi-C interaction scores along the diagonal from the upper left corner to the lower right corner for contact maps of WT (black), Flip 1-5 (blue). The Hi-C interaction scores along the secondary diagonal of Flip 1-5 (black dashed lines in panel **A**) was shifted to the same position as that of WT to enable comparison between strains. The vertical black dashed line at ~420 kb away from *ori* shows the position where Hi-C interaction scores along the secondary diagonal start to reduce in Flip 1-5 strain in comparison to WT. **(C)** A schematic presentation of cell elongation and chromosome expansion due to DnaA depletion. Cells depleted of DnaA are arrested in G1 phase but continue to grow, resulting in an elongated morphology with the nucleoid expanding to fill the larger available cytoplasmic volume (Kahng and Shapiro, 2003; Le and Laub, 2016). Pair of DNA loci (orange and green dots) were labeled fluorescently using the orthogonal ParB/*parS* system (Badrinarayanan et al., 2015). SMC, *ori*-proximal DNA and *ori*-distal DNA were labeled as purple eclipse, red and black wavy lines, respectively. **(D)** Hi-C maps for cells depleted of DnaA for the times indicated (Le and Laub, 2016). **(E)** Hi-C interaction scores along the secondary diagonal for Hi-C contact maps of cells depleted of DnaA at various time points. **(F)** Hi-C maps for *Δsmc* cells depleted of DnaA for the times indicated. **(G)** Hi-C interaction scores along the secondary diagonal for Hi-C contact maps of *Δsmc* cells depleted of DnaA at various time points.

Figure S6

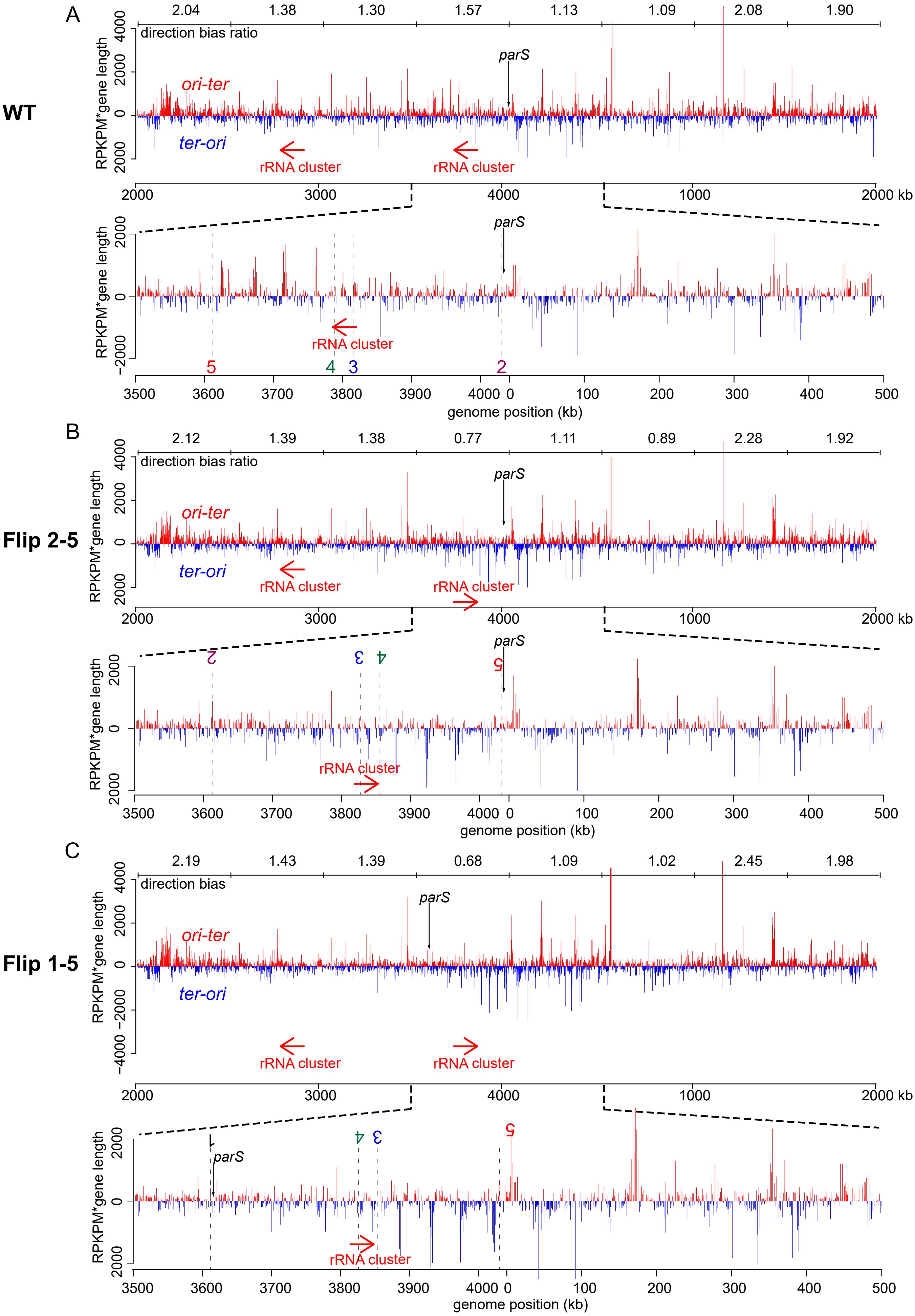

See next page for figure legend

Fig. S6. ChIP-seq of FLAG-tagged RpoC to quantify RNA polymerase abundance and the bias in direction of transcription, Related to Fig. 4.

The abundance of RNA polymerases on genes that are transcribing in the *ori-ter* direction (red) and in the *ter-ori* direction (blue) is shown for WT (Panel **A**), Flip 2-5 (Panel **B**) and Flip 1-5 cells (Panel **C**). Anti-FLAG antibody ChIP-seq was performed on exponentially-growing cells expressing RpoC-FLAG from its native locus. Only pulled-down DNA from translocating RNA polymerases were used to generate this plot. Pulled-down DNA from initiating RNA polymerases at promoter regions were discarded *in silico*. The abundance of RNA polymerases was represented as RPKPM*gene length for each gene and plotted against the genomic location of that gene. The direction bias ratio was calculated as the ratio of RPKPM*gene length for *ori-ter* genes to that of *ter-ori* genes in every 500 kb region along the *Caulobacter* genome. The direction bias ratio (greater than 1) indicates a preference for *ori-ter* transcription throughout the *Caulobacter* genome. Due to short sequencing reads (50 bp) and the high similarity between the two ribosomal RNA clusters, it is not reliable to estimate the RNA polymerase density within each rRNA cluster. Therefore, the enrichment data for rRNA clusters are not shown. Nevertheless, we indicate the genomic positions of highly-expressed rRNA clusters on *Caulobacter* genome with red arrows. A region between +3500 kb and +500 kb was further zoomed in. Vertical black dashed lines with numbering 2, 3, 4 and 5 indicate the inversion end points (See Fig. 3-4 and Fig. 6).

Figure S7

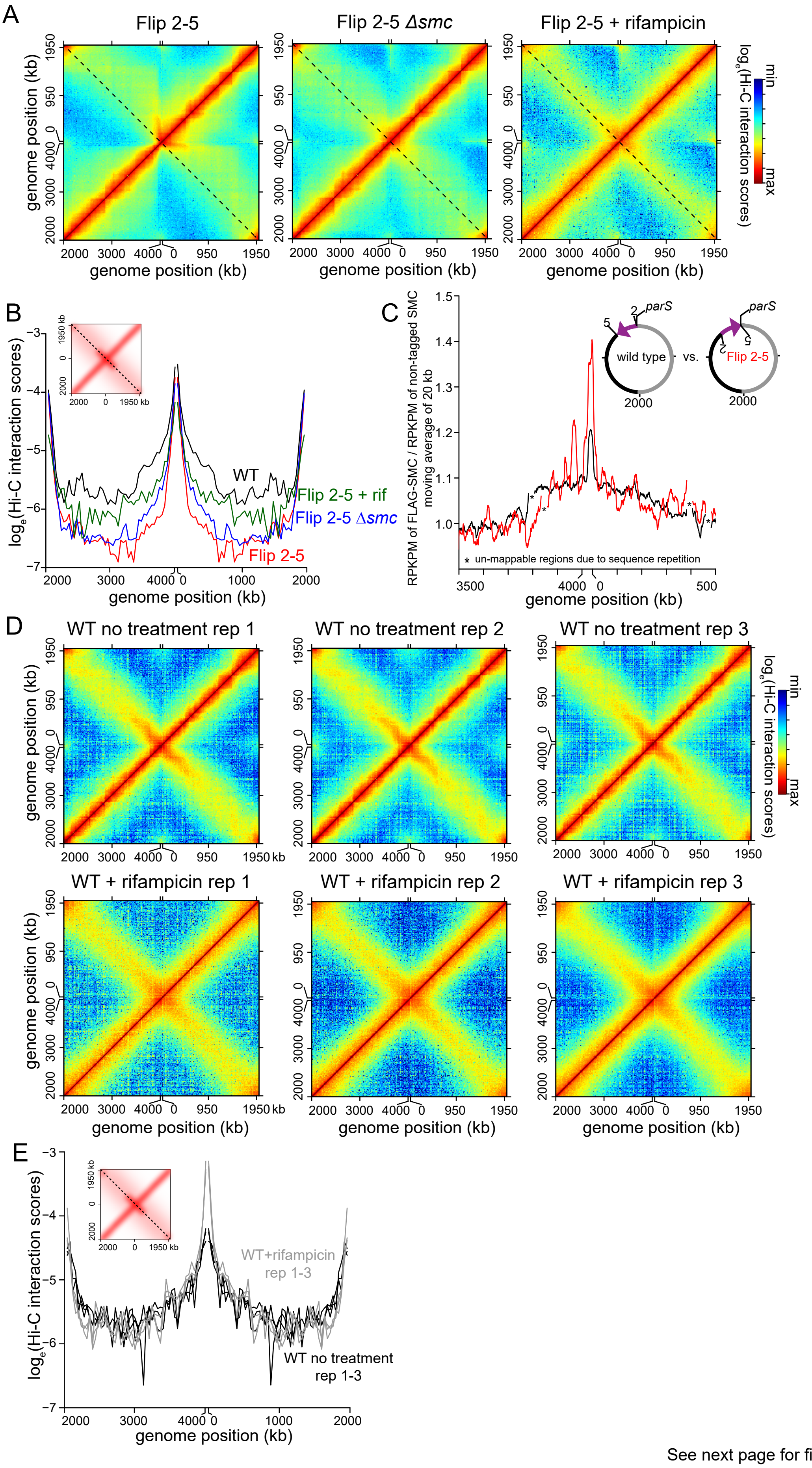

See next page for figure legend

Fig. S7. Hi-C interaction scores along the secondary diagonal for contact maps of the Flip 2-5 strains and the effect of inhibiting transcription elongation on inter-arm interactions in WT cells, Related to Fig. 4 and Fig. 5.

(A) Normalized Hi-C contact maps for Flip 2-5, Flip 2-5 *Δsmc* cells, and Flip 2-5 cells treated with rifampicin (25 µg/ml) for 30 minutes. **(B)** Hi-C interaction scores along the diagonal from the upper left corner to the lower right corner (black dashed line in panel **A**) for contact maps of WT, Flip 2-5, Flip 2-5 *Δsmc*, and Flip 2-5 cells treated with rifampicin (25 µg/ml) for 30 minutes. **(C)** The distribution of FLAG-tagged SMC on WT *Caulobacter* chromosome (black) and on Flip 2-5 chromosome (red). A moving average of twenty 1-kb bins was applied to highlight the difference between the ChIP-seq profiles of WT and Flip 2-5. See also Fig. 6A for profiles without a moving average smoothing. Only DNA segment between +3500 kb and +500 kb was shown. Asterisks (*) indicate the un-mappable DNA regions due to sequence repetition and the short-read nature of Illumina sequencing (50-bp long reads). **(D)** Normalized Hi-C contact maps showing DNA-DNA contacts for pairs of 10kb-bins across the genome of wild-type and cells treated with rifampicin (25 µg/ml) for 30 min. **(E)** Hi-C interaction scores along the secondary diagonal for Hi-C contact maps of wild-type and cells treated with rifampicin (25 µg/ml) for 30 min.

SUPPLEMENTARY EXPERIMENTAL PROCEDURES

Synchronization and growth conditions

Synchronizations of *C. crescentus* were performed on mid-exponential phase cells using Percoll (Sigma) and density gradient centrifugation. Briefly, 250 mL cultures of the wild-type CB15N or its derivatives were grown in PYE at 30°C to an OD_{600} of ~0.4 and pelleted via centrifugation at 8000 *g* for 10 min. Cells were resuspended in 5 ml of 1x M2 salts (6.1 mM Na_2HPO_4, 3.9 mM KH_2PO_4, 9.3 mM NH_4Cl, 0.5 mM $MgSO_4$, 10 µM $FeSO_4$, 0.5 mM $CaCl_2$) via pipetting and 5 mL of ice-cold Percoll was added to the resuspension. The resulting mixture was transferred to a 15 mL Falcon tube that was subsequently centrifuged for 20 min (10000 *g*) at 4°C. G1-phase swarmer cells formed a discrete band near the bottom of the tube. This band was removed via pipetting and the cells within this band were washed three times with 1 ml of ice-cold 1x M2 salts.

After synchronization, swarmer cells were released into PYE+1% formadehyde for fixation for chromosome conformation capture assays (Hi-C).

For antibiotic treatment, swarmer cells were incubated with rifampicin at 25 µg/mL final concentration for 30 minutes before fixing with 1% formadehyde for Hi-C assay.

For xylose induction experiments, xylose was added to mid-exponential phase cultures to 0.3% final concentration. Cultures were incubated with shaking for an additional hour before formadehyde was added to 1% final concentration to fix cells for Hi-C assay.

For the ParB-depletion experiment, MT148 (*parB::*P_{xyl}-*parB*) cells (Thanbichler and Shapiro, 2006) at mid-exponential phase were washed off xylose by repeated centrifugation and resuspension in fresh PYE before releasing to PYE+0.2% glucose. The culture was left shaking at 30°C for five more hours before formadehyde was added to 1% final concentration to fix cells for Hi-C assay.

For the ParB-replenishment experiment, MT148 (*parB::*P_{xyl}-*parB*) cells (Thanbichler and Shapiro, 2006) were first depleted of ParB using the above procedure. At the end of the five-hour period, cells were washed twice in fresh PYE to remove glucose. Xylose (0.3% final concentration) was then added (time point 0). Subsequently, cultures were withdrawn at regular time points (5, 10, 15, 20, and 25 minutes after the addition of xylose) and immediately fixed with 1% formadehyde for Hi-C assays.

Plasmids and Strains construction

All strains used are listed in Supplementary Table S1. All plasmids and primers used in strain and plasmid construction are listed in Supplementary Table S2.

pMCS2-ΔscpA

A 500 bp N-terminal sequence of *scpA* (CCNA_02084) was amplified by PCR using primers scpA_500N_F and scpA_500N-R and purified *Caulobacter* genomic DNA as template. The PCR product was gel-purified and assembled to an NdeI-NheI-cut pMCS2 (Thanbichler et al., 2007) using a 2x Gibson master mix (NEB). Briefly, 2.5 µL of each DNA fragment at equimolar concentration was added to 5 µL Gibson master mix (NEB), and the mixture was incubated at 50°C for 60 minutes. 5 µL was used to transform chemically-competent *E. coli* DH5α cells. Gibson assembly was possible due to a 23 bp sequence shared between the PCR fragment and the NdeI-NheI-cut pMCS2 backbone. The resulting plasmid was sequence verified by Sanger sequencing (Eurofins, Germany).

Electro-competent *Caulobacter* cells were electroporated with pMCS2-*ΔscpA* plasmid to allow for a single integration at *scpA*, thereby inactivating this gene. The correct integration of pMCS2-*ΔscpA* was verified by PCR using primers outside of the homologous regions.

pNPTS138-ΔscpB

A 500 bp upstream of the ScpB-encoding gene (CCNA_02083) that includes the first 36 nucleotides of *scpB* was amplified by PCR using primers scpBfrag1_F and scpBfrag1-R. A 500 bp downstream of the *scpB* including the last 36 nucleotides of *scpB* was amplified by PCR using primers scpBfrag2_F and scpBfrag2_R. PCR products were gel purified and assembled together with a BamHI-EcoRI-cut pNPTS138 using Gibson assembly technique. Briefly, 1.5 µL of each DNA fragment at equimolar concentration was added to 5 µL 2x Gibson master mix (NEB), and the mixture was incubated at 50°C for 60 minutes. 5 µL was used to transform chemically-competent *E. coli* DH5α cells. Gibson assembly was possible due to a 23 bp sequence shared between the three PCR fragments. This homology was incorporated during the primer design to amplify the upstream and downstream region of *scpB*. The resulting plasmid was sequence verified by Sanger sequencing (Eurofins, Germany).

For the deletion of *scpB*, pNPTS138-*ΔscpB* plasmid was first transformed to *Caulobacter* CB15N competent cells via electroporation. A double crossover gene knock-out was performed as described previously (Skerker et al., 2005). The deletion was verified using PCR with primers outside of the homologous flanking regions.

pENTR::smc

The SMC (CCNA_00377) encoding sequence was amplified by PCR from *Caulobacter* genomic DNA using primers smc_pentr_topo_F and smc_pentr_topo_R. The PCR product was gel purified before being ligated to the pENTR backbone in a reaction consisting of 2 µL of gel-purified PCR product, 0.5 µL of D-TOPO reaction buffer, and 0.5 µL of pENTR-D-TOPO vector (Invitrogen) and 2 µL of water. The reaction was incubated for an hour at room temperature before transformation into TOP10 *E. coli* cells (Invitrogen). The resulting plasmid was verified by Sanger dye-termination sequencing (Genewiz, USA).

pML477-smc

The *smc* genes were recombined into a Gateway-compatible destination vector pML477 via LR recombination reaction (Invitrogen). For LR recombination reactions: 1 µL of purified pENTR harbouring *smc* was incubated with 1 µL of the destination vector pML477, 1 µL of LR Clonase II mastermix, and 2 µL of water in a total volume of 5 µL. The reaction was incubated for an hour at room temperature before transformation into DH5α *E. coli* cells. Cells were then plated out on LB agar + spectinomycin. Resulting colonies were restruck onto LB agar + spectinomycin and LB agar + kanamycin. Only colonies that survived on LB + spectinomycin plates were subsequently used for culturing and plasmid extraction.

pNPTS138::inversion insertion point 1 to 6

These plasmids were used to insert a ΦC31 attachment site (*attP* or *attB*) at the intended locations (insertion points) on the *Caulobacter* chromosome. Insertion points are at the end of converging genes so that inverting a chromosome segment is less likely to disrupt transcription promoters near both ends of the inversion. DNA fragments were chemically-synthesized by BioBasics (Canada) to contain: 500 bp on the left hand side of the insertion point + *attP* or *attB* sequence + 500 bp on the right hand side of the insertion point. The sequence of *attP* (50bp, forward direction) is

gtagtgccccaactggggtaacctttgagttctctcagttgggggcgtag. The sequence of *attB* (40bp, reverse direction) is gtacgcgcccggggagcccaagggcacgccctggcacccg. The chemically-synthesized DNA fragments (insertion point 1, 2, 4, 5) were liberated from its carrying plasmid (pUC57, BioBasics) by a double digestion with HindIII and BamHI restriction enzymes. The dropped-out insert was gel-purified before being ligated to a HindIII-BamHI-cut pNPTS138. 10 µL of the ligation reaction was used to transform *E. coli* DH5α cells. The chemically-synthesized DNA fragment (insertion point 3) was liberated from its carrying plasmid (pUC57) by digestion with HindIII and BglII instead. The dropped-out insert was gel-purified before being ligated to a HindIII-BglII cut pNPTS138 backbone. 5 µL of the ligation reaction was used to transform *E. coli* DH5α cells.

pMCS5::parS^{Caulobacter}

DNA fragment (260 bp) containing *Caulobacter parS* sites (Toro et al., 2008) was amplified by PCR using primers CCparS_gibMCS5F and CCparS_gibMCS5R, and purified *Caulobacter* genomic DNA as template. The PCR fragment was gel-purified and assembled to an EcoRI-cut pMCS5 backbone (Thanbichler et al., 2007) using a 2x Gibson master mix (NEB). Briefly, 2.5 µL of each DNA fragment at equimolar concentration was added to 5 µL Gibson master mix (NEB), and the mixture was incubated at 50°C for 60 minutes. 5 µL was used to transform chemically-competent *E. coli* DH5α cells. Gibson assembly was possible due to 23 bp sequences shared between the two PCR fragments. These 23 bp regions were incorporated during the primer design to amplify the *parS*-containing sequence. The resulting plasmid was sequence verified by Sanger sequencing (Eurofins, Germany).

pMCS5::parS^{Caulobacter} at +1800kb and pMCS5::parS^{Caulobacter} at +2242kb

For insertion of a 260 bp sequence containing *Caulobacter parS* sites at +1800 kb in the *Caulobacter* genome, primers label1800-NdeI-F and label1800-SacI-R were used to amplify a ~500 bp fragment by PCR, using *Caulobacter* genomic DNA as template. This fragment was 5’ phosphorylated by T4 PNK (NEB) before being blunt-end ligated to a SmaI-cut pUC19 (Fermentas). The resulting construct was sequence verified. The NdeI-SacI-ended fragment was then liberated from the pUC19-based plasmid by NdeI and SacI double digestion before being cloned into the same sites on *pMCS5::parS^{Caulobacter}*. The construction of *pMCS5::parS^{Caulobacter}* for insertion at +2242 kb were carried out essentially as above, except the pairs of primers used were: label2242-NdeI-F and label2242-SacI-R.

Electro-competent *Caulobacter* cells were electroporated with these plasmids to allow for a single integration at the site of interest. The correct integration was verified by PCR using a primer specific to the *parS^{Caulobacter}* site and another primer upstream of the ~500 bp homologous region used to drive integration.

pMT619::parS^{pMT1} at +200 kb, +1000 kb and +1800 kb

For insertion of *parS^{pMT1}* at +200 kb in the *Caulobacter* genome, primers label200-NdeI-F and label200-SacI-R were used to amplify a ~500 bp fragment by PCR, using *Caulobacter* genomic DNA as template. This fragment was 5’ phosphorylated by T4 PNK (NEB) before being blunt-end ligated to a SmaI-cut pUC19 (Fermentas). The resulting construct was sequence verified. The NdeI-SacI-ended fragment was then liberated from the pUC19-based plasmid by NdeI and SacI double digestion before being cloned into the same sites on pMT619::*parS^{pMT1}* (Badrinarayanan et al., 2015). The constructions of pMT619::*parS^{pMT1}* for insertion at +1000 kb, and +1800 kb were carried out essentially as above, except the pairs of primers used were: label1000-NdeI-F and label1000-SacI-R; label1800-NdeI-F and label1800-SacI-R, respectively.

Electro-competent *Caulobacter* cells were electroporated with these plasmids to allow for a single integration at the site of interest. The correct integration was verified by PCR using a primer specific to the *parS*^{pMT1} site and another primer upstream of the ~500 bp homologous region used to drive integration.

pMT632::parS^{P1} at +3842 kb, +3042 kb and +2242 kb

To insert *parS*^{P1} sites at +3842 kb, +3042 kb and +2242 kb in the *Caulobacter* genome, the same procedure as above was used, except that the pairs of primers used were: label3842-NdeI-F and label3842-SacI-R; label3042-NdeI-F and label3042-SacI-R; and label2242-NdeI-F and label2242-SacI-R respectively. Also, the NdeI-SacI-ended fragment was then liberated from the pUC19-based plasmid by NdeI and SacI double digestion before being cloned into the same sites on pMT632::*parS*^{P1} (Badrinarayanan et al., 2015).

Electro-competent *Caulobacter* cells were electroporated with these plasmids to allow for a single integration at the site of interest. The correct integration was verified by PCR using a primer within the *parS*^{P1} site and another upstream of the ~500 bp homologous region used to drive insertion.

pGADT7-parB
The ParB coding sequence was amplified by PCR from *Caulobacter* genomic DNA using primers parB_pGAD424_F and parB_pGAD424_R. The PCR product was purified and assembled to BamHI-EcoRI-cut pGAD424 (Clonetech) using 2x Gibson mastermix. Briefly, 2.5 µL of the PCR product and the BamHI-EcoRI-cut pGAD424 at equimolar concentration was added to 5 µL Gibson master mix (NEB), and the mixture was incubated at 50°C for 60 minutes. 5 µL was used to transform chemically-competent *E. coli* DH5α cells. The resulting plasmid was sequence verified by Sanger sequencing (Eurofins, Germany). The *parB* insert was subsequently dropped out by digesting pGAD424-parB with EcoRI and BamHI, and ligated to EcoRI-BamHI-cut pGADT7 using T4 ligase (NEB). pGADT7 was used for yeast-two hybrid assay since *parB* is driven by a stronger promoter than that of pGAD424.

pGBKT7-parA WT and pGBKT7-parAK20R

The ParA coding sequence was amplified by PCR from *Caulobacter* genomic DNA using primers parA_pGBT_F and parA_pGBT_R. The PCR product was purified and assembled to BamHI-EcoRI-cut pGBT9 (Clonetech) using 2x Gibson master mix. Briefly, 2.5 µL of the PCR product and the BamHI-EcoRI-cut pGBT9 at equimolar concentration was added to 5 µL Gibson master mix (NEB), and the mixture was incubated at 50°C for 60 minutes. 5 µL was used to transform chemically-competent *E. coli* DH5α cells. The resulting plasmid was sequence verified by Sanger sequencing (Eurofins, Germany). The *parA* insert was subsequently dropped out by digesting pGBT9-parA with EcoRI and BamHI, and ligated to EcoRI-BamHI-cut pGBKT7 using T4 ligase (NEB). pGBKT7 was used for yeast two hybrid assay since *parA* is driven by a stronger promoter than that in pGBT9. The *parA (K20R)* was amplified by PCR using the exact same primers as for ParA^{WT} but from a plasmid carrying P_{xyl}-ParA^{K20R}

Generation of Caulobacter strains with knock-in attB/attP attachment site:

For the insertion of *attP/attB* attachment site on the chromosome, pNPTS138::inversion was first introduced to *Caulobacter* CB15N via electroporation. A homology-mediated double crossover was performed as described previously to knock in the *attP/attB* attachment site (Skerker et al., 2005). The insertion was verified using PCR with primers located 50 bp away on the left and on the right hand side of *attP/attB*.

To generate the inversion between insertion point 2 (nt: 4030311) and insertion point 5 (nt: 3611123):

The *attP* site (forward direction) was inserted first at the insertion point 5 via a homolog-mediated double crossover. The resulting strain was made competent and electroporated with pNPTS138::inversion insertion point 2. Again, a homology-mediated double crossover to knock in *attB* (reverse direction) at the insertion point 2 was performed as described previously (Skerker et al., 2005). The insertion at point 2 was verified using PCR with primers located 50 bp away on the left and on the right hand side of *attB*. The resulting strain was again made electroporation competent, transformed with pTORO78 that has ΦC31 integrase under the control of a vanillate-inducible promoter (Toro et al., 2008), and selected for on PYE + chloramphenicol plates. Colonies that formed on PYE + chloramphenicol were restruck on fresh PYE + chloramphenicol twice. PCR amplification was used to verify that an inversion has occurred. A leaky expression of ΦC31 integrase from the P_{van} promoter is sufficient to drive the *in vivo* recombination between *attP* at the insertion point 2 and *attB* at the insertion point 5.

To generate the inversion between insertion point 3 (nt: 3815825) and insertion point 5 (nt: 3611123):

The exact same procedure as above but pNPTS138::inversion insertion point 3 was used instead of pNPTS::inversion insertion point 2

To generate the inversion between insertion point 4 (nt: 3788231) and insertion point 5 (nt: 3611123):

The exact same procedure as above but pNPTS138::inversion insertion point 4 was used instead of pNPTS::inversion insertion point 3.

To generate the inversion between insertion point 1 (nt: 4038385) and insertion point 5 (nt: 3611123):

The exact same procedure as above but pNPTS138::inversion insertion point 1 was used instead of pNPTS::inversion insertion point 4

To generate the inversion between insertion point 2 (nt: 4030311) and insertion point 4 (nt: 3788231):

The *attP* site (forward direction) was inserted first at the insertion point 4 by homology-mediated double crossover. The resulting strain was made competent and electroporated with pNPTS138::inversion insertion point 2. Again, a homology-mediated double crossover to knock in *attB* (reverse direction) at the insertion point 2 was performed as described previously (Skerker et al., 2005).The insertion at point 2 was verified using PCR with primers located 50 bp away on the left and on the right hand side of *attB*. The resulting strains were again made electroporation competent, transformed with pTORO78 that has ΦC31 integrase under the control of a vanillate-inducible promoter (Toro et al., 2008), and selected for on PYE + chloramphenicol plates. Colonies that formed on PYE + chloramphenicol plate were restruck on fresh PYE + chloramphenicol twice. PCR amplification was used to verify that inversion has occurred. A leaky expression of ΦC31 integrase from the P_{van} promoter is sufficient to drive the *in vivo* recombination between *attP* at the insertion point 4 and *attB* at the insertion point 2.

To generate the inversion between insertion point 3 (nt: 3815825) and insertion point 4 (nt: 3788231):

The exact same procedure as above but pNPTS138::inversion insertion point 3 was used instead of pNPTS::inversion insertion point 2

Generation of Δsmc strains with inverted chromosome segment

We employed ΦCr30 generalized phage transduction (Ely, 1991) to move *Δsmc*::kanamycin^R or *Δsmc*::tetracycline^R alleles from the original deletion strains (ML2117 or ML2118) to the inversion strains.

For complementation of *Δsmc* strains with a plasmid-borne pML477::*flag-smc*, electroporation-competent *Caulobacter Δsmc* was transformed with pML477::*flag-smc* and plated out on PYE + spectinomycin. Colonies that formed on PYE + spectinomycin were restruck out on fresh PYE + spectinomycin plates twice to purify the strain.

Generation of Caulobacter *strains carrying rpoC-flag*

We employed ΦCr30 generalized phage transduction to move *rpoC::rpoC-flag::kanamycin^R* allele from the ML2299 strain (Haakonsen et al., 2015) to the wanted background to generate strains TLS1609, TLS1613.

Generation of Caulobacter *strains carrying cfp-parB and an ectopic parS at +1800 kb or +2242 kb*

We employed ΦCr30 generalized phage transduction to move 1800kb:*:parS^{Caulobacter}* (marked with tetracycline^R) from TLS1619 to MT190 (CB15N *parB::cfp-parB*) to result in strain TLS1620. Similarly, 2242kb::*parS^{Caulobacter}* (marked with tetracycline^R) from TLS1621 was transduced into MT190 (CB15N *parB::cfp-parB*) to result in strain TLS1622.

Generation of yeast two hybrid strains

A combination of pGBKT7-based plasmid and pGADT7-based plasmid was co-transformed into the yeast strain Y187 using the Frozen-EZ yeast transformation kit (ZymoResearch) and plated out on SD agar + leucine + tryptophan. The resulting colonies were restruck on SD agar + leucine + tryptophan plate to purify the strain before β-galactosidase assays.

Strains for labelling individual DNA loci:

For strain TLS1602 (CB15N *parS^{pMT1}*::200kb *parS^{P1}*::3842kb *dnaA*::P$_{van}$-*dnaA xyl*::P$_{xyl}$-*mcherry-parB^{P1}-yfp-parB^{pMT1}*): First, *parS^{pMT1}* at +200 kb (marked with spectinomycin^R) was transduced into *Caulobacter* strain *dnaA*::P$_{van}$-*dnaA*, then *parS^{P1}* at +3842 kb (marked with chloramphenicol^R) was transduced into the resulting strain. Finally, *xyl*::P$_{xyl}$-*mcherry-parB^{P1}-yfp-parB^{pMT1}* (marked with kanamycin^R) was transduced in. For strain TLS1605 (CB15N *parS^{pMT1}*::200kb *parS^{P1}*::3842kb *dnaA*::P$_{van}$-*dnaA xyl*::P$_{xyl}$-*mcherry-parB^{P1}-yfp-parB^{pMT1} Δsmc*::tetracycline^R): ΦCr30 generalized phage transduction was used to move *Δsmc*::tetracycline^R allele from the original deletion strains ML2117 to TLS1602.

For strain TLS1603 (CB15N *parS^{pMT1}*::1000kb *parS^{P1}*::3042kb *dnaA*::P$_{van}$-*dnaA* *xyl*::P$_{xyl}$-*mcherry-parB^{P1}-yfp-parB^{pMT1}*): First, *parS^{pMT1}* at +1000kb (marked with spectinomycin^R) was transduced into *Caulobacter* strain *dnaA*::P$_{van}$-*dnaA*, then *parS^{P1}* at +3042kb (marked with chloramphenicol^R) was transduced into the resulting strain. Finally, *xyl*::P$_{xyl}$-*mcherry-parB^{P1}-yfp-parB^{pMT1}* (marked with kanamycin^R) was transduced in. For strain TLS1606 (CB15N *parS^{pMT1}*::1000kb *parS^{P1}*::3042kb *dnaA*::P$_{lac}$-*dnaA* *xyl*::P$_{xyl}$-*mcherry-parB^{P1}-yfp-parB^{pMT1} Δsmc*::tetracycline^R): ΦCr30 generalized phage transduction was used to move *Δsmc*::tetracycline^R allele from the original deletion strains ML2117 to TLS1603.

For strain TLS1604 (CB15N *parS*^{pMT1}::1800kb *parS*^{P1}::2242kb *dnaA*::P$_{van}$-*dnaA* *xyl*::P$_{xyl}$-*mcherry-parB*^{P1}-*yfp-parB*^{pMT1}): First, *parS*^{pMT1} at +1800kb (marked with spectinomycin^{R}) was transduced into *Caulobacter* strain *dnaA*::P$_{van}$-*dnaA*, then *parS*^{P1} at +2242kb (marked with chloramphenicol^{R}) was transduced into the resulting strain. Finally, *xyl*::P$_{xyl}$-*mcherry-parB*^{P1}-*yfp-parB*^{pMT1} (marked with kanamycin^{R}) was transduced in. For strain TLS1607 (CB15N *parS*^{pMT1}::1800kb *parS*^{P1}::2242kb *dnaA*::P$_{lac}$-*dnaA* *xyl*::P$_{xyl}$-*mcherry-parB*^{P1}-*yfp-parB*^{pMT1} *Δsmc*::tetracycline^{R}): ΦCr30 generalized phage transduction was used to move *Δsmc*::tetracycline^{R} allele from the original deletion strains ML2117 to TLS1604.

For strain TLS1669 (CB15N *parS*^{pMT1}::1800kb *parS*^{P1}::1600kb *dnaA*::P$_{van}$-*dnaA* *xyl*::P$_{xyl}$-*mcherry-parB*^{P1}-*yfp-parB*^{pMT1}): First, *parS*^{pMT1} at +1800kb (marked with spectinomycin^{R}) was transduced into *Caulobacter* strain *dnaA*::P$_{van}$-*dnaA*, then *parS*^{P1} at +1600kb (marked with chloramphenicol^{R}) was transduced into the resulting strain. Finally, *xyl*::P$_{xyl}$-*mcherry-parB*^{P1}-*yfp-parB*^{pMT1} (marked with kanamycin^{R}) was transduced in. For strain TLS1670 (CB15N *parS*^{pMT1}::1800kb *parS*^{P1}::1600kb *dnaA*::P$_{lac}$-*dnaA* *xyl*::P$_{xyl}$-*mcherry-parB*^{P1}-*yfp-parB*^{pMT1} *Δsmc*::tetracycline^{R}): ΦCr30 generalized phage transduction was used to move *Δsmc*::tetracycline^{R} allele from the original deletion strains ML2117 to TLS1669.

Chromatin immunoprecipitation with deep sequencing (ChIP-seq) and generation of ChIP-seq profiles

Caulobacter cell cultures (25 mL) were grown in PYE to mid exponential phase (OD ~0.4) and fixed with formaldehyde to a final concentration of 1%. For ChIP-seq of FLAG-tagged SMC, mid-exponential phase cultures were washed twice in 1x PBS buffer before addition of formadehyde and crosslinker Gold (Diagenode) to a final concentration of 1%. Fixed cells were incubated at room temperature for 30 minutes, then quenched with 0.125 M glycine for 15 min at room temperature. Cells were washed three times with 1x PBS (pH 7.4) and resuspended in 1 mL of buffer 1 (20 mM K-HEPES pH 7.9, 50 mM KCl, 10% Glycerol and Roche EDTA-free protease inhibitors). Subsequently, the cell suspension was sonicated on ice using a Soniprep 150 probe-type sonicator (11 cycles, 15s ON, 15s OFF, at setting 8) to shear the chromatin to below 1 kb, and the cell debris was cleared by centrifugation (20 minutes at 13,000 rpm at 4°C).

The supernatant was then transferred to a new 2 mL tube and the buffer conditions were adjusted to 10 mM Tris-HCl pH 8, 150 mM NaCl and 0.1% NP-40. Fifty microliters of the supernatant were transferred to a separate tube for control (the INPUT fraction) and stored at -20°C. In the meantime, antibodies-coupled beads were washed off storage buffers before being added to the above supernatant. We employed α-GFP antibodies coupled to sepharose beads (Abcam, UK) for ChIP-seq of CFP-ParB, α-FLAG antibodies coupled to agarose beads (Sigma, UK) for ChIP-seq of RpoC-FLAG and FLAG-SMC. Briefly, 25 μL of α-GFP beads or 100 μL α-FLAG beads was washed off storage buffer by repeated centrifugation and resuspension in IPP150 buffer (10 mM Tris-HCl pH 8, 150 mM NaCl and 0.1% NP-40). Beads were then introduced to the cleared supernatant and incubated with gentle shaking at 4°C overnight. In the next day, beads were then washed five times at 4°C for 2 min each with 1 mL of IPP150 buffer, then twice at 4°C for 2 min each in 1x TE buffer (10 mM Tris-HCl pH 8 and 1 mM EDTA). Protein-DNA complexes were then eluted twice from the beads by incubating the beads first with 150 μL of the elution buffer (50 mM Tris-HCl pH 8, 10 mM EDTA and 1% SDS) at 65°C for 15 min, then with 100 μL of 1X TE buffer + 1% SDS for another 15 min at 65°C. The supernatant (the ChIP fraction) was then separated from the beads and further incubated at 65°C overnight to completely reverse crosslink. The INPUT fraction was also de-crosslinked by incubation with 200 μL of 1X TE buffer + 1% SDS at 65°C overnight. DNA from the ChIP and INPUT fraction were then purified using the PCR

purification kit (Qiagen) according to the manufacturer's instruction, then eluted out in 40 µL of a 10-fold-diluted EB buffer (Qiagen). The purified DNA was then used directly for qPCR or being made in to library suitable for Illumina sequencing using the NEXT Ultra library preparation kit (NEB). ChIP libraries were sequenced on the Illumina Hiseq 2500 at the Tufts University Genomics facility. For the list of ChIP-seq datasets in this study, see Supplementary Table S3.

For analysis of ChIP-seq data, Hiseq 2500 Illumina short reads (50 bp) were mapped back to the *Caulobacter* NA1000 reference genome (NCBI Reference Sequence: NC-011916.1) using Bowtie 1 (Langmead et al., 2009) using the following command:

*bowtie -m 1 -n 1 --best --strata -p 4 --chunkmbs 512 NA1000-2014-bowtie --sam *.fastq > output.sam*

For *Caulobacter* strains with an inverted DNA segment, a reconstructed fasta file with the correct orientation for the inverted segment was used as reference genome for Bowtie instead. Subsequently, sequencing reads were allocated to their corresponding 1-kb bins along the chromosome, and were normalized for the total number of reads to give the RPKPM value (number of reads per kb per million mapped reads). Finally, the profiles of SMC enrichment were plotted with the x-axis representing genomic positions and the y-axis is RPKPM from the ChIP fraction of FLAG-tagged SMC divided by RPKPM from the ChIP fraction of un-tagged SMC, using a custom R script.

Chromosome conformation capture with deep sequencing (Hi-C)
Hi-C experiments were performed exactly as described previously (Le et al., 2013). The restriction enzyme BglII were used for all Hi-C experiments in this study. Cells at OD_{600} of 0.2 were fixed with 1% formadehyde for 30 minutes before quenching by 0.125 M glycine. Fixed cells were washed twice in 1x M2 buffer before being resuspended in 1x TE buffer (10 mM Tris-HCl pH8 and 1 mM EDTA) and subjected to the Hi-C procedure (Le et al., 2013).

For Hi-C of ParB-depleted MT148 (Thanbichler and Shapiro, 2006), cells at mid-exponential phase were washed off xylose by repeated centrifugation and resuspension in fresh PYE before releasing to PYE+0.2% glucose. The culture was left shaking at 30°C for five more hours. The culture was adjusted with fresh PYE to OD_{600} of 0.2 before formadehyde was added to 1% final concentration to fix cells for Hi-C assay. Glycine (0.125M final concentration) was then added to quench the fixation. Fixed cells were washed twice in 1x M2 buffer before being resuspended in 1x TE buffer and subjected to the Hi-C procedure (Le et al., 2013).

Generation of Hi-C contacts maps and inter-arm Hi-C intensity plot
Each end of paired-end sequencing reads was mapped independently to the genome of *Caulobacter crescentus* NA1000 using Bowtie 2.1.0 and an algorithm used that iteratively increases truncation length to maximize the yield of valid Hi-C interactions (Imakaev *et al*, 2012). The *Caulobacter* NA1000 genome was then divided into restriction fragments (700 BglII fragments). Each read of a read pair was sorted into its corresponding restriction fragment. Read pairs were classified as valid Hi-C products, non-ligation, or self-ligation products (Imakaev et al., 2012). Only valid Hi-C products that are uniquely aligned to the *Caulobacter* genome were employed to generate Hi-C contact maps (Supplementary Table S3). To create interaction matrices, the *Caulobacter* genome was first divided into 405 10-kb bins. We then assigned valid Hi-C products to the 10-kb bins and normalised using an iterative correction procedure as described previously (Imakaev et al., 2012; Le et al., 2013). Subsequent analysis and visualization of the contact maps was done using R scripts. For *Caulobacter* strains with an inverted DNA segment, a reconstructed fasta file with the correct

orientation for the inverted segment was used as reference genome for Bowtie instead. All Hi-C contact maps in this manuscript report the log_e-scale of Hi-C interaction between 10-kb bins of DNA loci. Note that, in contrast to Hi-C maps reported previously (Le et al., 2013), here we use the logarithm of interaction frequencies to facilitate visualization of these weaker inter-arm interactions. The axes of Hi-C maps are oriented such as the origin of replication (*ori*: 0 kb or +4043 kb) is at the center of the x- and y-axis, and the left and the right chromosomal arm are on either side.

To determine the extent of inter-chromosomal-arm interactions, inter-arm Hi-C intensity was plotted against genomic positions (see also Fig. 1D). Inter-arm interaction was defined as Hi-C interactions between a 10-kb DNA bin and another bin equidistant from *ori* but on the opposite chromosomal arm. Essentially, this is the Hi-C interactions along the secondary diagonal spanning from the upper left corner to the lower right corner of each Hi-C contact map.

Calculate the rate of arm progression following ParB replenishment

First, the difference Hi-C maps between various time points (5, 10, 15, 20, 25 minutes after adding back xylose) and time point 0 were calculated by subtracting Hi-C maps at t5, t10, t15 or t25 from the t0 map. The procedure revealed the difference in DNA-DNA interactions between difference time points (Fig. S1E). The locator() function in R was then used to determine the genomic position of the leading edge of aligned DNA (black dashed lines in Fig. S1E), and the distance from the edge to *parS* site was calculated for each arm. We plotted the distance from *parS* to the leading edge against time points after adding xylose. We did not observe noticeable difference in arm progression between t0 and t5, therefore we used data from t10, t15, t20 and t25 to estimate the rate of arm progression. To estimate the rate, a linear regression line was fitted to these data. The rates of arm progression and the standard errors were calculated from the slope and its standard error of the best-fit lines.

Analyses of gene expression, RNA polymerase density and transcription orientation

RNA-seq data (Le and Laub, 2016) from CB15N *Caulobacter* growing at exponential phase in rich medium at 30°C were used to analyze the expression of genes that are oriented in the *ori-ter* or in the opposite direction i.e. *ter* to *ori*. For genes residing on the right arm of the chromosome (0 kb to +2000 kb), *ori-ter* oriented genes are on the plus strand (denoted with a + on a standard general feature fomat (gff) file) and *ter-ori* genes are on the minus strand (annotated as - as in the gff file). The opposite is true for genes on the left arm of the chromosome (+2000 kb to +4043 kb). The general feature format (gff) file for *Caulobacter* NA1000 was downloaded from NCBI (ftp://ftp.ncbi.nlm.nih.gov/genomes/Bacteria/*Caulobacter*-crescentus-NA1000-uid59307/), and used as a basis to separate genes to the plus or minus strand. Absolute expression value for each gene was calculated as the number of reads per million of mapped read (Le and Laub, 2016).

When using the enrichment of RpoC-FLAG ChIP-seq, only reads within the coding sequence i.e. excluding the promoter region, are used. This reflects the RNA polymerases that are elongating within the coding sequence instead of at the initiation phase at the promoter region. Pulled-down DNA from initiating RNA polymerases at promoter regions were discarded *in silico*. Again, genes are separated to the *ori-ter* or *ter-ori* direction as for the RNA-seq data above. Absolute RpoC enrichment value for each gene was calculated as the number of reads per kb per million of mapped read (RPKPM) * gene length (in kb). High expression genes are defined as genes with RPKPM*gene length values greater than 1000 (See Fig. 3A-B, Fig. 4A, and Fig. S6A and C).

Immunoblot analysis

For Western blot analysis, *Caulobacter* cells were pelleted and resuspended directly in 1x SDS sample buffer, then heated to 95°C for 5 min before loading. Total protein was run on 10% Tris-HCl gels (Bio-Rad) at 150 V for separation. Resolved proteins were transferred to polyvinylidene fluoride membranes using the Trans-Blot Turbo Transfer System (Biorad) and probed with 1:5,000 dilution of primary α-FLAG antibodies (Sigma-Aldrich), or 1:5,000 dilution of α-ParB antibody (custom antibody from CRB Cambridge, UK) and subsequently by a secondary horseradish peroxidase–conjugated antibody (1:10,000). Blots were imaged using an Amersham Imager 600 (GE Healthcare), and quantified using Image Studio Lite (LI-COR Biosciences).

Orthogonal ParB/*parS* system to label DNA loci and microscopy analysis

C. crescentus strains with $parS^{pMT1}$ or $parS^{P1}$ inserted at various locations on the chromosome was created as described above. These strains also harboured a P_{xyl}-m*cherry-parB*^{P1}-*yfp-parB*^{pMT1} cassette at the *xyl* locus. Strains were grown to OD_{600}=0.4 in the presence of appropriate antibiotics, vanillate, and glucose before the cells were collected by centrifugation and washed of residual vanillate, antibiotics and glucose twice with fresh PYE. Cells were then resuspended in PYE plus xylose (0.3% final concentration) (vanillate omitted) to deplete DnaA for 90 min before synchronization, thereby inducing cell elongation. Cells were imaged at 0 hr, 1 hr, 2 hr and 3 hr post synchronization. Phase contrast (150 ms exposure) and fluorescence images (2000 ms exposure) were collected. MicrobeTracker (http://microtracker.org) was used to detect cell outlines and SpotFinderZ to detect fluorescent foci positions (Sliusarenko et al., 2011). Only cells with a single Mcherry-$ParB^{P1}$ and YFP-$ParB^{pMT1}$ focus were used for construction of boxplots. Cells were then sorted into bins (<2.5 µm, 2.5-3.5 µm, 3.5-4.5 µm and 4.5-5.5 µm) according to their length. The number of cells used for construction of boxplots in Fig. 3 was as follows:

Labeled loci		< 2.5 µm	2.5-3.5 µm	3.5-4.5 µm	4.5-5.5 µm
200 kb-3842 kb	*smc*$^{+}$	232	915	593	351
	smc^{-}	218	1400	878	354
1000 kb-3042 kb	*smc*$^{+}$	177	922	697	396
	smc^{-}	179	1328	1246	746
1800 kb-2242 kb	*smc*$^{+}$	109	1059	855	414
	smc^{-}	118	1199	990	428
1600 kb-1800 kb	*smc*$^{+}$	85	868	903	495
	smc^{-}	106	1085	1112	616

Boxplots show the distribution of inter-foci distances for cells of different sizes were plotted using R programme. A one-tailed student's t-test was employed to test if inter-foci distances in *Δsmc* cells is greater than in wild-type cells.

Yeast-two hybrid assay

Yeast-two hybrid assays were performed exactly as described in the Clonetech manual. ParB was expressed as a fusion to the activation domain of Gal4 (AD), and $ParA^{WT}$ or $ParA^{K20R}$ as a fusion to the DNA-binding domain of Gal4 (BD). Four biological replicates were performed to obtain the mean and standard deviation of β-galactosidase activity.

TABLE S1, Related to the Experimental Procedures

Strains	Description	Source
***Caulobacter crescentus* strains**		
CB15N	Wild-type synchronizable *Caulobacter crescentus*	Lab collection
ML2000	CB15N *hfa::lacI dnaA::*P_{lac}-*dnaA*	Badrinarayanan et al., 2015
ML2413	ML2000 *xyl::*P_{xyl}-*parA*^{K20R}-*yfp* (gentamycin^{R})	Badrinarayanan et al., 2015
ML2414	ML2000 *xyl::*P_{xyl}-*parA*^{WT}-*yfp* (gentamycin^{R})	Badrinarayanan et al., 2015
ML2299	CB15N *rpoC::rpoC-flag*::kanamycin^{R}	Haakonsen et al., 2015
ML2118	CB15N *Δsmc*::kanamycin^{R}	Le et al., 2013
ML2117	CB15N *Δsmc*::tetracycline^{R}	Le et al., 2013
MT148	CB15N *parB::*P_{xyl}-*parB*	gift from Martin Thanbichler, Thanbichler and Shapiro, 2006
MT190	CB15N *parB::cfp-parB*	gift from Martin Thanbichler, Thanbichler and Shapiro, 2006
TLS1599	CB15N *Δsmc*::kanamycin^{R} pML477::*flag-smc*	
TLS1600	CB15N *ΔscpA*::kanamycin^{R}	This study
TLS1601	CB15N *ΔscpB*::markerless	This study
TLS1602	CB15N *parS*^{pMT1}::200 kb *parS*^{P1}::3842 kb *dnaA::*P_{van}-*dnaA xyl::*P_{xyl}-*mcherry-parB*^{P1}-*yfp-parB*^{pMT1}	This study
TLS1603	CB15N *parS*^{pMT1}::1000 kb *parS*^{P1}::3042 kb *dnaA::*P_{van}-*dnaA xyl::*P_{xyl}-*mcherry-parB*^{P1}-*yfp-parB*^{pMT1}	This study
TLS1604	CB15N *parS*^{pMT1}::1800 kb *parS*^{P1}::2242 kb *dnaA::*P_{van}-*dnaA xyl::*P_{xyl}-*mcherry-parB*^{P1}-*yfp-parB*^{pMT1}	This study
TLS1605	CB15N *parS*^{pMT1}::200 kb *parS*^{P1}::3842 kb *dnaA::*P_{van}-*dnaA xyl::*P_{xyl}-*mcherry-parB*^{P1}-*yfp-parB*^{pMT1}*Δsmc*::tetracycline^{R}	This study
TLS1606	CB15N *parS*^{pMT1}::1000 kb *parS*^{P1}::3042 kb *dnaA::*P_{van}-*dnaA xyl::*P_{xyl}-*mcherry-parB*^{P1}-*yfp-parB*^{pMT1}*Δsmc*::tetracycline^{R}	This study
TLS1607	CB15N *parS*^{pMT1}::1800 kb *parS*^{P1}::2242 kb *dnaA::*P_{van}-*dnaA xyl::*P_{xyl}-*mcherry-parB*^{P1}-*yfp-parB*^{pMT1}*Δsmc*::tetracycline^{R}	This study
TLS1669	CB15N *parS*^{pMT1}::1800 kb *parS*^{P1}::1600 kb *dnaA::*P_{van}-*dnaA xyl::*P_{xyl}-*mcherry-parB*^{P1}-*yfp-parB*^{pMT1}	This study

TLS1670	CB15N *parS*^{pMT1}::1800 kb *parS*^{P1}::1600 kb *dnaA::*P$_{van}$-*dnaA xyl::*P$_{xyl}$-*mcherry-parB*^{P1}-*yfp-parB*^{pMT1} *Δsmc*::tetracycline^{R}	This study
TLS1608	Flip 1-5: inversion between end point 1 (nt 4038432) and end point 5 (nt 3611123)	This study
TLS1609	Flip 1-5 *rpoC::rpoC-flag*::kanamycin^{R}	This study
TLS1610	Flip 1-5 *Δsmc*::kanamycin^{R}	This study
TLS1611	Flip 1-5 *Δsmc*::kanamycin^{R} pML477::*flag-smc*	This study
TLS1612	Flip 2-5: inversion between end point 2 (nt 4030311) and end point 5 (nt 3611123)	This study
TLS1613	Flip 2-5 *rpoC::rpoC-flag*::kanamycin^{R}	This study
TLS1614	Flip 2-5 *Δsmc*::kanamycin^{R}	This study
TLS1615	Flip 2-5 *Δsmc*::kanamycin^{R} pML477::*flag-smc*	This study
TLS1616	Flip 4-5: CB15N, inversion between end point 4 (nt 3788231) and end point 5 (nt 3611123)	This study
TLS1617	Flip 2-4 : CB15N, inversion between endpoint 2 (nt 4030311) and endpoint 4 (nt 3788231)	This study
TLS1618	Flip 3-4: CB15N, inversion between endpoint 3 (nt 3815825) and endpoint 4 (nt 3788231)	This study
TLS1619	CB15N 1800kb::*parS*^{Caulobacter} (260bp)	This study
TLS1620	CB15N *parB::cfp-parB* 1800kb::*parS*^{Caulobacter} (260bp)	This study
TLS1621	CB15N 2242kb::*parS*^{Caulobacter} (260bp)	This study
TLS1622	CB15N *parB::cfp-parB* 2242kb::*parS*^{Caulobacter} (260bp)	This study
TLS1623	ML2000 *Δsmc*::kanamycin^{R}	This study
**Yeast strains**		
	Y187	Clonetech
TLS1624	Y187 pGADT7-*parB* + pGBKT7-*parA*^{WT}	This study
TLS1625	Y187 pGADT7-*parB* + pGBKT7-*parA*^{K20R}	This study
TLS1626	Y187 pGADT7-*parB* + pGBKT7-empty	This study

TABLE S2, Related to the Experimental Procedures

**Plasmids**	**Description**	
pENTR-D-TOPO	ENTRY vector for gateway cloning, kanamycin^{R}	Invitrogen
pML477	Gateway-cloning destination vector for fusion of protein interest to an N-terminally FLAG tag, xylose-inducible promoter, medium-copy number plasmid, spectinomycin^{R}	Laub lab strain collection
pNPTS138	integrative vector for *Caulobacter* gene knockout/knock-in, kanamycin^{R}	Laub lab strain collection
pMCS2	integrative vector for *Caulobacter* gene knockout, kanamycin^{R}	Thanbichler et al., 2007
pNPTS138::*ΔscpB*	for the deletion of *scpB*	This study

pMCS2::*ΔscpA*	for the deletion of *scpA*	This study
pGADT7	vector for yeast-two hybrid assay, carbenicilin^{R}	Clonetech
pGBKT7	vector for yeast-two hybrid assay, carbenicilin^{R}	Clonetech
pGADT7::*parB*	expressing AD-ParB fusion protein, carbenicilin^{R}	This study
pBGKT7::*parA*^{K20R}	expressing DB-ParA fusion protein, carbenicilin^{R}	This study
pGBKT7::*parA*^{WT}	expressing DB-ParA^{K20R} fusion protein, carbenicilin^{R}	This study
pMT619:: *parS*^{pMT1} at +200kb	label +200 kb with *parS*^{pMT1} site, spectinomycin^{R}	This study
pMT632:: *parS*^{P1} at +3842kb	label +3842 kb with *parS*^{P1} site, chloramphenicol^{R}	This study
pMT619:: *parS*^{pMT1} at +1000kb	label +1000 kb with *parS*^{pMT1} site, spectinomycin^{R}	This study
pMT632: *parS*^{P1} at +3042kb	label +3042 kb with *parS*^{P1} site, chloramphenicol^{R}	This study
pMT619:: *parS*^{pMT1} at +1800kb	label +1800 kb with *parS*^{pMT1} site, spectinomycin^{R}	This study
pMT632:: *parS*^{P1} at +2242kb	label +2242 kb with *parS*^{P1} site, chloramphenicol^{R}	This study
pMCS5-*parS*^{Caulobacter}	plasmid for insertion of a second *Caulobacter parS* site (260 bp) at an ectopic location on the chromosome, tetracycline^{R}	This study
pMCS5:: *parS*^{Caulobacter} at +1800 kb	label +1800 kb with *Caulobacter parS* site (260 bp), tetracycline^{R}	This study
pMCS5:: *parS*^{Caulobacter} at +2242 kb	label +2242 kb with *Caulobacter parS* site (260 bp), tetracycline^{R}	This study
pNPTS138::inversion insertion point 1	for an insertion of *attP* (forward direction) at nucleotide (nt) position 4038385	This study
pNPTS138::inversion insertion point 2	for an insertion of *attB* (reverse direction) at nucleotide (nt) position 4030311	This study
pNPTS138::inversion insertion point 3	for an insertion of *attB* (reverse direction) at nucleotide (nt) position 3815825	This study
pNPTS138::inversion insertion point 4	for an insertion of *attP* (forward direction) at nucleotide (nt) position 3788231	This study
pNPTS138::inversion insertion point 5	for an insertion of *attB* (reverse direction) at nucleotide (nt) position 3611123	This study
pTORO78	pMT425-ΦC31, expressing *Streptomyces* ΦC31 integrase from a vanilate-inducible promoter, low-copy number plasmid, chloramphenicol^{R}	Gift from Lucy Shapiro, Toro et al 2008
pENTR::*smc*	entry vector harboring the coding sequence of SMC, kanamycin^{R}	This study
pML477:*flag-smc*	expressing flag-smc, spectinomycin^{R}	This study

**Primers**	**Sequence**	
**For the construction of pMCS2::*ΔscpA***		
scpA_500N_F	gctttcgcgagacgtccaattgcacacgggctttcagcccacctttgac	This study
scpA_500N_R	aactagtggatcccccgggctgcagcatcaggccatagaggtcgccctc	This study
**For the construction of pNPTS138::*ΔscpB***		
scpBfrag1_F	ccaagcttctctgcaggatatctggcctacctgaaatcgcgcctgctgctg	This study
scpBfrag1_R	gaaatcctgcgcgcagcgctcgacgaagagaggatcc	This study
scpBfrag2_F	gtcgagcgctgcgcgcaggatttcctgggcgagcccgaaaaag	This study
scpBfrag2_F	gagacgcgtcacggccgaagctagcgcggcggcgatgatgagaagttctgtg	This study
**For the construction of pENTR::*smc***		
smc-ENTR-D-TOPO-F	CACCgtgcagttccagcgcctccgcctg	This study
smc-ENTR-D-TOPO-R	ttaagccgccaccagcttctccgccg	This study
**For the construction of pMCS5:: *parS*^{Caulobacter}**		
CCparS_gibMCS5F	accttaagatctcgagctccggaggcccgggccctggagcgcatctccg	This study
CCparS_gibMCS5R	ctagcaccggtacgcgtaacgttcgcaagacgctcgcctcaatgcgaac	This study
**For the construction of pGADT7-*parB***		
parB-pGAD424_F	accaaacccaaaaaaagagatcgaattcggtggtggttccatgtccgaagggcgtcgtggtc	This study
parB-pGAD424_R	tcatagatctctgcaggtcgacggatccctcagatcccgcgcgtcagtcggttg	This study
**For the construction of pGBKT7-*parA*^{WT} and pGBKT7-*parA*^{K20R}**		
parA-pGBT_F	aaagacagttgactgtatcgccggaattcggtggtggttccgtgtccgctaatcctctccgcg	This study

parA-pGBT_R	attagcttggctgcaggtcgacggatcccttaggcggccttggcctggc gatcg	This study
**For the construction of pMT619::*parS*^{pMT1}**		
label200-NdeI-F	CATATGatcgaaaagaccttcaagctg	This study
label200-SacI-R	GAGCTCtcacgcctttcccatatagatgaac	This study
label1000-NdeI-F	CATATGttgggcttaggtgtggaccacg	This study
label1000-SacI-R	GAGCTCctaccgccgcttcaacttcgccag	This study
label1800-NdeI-F	CATATGctgccgatgacggaggcggcctac	This study
label1800-SacI-R	GAGCTCtcatggacgggcgctcccgtgac	This study
**For the construction of pMT632::*parS*^{P1}**		
label2242-NdeI-F	CATATGgtgaacggacaagtgggggaacac	This study
label2242-SacI-R	GAGCTCtcaggcggggaacatcctcgccag	This study
label3042-NdeI-F	CATATGatcagacccaccctgaccgcc	This study
label3042-SacI-R	GAGCTCctactcctccaccccatgggcgtag	This study
label3842-NdeI-F	CATATGttgcgtaacggcgaactgcggcgc	This study
label3842-SacI-R	GAGCTCttactgacgcgccttggccacc	This study

Uppercase letters denote restriction enzyme recognition site or sequence required for Gateway TOPO cloning

TABLE S3, Related to the Experimental Procedures

**Hi-C datasets**	**Restriction enzymes**	**GEO**
CB15N in PYE, synchronized	BglII	GSE45966
ML2118 in PYE, synchronized	BglII	GSE45966
TLS1612, synchronized, rep 1	BglII	This study GSE97330
TLS1612, synchronized, rep 2	BglII	This study GSE97330
TLS1614, synchronized	BglII	This study GSE97330
TLS1612 + 25 µg/ml rifampicin for 30min, synchronized	BglII	This study GSE97330
TLS1608, synchronized, rep 1	BglII	This study GSE97330
TLS1608, synchronized, rep 2	BglII	This study GSE97330
TLS1608, synchronized, rep 3	BglII	This study GSE97330
TLS1610, synchronized	BglII	This study

		GSE97330
TLS1616, synchronized	BglII	This study GSE97330
TLS1616 + 25 µg/ml rifampicin for 30min, synchronized	BglII	This study GSE97330
TLS1617, synchronized	BglII	This study GSE97330
TLS1617 + 25 µg/ml rifampicin for 30min, synchronized	BglII	This study GSE97330
TLS1618, synchronized	BglII	This study GSE97330
TLS1618 + 25 µg/ml rifampicin for 30min, synchronized	BglII	This study GSE97330
ML2413 + xylose (0.3%) for 1 hr, rep1	BglII	This study GSE97330
ML2413 + xylose (0.3%) for 1 hr, rep2	BglII	This study GSE97330
ML2414 + xylose (0.3%) for 1 hr, rep1	BglII	This study GSE97330
ML2414 + xylose (0.3%) for 1 hr, rep2	BglII	This study GSE97330
MT148 + glucose (0.2%) for 5 hrs	BglII	This study GSE97330
MT148 ParB depletion, 5hr after xylose withdrawal, rep 1	BglII	This study GSE97330
MT148 ParB depletion, 5hr after xylose withdrawal, rep 2	BglII	This study GSE97330
MT148 ParB replenishment, 5 min after adding xylose (0.3%)	BglII	This study GSE97330
MT148 ParB replenishment, 10 min after adding xylose (0.3%)	BglII	This study GSE97330
MT148 ParB replenishment, 15 min after adding xylose (0.3%)	BglII	This study GSE97330
MT148 ParB replenishment, 20 min after adding xylose (0.3%)	BglII	This study GSE97330
MT148 ParB replenishment, 25 min after adding xylose (0.3%)	BglII	This study GSE97330
TLS1599, 0 min after adding xylose (0.3%)	BglII	This study GSE97330
TLS1599, 15 min after adding xylose (0.3%)	BglII	This study GSE97330
TLS1599, 30 min after adding xylose (0.3%)	BglII	This study GSE97330
TLS1599, 60 min after adding xylose (0.3%)	BglII	This study GSE97330
TLS1600, synchronized, rep 1	BglII	This study GSE97330
TLS1600, synchronized, rep 2	BglII	This study GSE97330

TLS1601, synchronized	BglII	This study GSE97330
TLS1619, synchronized	BglII	This study GSE97330
TLS1619, mixed population	BglII	This study GSE97330
TLS1621, synchronized	BglII	This study GSE97330
CB15N + 25 µg/ml rifampicin for 30min, synchronized, rep 1	BglII	This study GSE97330
CB15N + 25 µg/ml rifampicin for 30min, synchronized, rep 2	BglII	This study GSE97330
CB15N + 25 µg/ml rifampicin for 30min, synchronized, rep 3	BglII	This study GSE97330
CB15N, synchronized, rep 1	BglII	This study GSE97330
CB15N, synchronized, rep 2	BglII	This study GSE97330
CB15N, synchronized, rep 3	BglII	This study GSE97330
ML2000, 90 minutes after IPTG withdrawal, synchronized	BglII	GSE74364
ML2000, 150 minutes after IPTG withdrawal, synchronized	BglII	GSE74364
ML2000, 210 minutes after IPTG withdrawal, synchronized	BglII	GSE74364
ML2000, 270 minutes after IPTG withdrawal, synchronized	BglII	GSE74364
TLS1623, 90 minutes after IPTG withdrawal, synchronized	BglII	GSE74364
TLS1623, 210 minutes after IPTG withdrawal, synchronized	BglII	GSE74364

**ChIP-seq datasets**	**GEO**
CB15N, fixation with 1% formadehyde and crosslinker Gold (Diagenode), α-FLAG antibody (Sigma), ChIP fraction	This study GSE97330
TLS1599, fixation with 1% formadehyde and crosslinker Gold (Diagenode), α -FLAG antibody (Sigma), ChIP fraction	This study GSE97330
TLS1608, fixation with 1% formadehyde and crosslinker Gold (Diagenode), α -FLAG antibody (Sigma), ChIP fraction	This study GSE97330
TLS1611, fixation with 1% formadehyde and crosslinker Gold (Diagenode), α -FLAG antibody (Sigma), ChIP fraction	This study GSE97330
TLS1612, fixation with 1% formadehyde and crosslinker Gold (Diagenode), α-FLAG antibody (Sigma), ChIP fraction	This study GSE97330
TLS1615, fixation with 1% formadehyde and	This study

crosslinker Gold (Diagenode), α-FLAG antibody (Sigma), ChIP fraction	GSE97330
ML2299, fixation with 1% formadehyde, α-FLAG antibody (Sigma), ChIP fraction	This study GSE97330
TLS1609, fixation with 1% formadehyde, α-FLAG antibody (Sigma), ChIP fraction	This study GSE97330
TLS1613, fixation with 1% formadehyde, α-FLAG antibody (Sigma), ChIP fraction	This study GSE97330
MT190, fixation with 1% formadehyde, α-GFP antibody (Abcam), ChIP fraction	This study GSE97330
TLS1620, fixation with 1% formadehyde, α-GFP antibody (Abcam), ChIP fraction	This study GSE97330
TLS1622, fixation with 1% formadehyde, α-GFP antibody (Abcam), ChIP fraction	This study GSE97330
CB15N, fixation with 1% formadehyde, α-FLAG antibody (Sigma), ChIP fraction	This study GSE97330

SUPPLEMENTARY REFERENCES

Badrinarayanan, A., Le, T.B.K., and Laub, M.T. (2015). Rapid pairing and resegregation of distant homologous loci enables double-strand break repair in bacteria. J. Cell Biol. *210*, 385–400.

Ely, B. (1991). Genetics of Caulobacter crescentus. Methods Enzym. *204*, 372–384.

Haakonsen, D.L., Yuan, A.H., and Laub, M.T. (2015). The bacterial cell cycle regulator GcrA is a σ70 cofactor that drives gene expression from a subset of methylated promoters. Genes Dev. *29*, 2272–2286.

Imakaev, M., Fudenberg, G., McCord, R.P., Naumova, N., Goloborodko, A., Lajoie, B.R., Dekker, J., and Mirny, L.A. (2012). Iterative correction of Hi-C data reveals hallmarks of chromosome organization. Nat Methods *9*, 999–1003.

Kahng, L.S., and Shapiro, L. (2003). Polar localization of replicon origins in the multipartite genomes of Agrobacterium tumefaciens and Sinorhizobium meliloti. J.Bacteriol. *185*, 3384–3391.

Langmead, B., Trapnell, C., Pop, M., and Salzberg, S.L. (2009). Ultrafast and memory-efficient alignment of short DNA sequences to the human genome. Genome Biol *10*, R25.

Le, T.B., and Laub, M.T. (2016). Transcription rate and transcript length drive formation of chromosomal interaction domain boundaries. EMBO J. *35*, 1582–1595.

Le, T.B., Imakaev, M.V., Mirny, L.A., and Laub, M.T. (2013). High-resolution mapping of the spatial organization of a bacterial chromosome. Science *342*, 731–734.

Skerker, J.M., Prasol, M.S., Perchuk, B.S., Biondi, E.G., and Laub, M.T. (2005). Two-component signal transduction pathways regulating growth and cell cycle progression in a bacterium: a system-level analysis. PLoS Biol *3*, e334.

Sliusarenko, O., Heinritz, J., Emonet, T., and Jacobs-Wagner, C. (2011). High-throughput, subpixel precision analysis of bacterial morphogenesis and intracellular spatio-temporal dynamics. Mol Microbiol *80*, 612–627.

Thanbichler, M., and Shapiro, L. (2006). MipZ, a Spatial Regulator Coordinating Chromosome Segregation with Cell Division in Caulobacter. Cell *126*, 147–162.

Toro, E., Hong, S.-H., McAdams, H.H., and Shapiro, L. (2008). Caulobacter requires a dedicated mechanism to initiate chromosome segregation. Proc. Natl. Acad. Sci. *105*, 15435–15440.
